# Parathyroid hormone–related protein is a therapeutic target in idiopathic pulmonary fibrosis

**DOI:** 10.1038/s41392-026-02578-8

**Published:** 2026-02-23

**Authors:** Xue-Quan Fang, Suha Lim, Yoon-Mi Lee, Chang-Hoon Lim, Han-Byeol Kim, Jeong Ho Joo, Sang-Woo Han, Seohyun Kim, Ji Hyung Kim, Kwon Joong Na, Samina Park, Young Tae Kim, Jimyung Park, Jooho Park, Jeong Seok Lee, Eun-Young Shin, Eung-Gook Kim, Hyun-Woo Shin, Ji-Hong Lim

**Affiliations:** 1 Department of Medicinal Biosciences, College of Biomedical and Health Science, Konkuk University, Chungju, Republic of Korea; 2 Department of Applied Life Science, Graduate School, BK21 Program, Konkuk University, Chungju, Republic of Korea; 3 Center for Metabolic Diseases, Konkuk University, Chungju, Republic of Korea; 4 Obstructive Upper Airway Research (OUaR) Laboratory, Department of Pharmacology, Seoul National University College of Medicine, Seoul, Republic of Korea; 5 Department of Biomedical Sciences, Seoul National University Graduate School, Seoul, Republic of Korea; 6 Sensory Organ Research Institute, Seoul National University Medical Research Center, Seoul, Republic of Korea; 7 Department of Biochemistry, Chungbuk National University College of Medicine, Cheongju, Republic of Korea; 8 Graduate School of Medical Science and Engineering, Korea Advanced Institute of Science and Technology, Daejeon, Republic of Korea; 9 Department of Biotechnology, College of Biomedical & Health Science, Konkuk University, Chungju, Chungbuk Republic of Korea; 10 Department of Biotechnology, College of Life Sciences and Biotechnology, Seoul, Korea University Republic of Korea; 11 Department of Thoracic and Cardiovascular Surgery, Seoul National University Hospital, Seoul National University College of Medicine, Seoul, Republic of Korea; 12 Cancer Research Institute, Seoul National University College of Medicine, Seoul, Republic of Korea; 13 Division of Pulmonary and Critical Care Medicine, Department of Internal Medicine, Seoul National University Hospital, Seoul National University College of Medicine, Seoul, Republic of Korea; 14 Department of Otorhinolaryngology-Head and Neck Surgery, Seoul National University Hospital, Seoul, Republic of Korea

**Keywords:** Molecular medicine, Respiratory tract diseases

## Abstract

The crosstalk between immune or alveolar epithelial cells and fibroblasts mediated by paracrine signaling molecules is associated with the pathogenesis of idiopathic pulmonary fibrosis (IPF). However, studies investigating the active involvement of soluble mediators derived from bronchial epithelial cells in fibroblast activation and the development of pulmonary fibrosis are limited. Reanalysis of bulk and single-cell RNA-sequencing data from human bronchus and IPF lung tissue revealed marked upregulation of parathyroid hormone–like hormone (*PTHLH*) in IPF lung tissue compared with normal tissue, with expression predominantly localized to bronchial epithelial cells. parathyroid hormone–related protein (PTHrP) translated from *PTHLH* was significantly increased in the bronchial epithelium of IPF patients and bleomycin-induced pulmonary fibrosis mice. Furthermore, the paracrine peptide PTHrP_1-34_, generated through post-translational processing of PTHrP, was elevated in lung homogenates and bronchoalveolar lavage fluid obtained from fibrotic lungs. Cell- and animal-based experiments showed that PTHrP_1-34_ activated fibroblasts and extracellular matrix production, resulting in the progression of pulmonary fibrosis. In a preclinical evaluation using a bleomycin-induced pulmonary fibrosis mouse model, attenuating effects against pulmonary fibrosis were observed using neutralizing antibodies, peptides, and gene silencing strategies targeting the PTHrP_1-34_/parathyroid hormone 1 receptor axis. In conclusion, our results suggest that PTHrP_1-34_ derived from bronchial epithelial cells is involved in the pathogenesis of IPF and is a promising target for alleviating disease progression.

## Introduction

Idiopathic pulmonary fibrosis (IPF) is a prototypical chronic and progressive fibrotic lung disease marked by reduced lung compliance, impaired gas exchange, and ultimately respiratory failure and death.^1^ Genetic predisposition significantly contributes to IPF susceptibility, with variants in genes such as *TERT*, *TERC*, and *MUC5B* strongly associated with disease risk. In addition to genetic factors, recent studies have highlighted the lung microbiome’s composition and dynamics as key modulators of disease progression.^2^ Central to IPF pathogenesis is a persistent inflammatory milieu, wherein soluble mediators such as interleukin (IL)-1β, IL-11, IL-6, transforming growth factor beta 1 (TGFβ1), and tumor necrosis factor-alpha (TNF-α) drive fibrotic remodeling.^3,4^ Mechanistically, IPF is characterized by excessive deposition of extracellular matrix (ECM) components, particularly collagen, resulting from the aberrant activation and survival of fibroblasts that differentiate into myofibroblasts. These myofibroblasts, defined by the expression of ACTA2 encoding alpha-smooth muscle actin (α-SMA), play a pivotal role in the fibrotic response to epithelial injury.^5,6^

Despite advances in our understanding of IPF pathobiology, therapeutic options remain limited. Currently, the only approved agents are pirfenidone and nintedanib, which slow disease progression^7^ but do not stop or reverse functional decline, do not improve quality of life, and are often associated with tolerability concerns.^8^ Furthermore, several recent clinical trials investigating novel therapeutic candidates have not demonstrated significant clinical efficacy.^3^ Therefore, the continued exploration of new therapeutic strategies and druggable targets for the effective diagnosis and treatment of IPF is urgently needed.

Parathyroid hormone–related protein (PTHrP), encoded by the parathyroid hormone–like hormone (*PTHLH*) gene, functions as a global endocrine hormone. Locally, PTHrP exerts paracrine, autocrine, and intracrine activity that regulates cell growth and apoptosis, calcium homeostasis, and epithelial-mesenchymal transition (EMT) during the development of bone, lung, and mammary glands.^9–11^ Similar to prohormones, PTHrP is cleaved at multibasic endoproteolytic sites into multiple smaller secretory peptides, including PTHrP_1-34_, PTHrP_38-94_, PTHrP_38-101_, and PTHrP_107-139_—all of which possess physiological activity—through post-translational processing of proteins.^12,13^ PTHrP_1-34_ regulates multiple signaling pathways and exerts different physiological effects as a ligand of the parathyroid hormone 1 receptor (PTH1R), with an affinity equal to that of parathyroid hormone (PTH).^14^ PTH1R, a class B1 G protein-coupled receptor, regulates signaling pathways mediated by cyclic AMP (cAMP), protein kinase A (PKA), protein kinase C, intracellular calcium, and nitric oxide (NO) in multiple cell types1.^15^ Among all regions of PTHrP, only the PTHrP_1-34_ region is biologically active in PTH1R-mediated signaling pathways.^16,17^

In the respiratory airway system, PTHrP is expressed in both fetal and adult lungs and is involved in lung development and pathophysiology, including lung injury and cancer.^9^ A comparative study in patients with or without acute lung injury (ALI) receiving mechanical ventilation showed that PTHrP levels in the bronchoalveolar lavage fluid (BALF) were approximately twofold higher in patients with ALI than in normal controls, although the difference was not statistically significant and was negatively correlated with injury severity.^18^ Likewise, some studies have reported biological functions of PTHrP in the remodeling of lung injury and development, the clinical significance of PTHrP in pulmonary fibrosis (PF), a severe lung disease, remains unknown.

In this study, we reanalyzed single-cell RNA-sequencing (scRNA-seq) data derived from human bronchus and IPF lung tissue. We investigated the mechanistic role of PTHrP_1-34_ in pulmonary fibrosis both in vitro and using in vivo models and proposed different strategies for blocking IPF progression.

## Results

### Integrative transcriptomic analysis identifies PTHrP as a novel secretory factor upregulated in IPF lungs

**Fig. 1:**
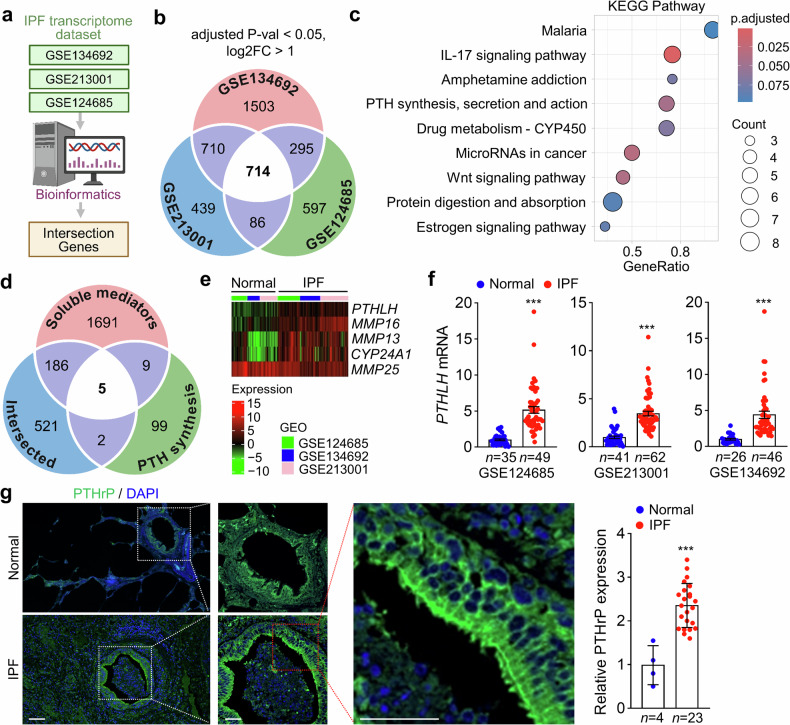
PTHrP expression in IPF and BLM-induced PF in humans. **a** Procedure for bioinformatics-based transcriptome analysis. **b** Identification of 714 commonly up- or downregulated genes in human IPF lungs using publicly available transcriptome datasets. **c** Top 9 activated gene sets identified by KEGG pathway analysis based on 714 common genes. **d** Identification of 5 genes through the intersection of genes related to soluble mediators, PTH synthesis, secretion, and action and 714 common genes. **e** Heatmap of *PTHLH* expression in normal and IPF samples. **f** *PTHLH* mRNA in normal and IPF samples. **g** Representative images of IF staining of PTHrP and quantification of the intensity of expression of PTHrP in human pulmonary interstitial fibrosis tissue microarrays from patients with IPF (*n* = 23) and healthy donors (*n* = 4). A magnified view of the region highlighted in the red box is shown. Scale bar: 50 μm and 100 μm (low magnification). **a**, **b**, **d** were created with BioRender.com. Data are shown as the mean ± SEM. *P* values were determined by two-tailed Student’s *t* test (**f**, **g**). ***P < 0.001

Many therapeutic strategies inhibiting secreted factors that promote fibrosis progression in preclinical models have been assessed in humans; however, further development and assessment of secreted factors for therapeutic intervention are crucial for extending the therapeutic opportunities for patients with IPF.^3^ To identify novel secreted factors that increase predominantly in the lungs of patients with IPF, we initially surveyed 3 different publicly available transcriptome datasets and identified 714 intersecting up- or downregulated genes (Fig. 1a, b). Among the 714 intersecting genes identified from transcriptomic datasets, Kyoto Encyclopedia of Genes and Genomes pathway analysis revealed statistically significant enrichment (adjusted *p* < 0.05) in gene sets associated with the IL-17 signaling pathway; PTH synthesis, secretion, and action; Wnt signaling; and microRNAs in cancer (Fig. 1c). Given the well-established clinical relevance of the IL-17 and Wnt signaling pathways in IPF and their roles as molecular targets for therapeutic development,^19^ we focused on identifying novel secretory factors potentially involved in IPF pathogenesis. To this end, we performed an intersectional analysis of the 714 differentially expressed genes in IPF with two curated datasets: 115 genes related to PTH synthesis, secretion, and action, and 1891 predicted secretory proteins based on The Human Protein Atlas (Fig. 1d). This integrative approach enabled the identification of previously unrecognized secretory factors that may contribute to IPF progression. Notably, we observed the upregulation of several genes encoding secretory proteins in human IPF lung tissues, including *PTHLH*, *MMP16*, *MMP13*, and *CYP24A1* (Fig. 1e). An increase in *PTHLH* mRNA was observed in IPF lungs compared with healthy lungs (Fig. 1f). To measure the PTHrP encoded by *PTHLH*, immunofluorescence (IF) staining was performed on lung sections from patients with IPF. The low-magnification image showed that the expression of the PTHrP protein was higher in IPF lung tissues than in normal lung tissues. In addition, the enlarged image (red box) in Fig. 1g shows that PTHrP was predominantly expressed in the bronchial epithelium of the lung tissue from patients with IPF. Analysis of PTHrP expression intensity revealed that its expression was approximately 2.5-fold higher in IPF lung tissues than in normal lung tissues (Fig. 1g). These findings indicate that both *PTHLH* mRNA and PTHrP protein levels are elevated in lung tissues from patients with IPF.

### Tissue-specific expression of PTHLH and PTH1R highlights a bronchial epithelial–mesenchymal interaction axis in PF

**Fig. 2:**
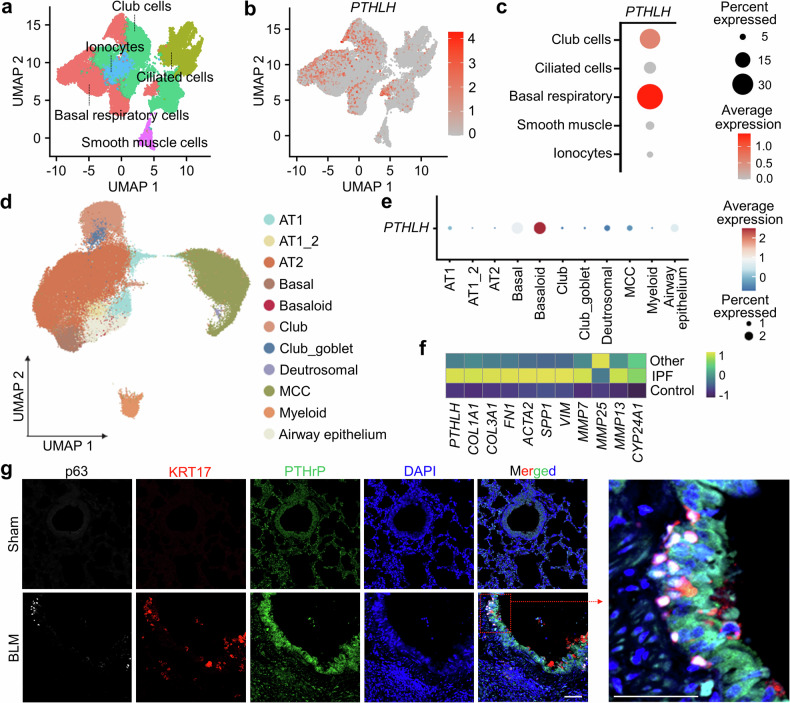
Cell type-specific expression of PTHrP in epithelial cells of the bronchus and IPF lungs. **a** Visualization of uniform manifold approximation and projection (UMAP) of bronchial epithelial cell populations (*n* = 26,676) in scRNA-seq data from human bronchi. **b** UMAP visualization of single cells expressing *PTHLH* (red) in bronchial epithelial cell populations. **c** Dot plot representing the expression of *PTHLH* among annotated bronchial epithelial cells of the human bronchus. **d** UMAP visualization of subclustered cell types belonging to lung epithelial cells (*n* = 91,443) in IPF lungs; scRNA-seq data from IPF lungs are colored according to subclustered cells. **e** Dot plot representing the expression of *PTHLH* among annotated alveolar and bronchial epithelial cells. The dot size indicates the percentage of relative gene expression for each marker gene. The dot color reflects the average expression of the specified gene within each cell type. **f** Heatmap of fibrosis genes associated with different disease conditions. **g** Representative IF staining shows colocalization of the aberrant basaloid cell markers KRT17 (red) and p63 (white) along with PTHrP (green). Representative images were captured at 200x magnification. Scale bar: 100 μm. A magnified view of the region highlighted in the red box is shown. AT1 Alveolar type 1, AT1_2 Alveolar type 1_2, AT2 Alveolar type 2, MCC Multiciliate cell

To confirm the predominance of tissue-specific expression of *PTHLH* in human tissues, we reanalyzed publicly available single-cell transcriptome data provided by The Human Protein Atlas. Interestingly, higher expression of *PTHLH* was observed in basal respiratory cells and club cells, which are the main components of the bronchial epithelium, than in other cell types. However, *PTHLH* expression was not observed in either type 1 (AT1) or 2 (AT2) alveolar cells that compose the alveoli (Fig. 2a–c and Supplementary Fig. 1a). Single-cell analysis of *PTHLH* expression in human IPF lungs revealed the highest expression in basaloid cells (KRT5-, KRT17 + , and LAMB3 + ),^20^ with elevated levels also observed in basal respiratory cells and in the airway epithelium (Fig. 2d, e). Compared with that in healthy controls, the expression of *PTHLH*, along with genes associated with fibrosis, such as *COL1A1*, *COL3A1*, and *ACTA2*, was upregulated in IPF lungs (Fig. 2f). PTHrP expression was markedly increased in the bronchial epithelium of bleomycin (BLM)-induced fibrotic lungs, and co-expression of PTHrP with p63 and KRT17 (markers for aberrant basaloid cells) was also observed in a portion of the bronchial epithelial cell population, which was not detected in normal lungs (Fig. 2g and Supplementary Fig. 1b, c). Notably, approximately 70% of the p63+ and KRT17+ cell populations also expressed PTHrP, indicating a strong association between aberrant basaloid cell identity and PTHrP expression in fibrotic lungs (Supplementary Fig. 1d). Furthermore, PTHrP was predominantly expressed in bronchial epithelial cells, including aberrant basaloid cells, in BLM-induced fibrotic lungs, suggesting its potential involvement in the pathophysiology of PF. We reanalyzed the expression of *PTH1R* and parathyroid hormone 2 receptor (*PTH2R*), known receptors for PTHrP and PTH, respectively, at the single-cell level using the scRNA-seq data from the lung.^21^ Interestingly, *PTH1R* and *PTH2R* exhibited the highest expression in kidney tubular cells (Supplementary Fig. 2a, b). Among the cell types implicated in the pathogenesis of PF, *PTH1R* was predominantly expressed in smooth muscle cells and fibroblasts (Supplementary Fig. 2c–e). Collectively, these findings suggest a potential interaction between bronchial epithelial cells and mesenchymal cells, including bronchial smooth muscle cells and lung fibroblasts, through the PTHrP/PTH1R regulatory axis.

### Cell type-specific activation of fibroblasts by PTHrP_1-34_ through PTH1R/PKA signaling

**Fig. 3:**
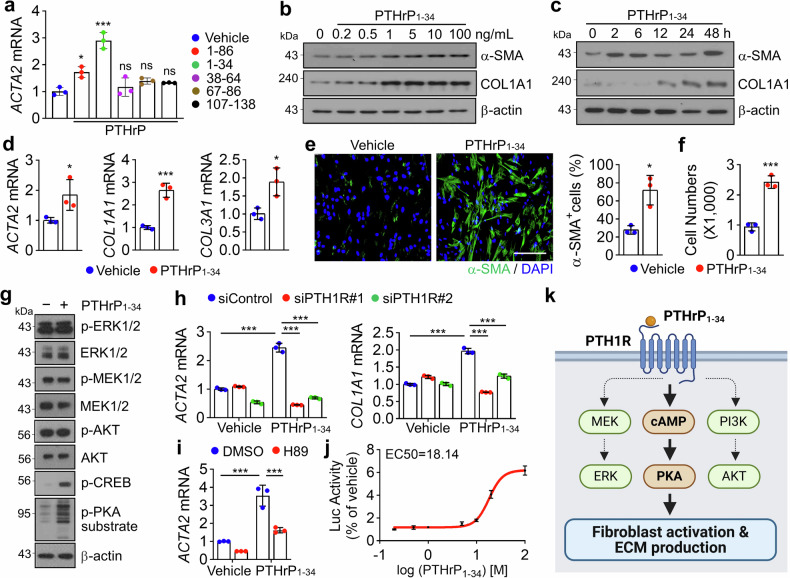
PTHrP_1-34_ induces the activation of lung fibroblasts via PTH1R/PKA signaling. **a** Expression of *ACTA2* mRNA in MRC5 cells treated with PTHrP peptides (aa 1-86, 1-34, 38–64, 67–86, or 107–138). Peptides (100 ng/mL) were incubated for 48 h. **b** Fibrosis-related proteins (α-SMA and COL1A1) in MRC5 cells treated with PTHrP_1-34_ in a dose-dependent manner for 48 h. **c** Fibrosis-related proteins (α-SMA and COL1A1) in MRC5 cells treated with PTHrP_1-34_ (100 ng/mL) in a time-dependent manner. **d** Expression of fibrosis-related genes in human IPF fibroblasts following incubation with PTHrP_1-34_ (100 ng/mL) for 24 h. **e** Representative IF images of α-SMA (green) in MRC5 cells incubated with PTHrP_1-34_ (100 ng/mL, 24 h) and quantification of α-SMA-positive cells. Scale bar: 100 μm. **f** Quantification of MRC5 cell migration following PTHrP_1-34_ (100 ng/mL) treatment for 24 h. **g** Western blot analysis of MAPK, PKA and AKT signaling pathways in MRC5 cells treated with PTHrP_1-34_ (100 ng/mL) for 10 min. **h** Expression of fibrosis-related genes in MRC5 cells transiently transfected with siRNA (20 nM) targeting control (siControl) or PTH1R (siPTH1R#1 and siPTH1R#2), followed by treatment with PTHrP_1-34_ (100 ng/ml) for 48 h. **i** *ACTA2* mRNA levels in MRC5 cells pretreated with the PKA inhibitor H89 (10 μM) for 1 h prior to PTHrP_1-34_ treatment (100 ng/mL, 48 h). **j** The EC_50_ value of the PTHrP_1-34_ was determined based on CRE-luciferase activity in HEK293-PTH1R cells. **k** Schematic representation of the expected intracellular signaling cascades activated in fibroblasts following PTHrP_1-34_ stimulation. All data shown are the mean ± SEM. *P* values were determined by two-tailed Student’s *t*-test in (**d**–**f**) and one-way ANOVA Tukey’s test in (**a**, **h**, **i**). *P < 0.05, ***P < 0.001. ns not significant

PTHrP undergoes proteolytic processing to generate multiple types of fragments, which exert diverse biological functions through both paracrine and endocrine mechanisms.^12,13^ Based on this, we investigated the role of distinct PTHrP fragments in mediating intercellular communication between bronchial epithelial cells and mesenchymal cells, such as smooth muscle cells and fibroblasts. Treatment of MRC5 lung fibroblasts with PTHrP fragments containing amino acids 1–34, such as PTHrP_1-34_ and PTHrP_1-86_, significantly upregulated *ACTA2* expression, whereas fragments lacking this region had no effect, underscoring the functional importance of the 1–34 domain in myofibroblast activation (Fig. 3a). Treatment with PTHrP_1-34_ increased the expression of fibrosis-related genes and proteins in a dose- and time-dependent manner in MRC5 cells (Fig. 3b, c and Supplementary Fig. 3a), human primary lung fibroblasts derived from a patient with IPF (Fig. 3d and Supplementary Fig. 3b), and mouse primary lung fibroblasts (Supplementary Fig. 3c). PTHrP_1-34_ treatment increased the population of α-SMA-positive cells (Fig. 3e) and enhanced the motility of MRC5 cells (Fig. 3f and Supplementary Fig. 3d). From a structural perspective on the interaction between PTHrP and PTH1R, the N-terminal region of PTHrP (amino acids 1–14) is deeply inserted into the transmembrane domain (TMD) of PTH1R, ultimately activating the stimulatory G protein (Gs) signaling pathway, while the C-terminal region (amino acids 15–34) binds to the extracellular domain (ECD) of PTH1R, contributing to binding affinity and ligand specificity.^16,22–24^ Consistent with previous study results, our AlphaFold3-based structural analysis revealed that the N-terminal region of PTHrP_1-34_ inserts into the binding pocket within the TMD core of PTH1R, forming extensive interactions (Supplementary Fig. 3e). In particular, residues E4, R19, and R21 of PTHrP engage in hydrogen bonding and electrostatic interactions with key residues in the TMD of PTH1R, including Y195, R233, E35, E252, E177, and E180 (Supplementary Fig. 3f). Additionally, hydrophobic residues in the C-terminal region of PTHrP_1-34_, such as F23, L24, L27, I28, and I31, form broad hydrophobic contacts with the ECD of PTH1R, notably with residues I38, I115, I135, and F138 (Supplementary Fig. 3g). These findings suggest that the activation of fibroblasts via PTH1R is dependent on the region encompassing amino acids 1–34 of PTHrP, highlighting the functional significance of this fragment in receptor engagement and downstream signaling. Based on the known involvement of PTH1R in mitogen-activated protein kinase (MAPK), PKA, and AKT signaling pathways across various cell types,^25,26^ we investigated the key intracellular signaling cascades mediating fibroblast activation in response to PTHrP_1-34_. In MRC5 fibroblasts treated with PTHrP_1-34_, we observed a marked increase in the phosphorylation of cAMP-response element binding protein (CREB) and PKA substrates, whereas activation of the MAPK and AKT pathways was not detected (Fig. 3g). Consistent with a PTH1R-dependent mechanism, knockdown of PTH1R in MRC5 cells significantly attenuated the expression of fibrosis-related genes and proteins induced by PTHrP_1-34_ (Fig. 3h and Supplementary Fig. 3h–j). Furthermore, pretreatment with the PKA inhibitor H89 abolished the PTHrP_1-34_-mediated upregulation of fibrosis markers (Fig. 3i and Supplementary Fig. 3k, l), confirming the essential role of the PKA pathway in this process. To determine the effective concentration range of PTHrP_1-34_ in activating PTH1R/PKA signaling, we generated HEK293 cells stably expressing PTH1R (HEK293-PTH1R) and transfected them with a cAMP response element (CRE)-luciferase reporter. Dose–response analysis revealed an EC₅₀ of 18.14 ng/mL for PTHrP-induced luciferase activity (Fig. 3j). These findings collectively demonstrate that PTHrP_1-34_ activates fibroblasts through a PTH1R/PKA/CREB signaling axis, independent of the MAPK and AKT pathways (Fig. 3k). Given that *PTH1R* is highly expressed in smooth muscle cells, we evaluated the role of PTHrP_1-34_ in regulating intracellular calcium levels, ECM synthesis, proliferation, and motility in human primary bronchial smooth muscle cells (HBSMCs). To assess whether PTHrP_1-34_ stimulates calcium influx in HBSMCs, we performed intracellular calcium imaging using Fluo-4 AM. PTHrP_1-34_ did not evoke a measurable Ca²⁺ influx upon acute stimulation, while 50 mM KCl induced a marked increase in intracellular Ca²⁺ as a positive control (Supplementary Fig. 4a). Unlike fibroblasts, treatment of HBSMCs with PTHrP_1-34_ did not result in any detectable changes in the expression of *ACTA2*, *COL1A1*, and *COL3A1* (Supplementary Fig. 4b). The proportion of cells in the S phase of the cell cycle in HBSMCs increased in a supplement concentration-dependent manner, as the supplement contained fetal calf serum (FCS) and some growth factors; however, no detectable changes in cell cycle progression were observed when HBSMCs were cultured in the presence of PTHrP_1-34_ (Supplementary Fig. 4c). Transwell chamber assays revealed no detectable changes in the motility of HBSMCs upon PTHrP_1-34_ treatment (Supplementary Fig. 4d). Unlike HBSMCs cultured with 2% FCS containing growth factors, which exhibited activation of MAPK, PKA, and AKT signaling pathways, cells treated with PTHrP_1-34_ showed no detectable activation of these signaling cascades (Supplementary Fig. 4e). These findings reveal that PTHrP_1-34_ selectively activates fibroblasts via PTH1R/PKA signaling, while HBSMCs remain unresponsive, highlighting a cell type-specific divergence in PTHrP_1-34_-mediated intercellular communication.

### PTHrP_1-34_ exacerbates BLM-induced PF in vivo

**Fig. 4:**
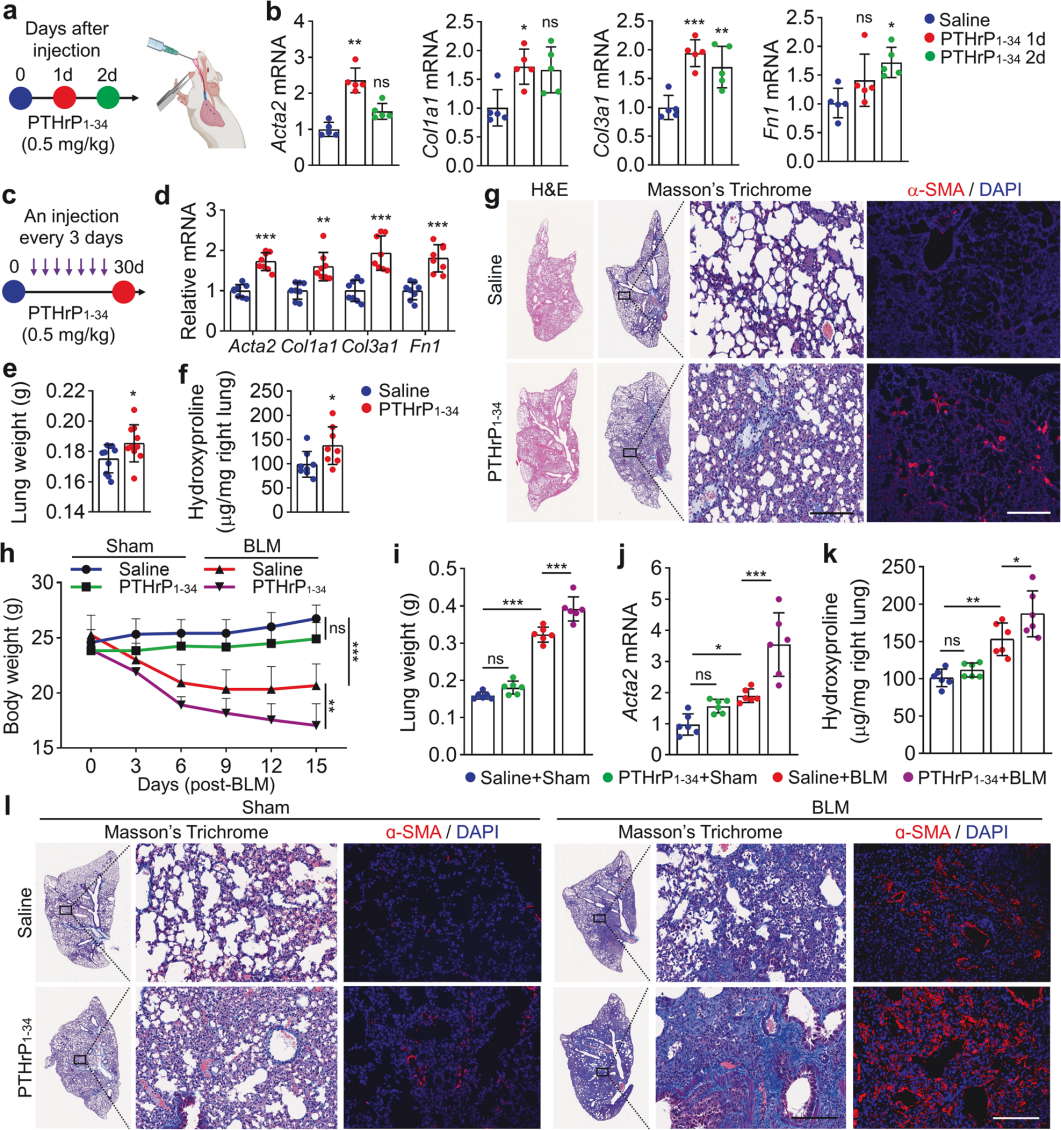
PTHrP_1-34_ activates lung fibroblasts and promotes BLM-induced PF. **a**, **b** Mice were sacrificed and lung tissues were collected 1 and 2 days after IT injection of saline or PTHrP_1-34_ (0.5 mg/kg). (*n* = 5 each group, biological replicates). **a** Schematic of the experimental schedule. The experimental scheme was created with BioRender.com. **b** Expression of fibrosis genes in lung tissues. **c**–**g** Mice were administered saline or PTHrP_1-34_ (0.5 mg/kg) via IT injection every 3 days for 30 days. (*n* = 8 each group, biological replicates). **c** Schematic of the experimental schedule. **d** Expression of fibrosis-related genes. **e** Lung weight of mice at the end of the experiment. **f** Hydroxyproline content in lung tissues. **g** Masson’s trichrome and H&E staining. Scale bar: 100 μm. IF images of ɑ-SMA in lung sections. Scale bar: 200 μm. **h**–**l** After injecting BLM (0.75 mg/kg) into the mice, PTHrP_1-34_ (0.5 mg/kg) was administered by IT injection every 3 days for a total of 12 days, and the animal experiment was terminated 3 days after the final injection (*n* = 6 each group, biological replicates). **h** Changes in the body weight of the mice. **i** Lung weight of mice sacrificed at the end of the experiment. **j** *Acta2* mRNA and **k** hydroxyproline levels in the lungs. **l** Masson’s trichrome and IF images of ɑ-SMA in lung sections. Scale bar: 100 μm. Data are shown as the mean ± SEM. *P* values were analyzed by two-tailed Student’s *t* test in (**d**–**f**), two-way ANOVA (**h**) and one-way ANOVA and Tukey’s test in (**b**, **i**–**k**). *P < 0.05, **P < 0.01, ***P < 0.001. ns not significant

To evaluate the profibrotic effects of PTHrP_1-34_ under physiological conditions, we assessed the expression of fibrosis-related genes in the lungs of PTHrP_1-34_-treated mice. Intratracheal (IT) administration of PTHrP_1-34_ for 1 or 2 days significantly upregulated fibrosis genes in mouse lungs (Fig. 4a, b). Consistent with short-term exposure studies, repeated administration of PTHrP_1-34_ every 3 days over a period of 1 month also elevated the expression of fibrosis genes (Fig. 4c, d). Long-term administration of PTHrP_1-34_ led to a modest increase in lung weight by approximately 5.7% and a substantial elevation in hydroxyproline content by approximately 35% (Fig. 4e, f). Following chronic exposure to PTHrP_1-34_, elevated levels of α-SMA protein were observed in lung tissues, whereas Masson’s trichrome staining did not reveal a clear accumulation of collagen, suggesting that although fibrotic markers were upregulated, overt fibrosis was not prominent (Fig. 4g). These findings suggest that PTHrP_1-34_ partially contributes to collagen production and fibrotic remodeling through aberrant activation of fibroblasts; however, whether PTHrP_1-34_ alone is sufficient to initiate and drive the onset and progression of PF remains to be explored. To further assess the profibrotic potential of PTHrP_1-34_, we employed a low-dose BLM-induced PF model (0.75 mg/kg) and evaluated the effects of repeated PTHrP_1-34_ administration. Compared with the mice in the PTHrP_1-34_-only and BLM-only groups, mice co-treated with BLM and PTHrP_1-34_ exhibited a marked reduction in body weight and an increase in lung weight (Fig. 4h, i). While PTHrP_1-34_ alone modestly elevated the expression of fibrosis-related genes, co-treatment with BLM resulted in a dramatic upregulation of these genes and hydroxyproline content (Fig. 4j, k and Supplementary Fig. 5a). Histological analysis revealed that co-administration of BLM and PTHrP_1-34_ led to increased cell density, severe collagen deposition, and a marked elevation in α-SMA expression in the lungs (Fig. 4l and Supplementary Fig. 5b). These findings demonstrate that PTHrP_1-34_ markedly exacerbates fibrotic outcomes in the context of lung injury, acting as a potent driver of tissue remodeling. Given that the pathogenesis of PF is initiated by alveolar epithelial cell injury followed by inflammation and the secretion of profibrotic mediators that aberrantly activate lung fibroblasts, we investigated the effects of PTHrP_1-34_ on epithelial cell injury in both alveolar and bronchial compartments. Chronic exposure of mice to PTHrP_1-34_ did not result in macrophage infiltration in lung tissue, suggesting that PTHrP_1-34_ does not exert proinflammatory effects (Supplementary Fig. 5c). Consistently, the expression of key inflammatory and profibrotic cytokines, including *IL-6*, *TGFB1*, and *IL-11*, remained unchanged upon PTHrP_1-34_ treatment in A549 cells, human primary alveolar epithelial cells (AECs), and human primary bronchial epithelial cells (HBEpCs) (Supplementary Fig. 5d). To assess the cellular response of epithelial cells to PTHrP_1-34_, we cultured AECs, mouse AT2 (mAT2), and HBEpC cells with PTHrP_1-34_ for 24 h. No significant changes in cell viability were observed (Supplementary Fig. 5e). In AECs exposed to hydrogen peroxide (H_2_O_2_), cleaved-caspase-3 levels were markedly increased, whereas PTHrP_1-34_ treatment did not alter cleaved-caspase-3 expression (Supplementary Fig. 5f). Additionally, PTHrP_1-34_ did not affect the MAPK, PKA, or AKT signaling pathways in either AECs or HBEpC cells (Supplementary Fig. 5g). PTHrP_1-34_ does not induce cytotoxicity or activate canonical signaling pathways in lung alveolar and bronchial epithelial cells, indicating cell type-specific responsiveness. In addition, PTHrP_1-34_ induced the expression of α-SMA and COL1A1 even in the presence of the TGFβ1/Smad pathway inhibitor LY2109761 (Supplementary Fig. 5h). PTHrP_1-34_ did not stimulate TGFβ1 production or Smad3 phosphorylation (Supplementary Fig. 5i–k), indicating that its profibrotic effects are independent of the canonical TGFβ1/Smad signaling axis. Collectively, these observations indicate that PTHrP_1-34_ functions as a potent enhancer of PF by promoting fibroblast activation and tissue remodeling in injured lungs, while exerting minimal direct effects on epithelial cells, highlighting its cell type-specific profibrotic activity.

### Bronchial epithelial cell-derived PTHrP_1-34_ drives fibroblast activation in BLM-induced lung injury

**Fig. 5:**
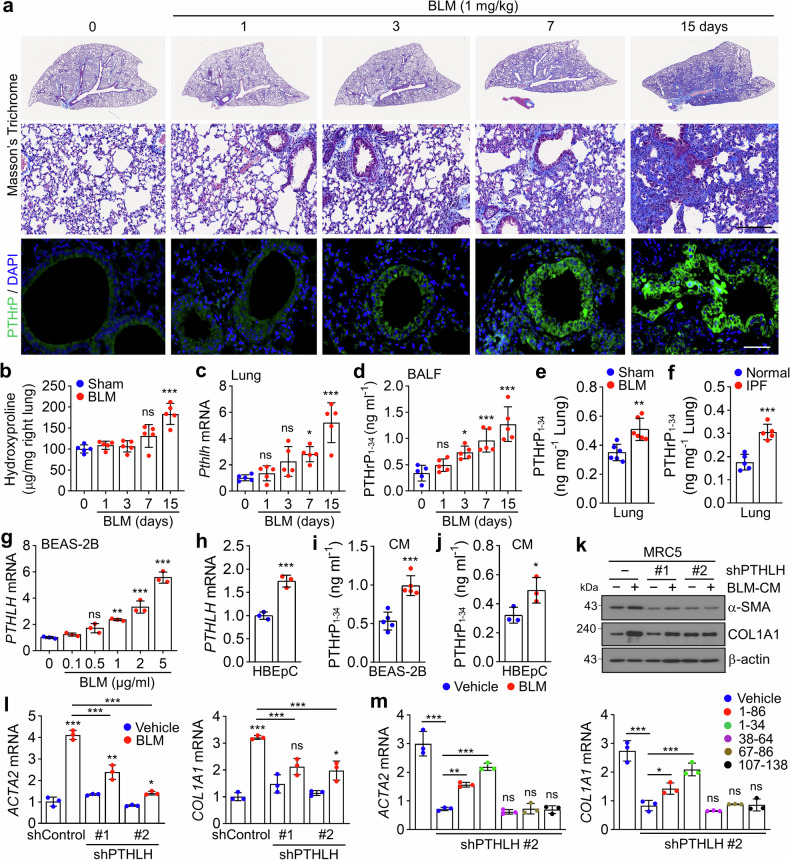
BLM induces PTHrP_1-34_ secretion from bronchial epithelial cells. **a**–**f** Timeline of BLM (1 mg/kg) intratracheal administration and sacrifice at 0, 1, 3, 7, and 15 days post-injection (*n* = 5 each group, biological replicates). **a** Masson’s trichrome staining and IF images of PTHrP (green) expression in mouse lung tissue. Scale bar: 100 μm. **b** Hydroxyproline content in the lungs. **c** *Pthlh* mRNA expression levels in lung lysates. **d** Quantification of PTHrP_1-34_ in BALF. **e** Quantification of PTHrP_1-34_ in lung lysates **f** Quantification of PTHrP_1-34_ in human IPF patients (*n* = 5, biological replicates). **g** *PTHLH* mRNA levels in BEAS-2B cells treated with the indicated concentrations of BLM for 48 h. **h** *PTHLH* mRNA levels following BLM stimulation in HBEpC for 48 h. **i** Quantification of PTHrP_1-34_ in CM derived from BLM-treated BEAS-2B cells. **j** Quantification of PTHrP_1-34_ in CM derived from BLM-treated HBEpCs. **k** Fibrosis-related proteins in MRC5 cells stimulated with CM from BLM-treated, shPTHLH-infected BEAS-2B cells. **l** Fibrosis-related genes in MRC5 cells stimulated with CM from BLM-treated, shPTHLH-infected BEAS-2B cells. **m** MRC5 cells were treated with CM derived from BLM-treated, shPTHLH lentivirus-infected BEAS-2B cells, followed by treatment with PTHrP domain peptides (aa 1-86, 1-34, 38–64, 67–86, and 107–138). Data are shown as the mean ± SEM. *P* values were determined by two-tailed Student’s *t* test in (**e**, **f**, **h**–**j**) and one-way ANOVA and Tukey’s test in (**b**–**d**, **g**, **l**, **m**). *P < 0.05, **P < 0.01, ***P < 0.001. ns not significant

To delineate the pathophysiological role of PTHrP_1-34_ in lung injury-induced PF, we investigated its temporal expression during the inflammatory and fibrotic phases. Following BLM administration (1 mg/kg), PTHrP expression in bronchial epithelial cells showed a modest increase by day 3, followed by a marked elevation on days 7 and 15 (Fig. 5a). This upregulation coincided with progressive fibrotic changes, including body weight loss, increased lung weight, pulmonary edema, and old hemorrhagic lesions (Supplementary Fig. 6a, b). Lung lysates revealed elevated levels of fibrotic markers and full-length PTHrP protein (Supplementary Fig. 6c, d). Notably, hydroxyproline content and *Pthlh* mRNA expression began to rise by day 7 and peaked on day 15 (Fig. 5b, c), whereas *Tnf-α* mRNA, a proinflammatory cytokine, was highest on days 3 and 7 (Supplementary Fig. 6e). These findings suggest that PTHrP upregulation follows the initial inflammatory response and may contribute to the transition toward fibrosis. Consistent with this, PTHrP_1-34_ levels in BALF increased approximately twofold on day 7 and further rose to threefold by day 15 compared to those in controls (Fig. 5d). In lung homogenates, PTHrP_1-34_ levels were elevated by 43% on day 15 (Fig. 5e), while no significant changes were observed in the plasma (Supplementary Fig. 6f), indicating that PTHrP_1-34_ is locally and dynamically upregulated in the lung following BLM-induced injury, with peak expression during the fibrotic phase. Supporting the clinical relevance of these findings, analysis of lung homogenates from patients with IPF revealed a 57.5% increase in PTHrP_1-34_ levels compared to those from normal lung tissues (Fig. 5f), suggesting that PTHrP_1-34_ upregulation is a conserved feature of fibrotic lung remodeling. These results suggest that epithelial-derived PTHrP_1-34_ may play a pivotal role in the transition from inflammation to fibrosis, independent of systemic circulation. Hypoxia, a hallmark of fibrotic lung environments, promotes disease progression and has been shown to upregulate PTHrP expression.^27^ Thus, we further examined whether hypoxia itself could induce PTHrP expression. BEAS-2B and HBEpC cells were cultured under hypoxic conditions, which led to a significant increase in *PTHLH* mRNA expression and elevated levels of PTHrP_1-34_ in the conditioned medium (CM) (Supplementary Fig. 6g, h). These findings support the notion that hypoxia may involve epithelial PTHrP production. To provide direct evidence of PTHrP_1-34_ secretion from bronchial epithelial cells and its potential role in epithelial–fibroblast communication, we analyzed BLM-induced changes in PTHrP_1-34_ expression and release using BEAS-2B and HBEpC cells. In BEAS-2B cells, *PTHLH* mRNA expression increased in a dose-dependent manner upon BLM treatment (Fig. 5g), and a similar increase was observed in HBEpCs (Fig. 5h). Consistent with these transcriptional changes, PTHrP_1-34_ levels were significantly elevated in the CM from both BLM-treated BEAS-2B and HBEpC cells (Fig. 5i and j), providing direct evidence of PTHrP_1-34_ secretion in response to epithelial injury and supporting its role in intercellular signaling during fibrotic remodeling. CM from BLM-treated BEAS-2B cells (BLM-CM) induced the expression of fibrotic markers in MRC5 fibroblasts at both the mRNA and protein levels. In contrast, BLM-CM derived from *PTHLH*-knockdown BEAS-2B cells elicited a markedly attenuated induction of these markers (Fig. 5k, l). Notably, *ACTA2* expression was reduced by approximately 50% in fibroblasts treated with BLM-CM from *PTHLH*-silenced cells compared to that in control BLM-CM, suggesting that PTHrP_1-34_ is a key profibrotic factor among the multiple mediators present in BLM-CM. To directly assess the contribution of PTHrP_1-34_ to the profibrotic activity of BLM-CM, we supplemented BLM-CM from *PTHLH*-silenced BEAS-2B cells with various PTHrP peptides. Treatment of MRC5 fibroblasts with these supplemented media revealed that both PTHrP_1-34_ and the longer PTHrP_1-86_ fragment restored the expression of fibrotic genes (Fig. 5m), confirming the functional relevance of epithelial-derived PTHrP_1-34_ in fibroblast activation. Collectively, these observations indicate that epithelial-derived PTHrP_1-34_ is a key mediator of fibroblast activation in response to BLM-induced lung injury.

### PTHrP neutralizing antibody (α-PTHrP) prevents and reverses BLM-induced PF in mice

**Fig. 6:**
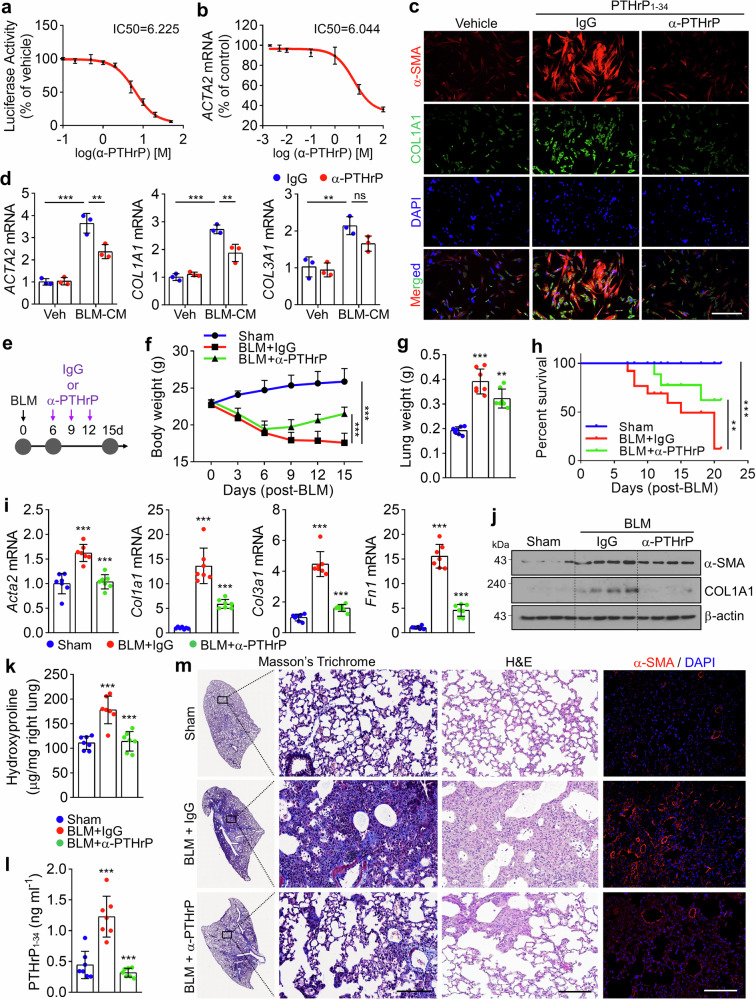
PTHrP_1-34_ neutralizing antibody (α-PTHrP) attenuates BLM-induced PF. **a** The IC_50_ value of the α-PTHrP was determined based on CRE-luciferase activity in PTHrP_1-34_-treated HEK293-PTH1R cells. **b** The IC_50_ value of the α-PTHrP was determined based on *ACTA2* mRNA expression in PTHrP_1-34_-treated MRC5 cells. **c** MRC5 cells were pretreated with α-PTHrP (5 μg/mL), followed by stimulation with PTHrP_1-34_. Representative IF images of α-SMA and COL1A1. Scale bar: 200 μm **d** CM from BLM (5 μg/mL, 48 h)-treated BEAS-2B cells was mixed with α-PTHrP (5 μg/mL) and applied to MRC5 cells for analysis of fibrosis genes. **e**–**g**, **i**–**m** α-PTHrP (1 mg/kg) was administered by IT injection every 3 days for 12 days, starting on day 6 after BLM (1 mg/kg) challenge, and the animal experiment was terminated on day 15 (*n* = 7 each group, biological replicates). **e** Scheme for the experimental schedule. **f** Changes in the body weight of the mice. **g** Lung weight of mice measured after sacrifice at the end of the experiment. **h** Survival rate analysis in mice. α-PTHrP was administered by IT injection every 3 days for 18 days, starting on day 6 after BLM (2 mg/kg) challenge; the animal experiment was stopped on day 21 (*n* = 10 each group, biological replicates). **i** Expression of fibrosis-related genes and **j** expression of proteins in the lungs of mice. **k** Hydroxyproline content in the lungs. **l** Quantification of PTHrP_1-34_ in the BALF of mice. **m** Masson’s trichrome, H&E staining, and IF images of α-SMA in lung sections. Scale bar: 100 μm. All data are shown as the mean ± SEM. *P* values were analyzed by two-way ANOVA (**f**, **h**) and one-way ANOVA and Tukey’s test in (**d**, **g**, **i**, **k**, **l**). **P < 0.01, ***P < 0.001. ns not significant

To evaluate the neutralizing capacity and effective dose of α-PTHrP, we first assessed CRE-luciferase activity in HEK293-PTH1R cells stimulated with PTHrP_1-34_. The antibody dose-dependently inhibited PTHrP_1-34_-induced CRE-Luc activation, with an IC₅₀ value of 6.225 μg/mL (Fig. 6a). Consistent with this result, treatment of MRC5 cells with 6.044 μg/mL α-PTHrP reduced PTHrP_1-34_-induced *ACTA2* expression by approximately 50% (Fig. 6b). Moreover, fibrosis markers upregulated by PTHrP_1-34_ were dramatically suppressed at both the mRNA and protein levels by 5 μg/mL α-PTHrP in MRC5 and IPF patient-derived lung fibroblasts (Fig. 6c and Supplementary Fig. 7a–c). Importantly, α-PTHrP at 5 μg/mL also significantly attenuated the BLM-CM-induced expression of fibrosis genes and proteins (Fig. 6d and Supplementary Fig. 7d), further supporting its therapeutic potential in fibrotic contexts. In this study, we identified that PTHrP is markedly upregulated between days 3 and 7 following BLM administration. This temporal pattern aligns with established preclinical guidelines for BLM-induced PF models, which designate day 6 post-BLM injection as a representative time point for the late phase of ALI (days 0–7) and the early stage of fibroproliferation (days 3–14).^28^ Based on these findings, we selected day 6 as the intervention point to evaluate the antifibrotic efficacy of α-PTHrP in vivo, aiming to target the early fibrotic signaling cascade initiated by BLM-induced PTHrP upregulation. Six days after BLM administration, α-PTHrP (1 mg/kg) was administered by IT instillation every 3 days for a total of 3 times, and the mice were monitored for a total of 15 days (Fig. 6e). Severe body weight loss and increased lung weight in BLM-induced PF mice were significantly reversed by α-PTHrP administration (Fig. 6f, g). An increased survival rate of BLM-induced PF mice was observed after administration of α-PTHrP (Fig. 6h). Lower expression of fibrosis genes and proteins was observed in the lungs of BLM-induced PF mice treated with α-PTHrP compared with those in the IgG-treated mice (Fig. 6i, j and Supplementary Fig. 7e). Increased hydroxyproline levels, indicating collagen accumulation in BLM-induced PF lungs, were significantly reversed by α-PTHrP administration (Fig. 6k). BALF collected from the α-PTHrP-administered mice contained lower levels of PTHrP_1-34_ compared with that collected from mice treated with IgG in the BLM-induced PF model (Fig. 6l), indicating that α-PTHrP can trigger PTHrP_1-34_ elimination within the bronchial lumen and in the fibrotic region. Histological evaluation, including H&E, Masson’s trichrome, and ɑ-SMA staining of lung slices, showed that α-PTHrP administration effectively reduced and recovered fibrotic remodeling of the lung parenchyma, collagen deposition (blue staining), and myofibroblasts compared with IgG administration in the lungs of the BLM-induced PF model (Fig. 6m). To assess the clinical relevance of early therapeutic intervention with α-PTHrP during the acute inflammatory phase of lung injury, α-PTHrP treatment was initiated 3 days after BLM injection, a time point at which PTHrP expression begins to increase. This timing was chosen to target the early fibrotic signaling cascade triggered by BLM-induced PTHrP upregulation. In this context, α-PTHrP treatment effectively reversed BLM-induced body weight loss and the increased expression of fibrosis genes (Supplementary Fig. 7f, g). To determine the optimal therapeutic dose of α-PTHrP, compare its efficacy with the approved antifibrotic drug nintedanib, and assess long-term treatment outcomes, we administered low-dose α-PTHrP (0.5 mg/kg and 0.1 mg/kg) via IT instillation every 3 days starting on day 6 post-BLM injection. In parallel, nintedanib (30 mg/kg) was administered orally once daily (Supplementary Fig. 7i). We compared body weight recovery as a surrogate marker of disease severity. Nine days after treatment initiation, mice in the BLM-only group exhibited an average body weight of 17–18 g. In contrast, mice treated with 0.1 mg/kg α-PTHrP showed a modest recovery to 19.5 g (11.43% increase), while those receiving 0.5 mg/kg and 1 mg/kg demonstrated more substantial recovery to 21.5 g (22.86% increase) (Fig. 6f and Supplementary Fig. 7h). The similar efficacy observed for the 0.5 mg/kg and 1 mg/kg groups suggests a plateau effect, indicating that 0.5 mg/kg may represent the minimum effective dose. Notably, 0.5 mg/kg α-PTHrP showed comparable body weight recovery to nintedanib, and 1 mg/kg α-PTHrP outperformed nintedanib. No further enhancement in body weight recovery was observed on day 15 compared to that on day 9 (Supplementary Fig. 7 h). Analysis of hydroxyproline content and fibrosis gene expression revealed similar reductions in mice treated with nintedanib and in those receiving low-dose α-PTHrP (0.5 or 0.1 mg/kg) (Supplementary Fig. 7j, k). Histological examination further confirmed that collagen accumulation was comparably attenuated in both treatment groups, supporting the antifibrotic efficacy of α-PTHrP (Supplementary Fig. 7l). These findings collectively demonstrate that α-PTHrP effectively neutralizes PTHrP_1-34_ signaling and alleviates BLM-induced PF, showing comparable or superior therapeutic efficacy to nintedanib even at low doses.

### PTHrP_7-34_ is a potent antagonist of PTHrP_1-34_/PTH1R signaling in BLM-induced PF in mice

**Fig. 7:**
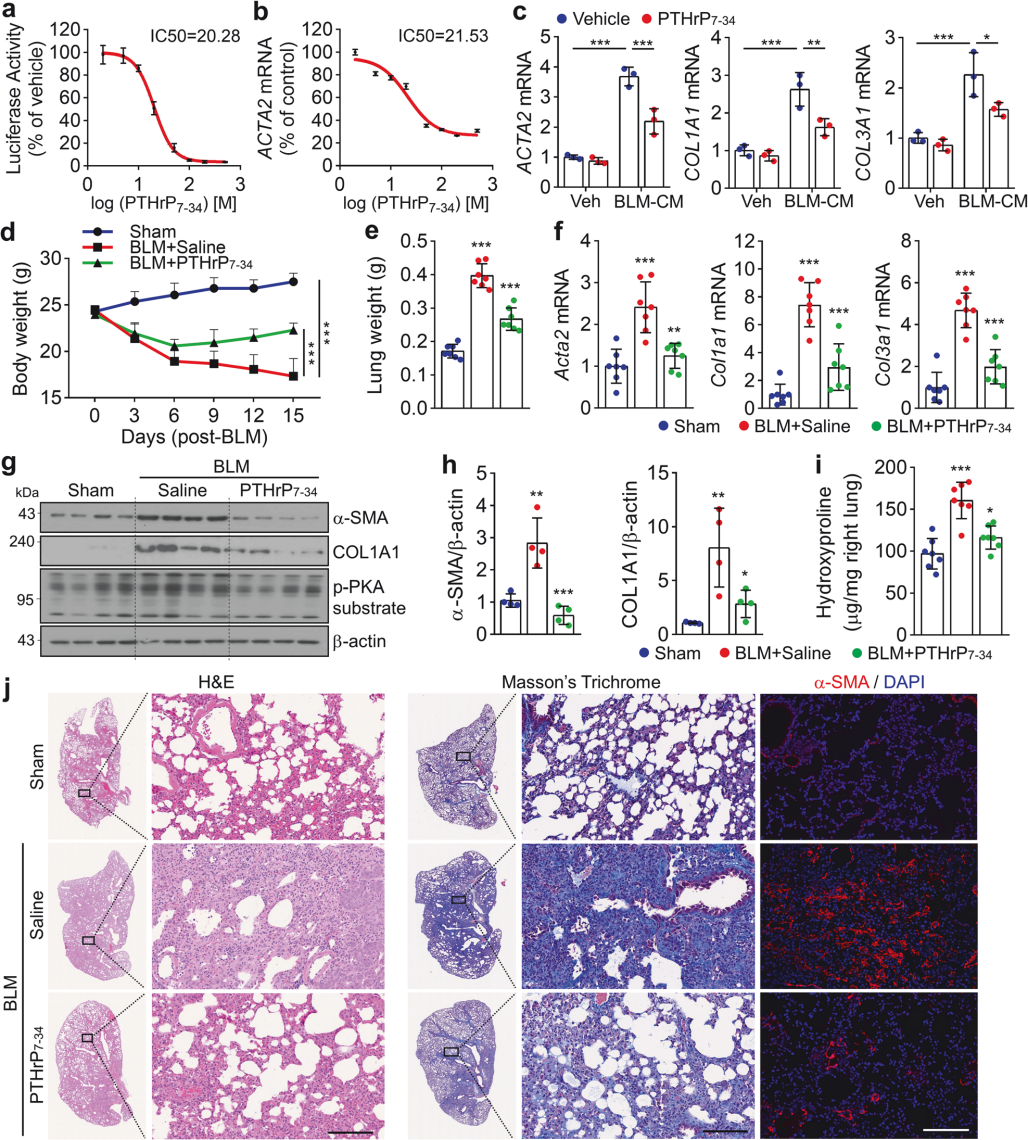
Inhibition of the PTHrP_1-34_/PTH1R axis attenuates BLM-induced PF. **a** The IC_50_ value of the PTHrP_7-34_ was determined based on CRE-luciferase activity in PTHrP_1-34_-treated HEK293-PTH1R cells. **b** IC_50_ value of the PTHrP_7-34_ was determined based on *ACTA2* mRNA expression in PTHrP_1-34_-treated MRC5 cells. **c** CM from BLM (5 μg/mL, 48 h)-treated BEAS-2B cells was mixed with PTHrP_7-34_ (100 ng/mL) and applied to MRC5 cells for analysis of fibrosis genes. **d**–**j** BLM (1 mg/kg) was administered once to mice, and 6 days later, PTHrP_7-34_ (0.5 mg/kg) was administered by IT injection every 3 days for a total of 12 days. The animal experiment was terminated 3 days after the final injection (*n* = 7 each group, biological replicates). **d** Changes in the body weight of the mice. **e** Lung weight of mice sacrificed at the end of the experiment. **f** Fibrosis-related genes in the lungs of mice. **g** Fibrosis-related proteins in the lungs of mice. **h** Quantification of α-SMA and COL1A1 band intensity in lung tissue. **i** Hydroxyproline content in the lungs. **j** Masson’s trichrome, H&E staining, and IF images of ɑ-SMA in lung sections. Scale bar: 100 μm. All data are shown as the mean ± SEM. *P* values were analyzed by two-way ANOVA (**d**) and one-way ANOVA and Tukey’s test (**c**, **e**, **f**, **h**, **i**). **P < 0.01, ***P < 0.001

Next, we investigated the antagonistic efficacy of PTHrP_7-34_, a competitive peptide inhibitor designed to disrupt the bipartite interaction between PTHrP_1-34_ and PTH1R, wherein the N-terminal region of PTHrP engages the TMD to activate Gs signaling, and the C-terminal region binds to the ECD to stabilize receptor affinity and specificity.^29,30^ In HEK293-PTH1R cells, PTHrP_7-34_ markedly suppressed CRE-luciferase activity triggered by PTHrP_1-34_ stimulation, with half-maximal inhibition observed at 20.28 ng/mL (Fig. 7a). A similar dose-dependent attenuation was noted in MRC5 cells, where *ACTA2* mRNA levels were reduced with an IC₅₀ of 21.53 ng/mL (Fig. 7b). Consistent with this result, PTHrP_7-34_ inhibited PKA substrate phosphorylation and substantially downregulated fibrosis-related transcripts and proteins induced by PTHrP_1-34_ (Supplementary Fig. 8a, b). Furthermore, when exposed to BLM-CM, cells treated with PTHrP_7-34_ exhibited significantly diminished expression of fibrotic markers at both the transcriptional and translational levels (Fig. 7c and Supplementary Fig. 8c), indicating that antagonistic peptides to PTHrP_1-34_/PTH1R have potential as therapeutic modulators in fibrotic signaling. To evaluate the antifibrotic efficacy of PTHrP_7-34_ in vivo, we conducted a preclinical study using the BLM-induced PF model, following the same experimental schedule as that applied for α-PTHrP (Fig. 6e). Administration of PTHrP_7-34_ (0.5 mg/kg) significantly reversed severe body weight loss and reduced the elevated lung weight observed in BLM-treated mice (Fig. 7d, e). Quantitative analysis revealed markedly lower expression of fibrosis genes and proteins in lung tissues from PTHrP_7-34_-treated mice than in saline controls (Fig. 7f, g and Supplementary Fig. 8d). Notably, BLM-induced phosphorylation of PKA substrates was substantially attenuated by PTHrP_7-34_ treatment (Fig. 7g, h). Hydroxyproline content was also significantly reduced in the lungs of PTHrP_7-34_-treated mice (Fig. 7i). Histological assessments demonstrated that PTHrP_7-34_ effectively mitigated fibrotic remodeling, collagen deposition, and myofibroblast expansion in the lung parenchyma (Fig. 7j). To determine the optimal therapeutic dose and evaluate the comparative and sustained antifibrotic efficacy of PTHrP_7-34_, we administered low doses of the peptide (0.05 and 0.2 mg/kg, intratracheally, every 3 days) and nintedanib (30 mg/kg, orally, daily), starting on day 6 post-BLM instillation. By day 9 of treatment, PTHrP_7-34_ exhibited a dose-dependent effect on body weight recovery, with increases of 2.78%, 8.33%, and 22.22% at 0.05, 0.2, and 0.5 mg/kg, respectively, compared to that in the BLM-only group (Supplementary Fig. 8e, f). Nintedanib treatment resulted in a 17% recovery, which was surpassed by that in the 0.5 mg/kg PTHrP_7-34_ group, while the 0.2 mg/kg group showed a relatively modest effect. Continued monitoring until day 15 revealed a slight additional improvement in body weight across all treatment groups (Supplementary Fig. 8e). Molecular analysis of lung tissues demonstrated comparable reductions in hydroxyproline content and fibrosis gene expression between mice treated with nintedanib and those receiving 0.2 mg/kg PTHrP_7-34_ (Supplementary Fig. 8g, h). Consistently, Masson’s trichrome staining confirmed that collagen deposition was similarly attenuated in both groups (Supplementary Fig. 8i). Given the known involvement of PTHrP_1-34_/PTH1R signaling in calcium homeostasis,^15^ we assessed whether localized IT administration of PTHrP_7-34_ or α-PTHrP antibody alters systemic calcium levels. Serum calcium measurements revealed no significant changes in mice treated with either PTHrP_7-34_ or α-PTHrP, comparable to those observed in the nintedanib-treated and saline control groups, indicating that local airway delivery of these agents does not perturb systemic calcium metabolism (Supplementary Fig. 8j). These findings collectively demonstrate that PTHrP_7-34_, as a competitive antagonist of PTHrP_1-34_/PTH1R signaling, exerts potent antifibrotic effects both in vitro and in vivo, with therapeutic efficacy comparable to or exceeding that of nintedanib, depending on the dose.

### Genetic suppression of *Pthlh* attenuates BLM-induced PF in mice

**Fig. 8:**
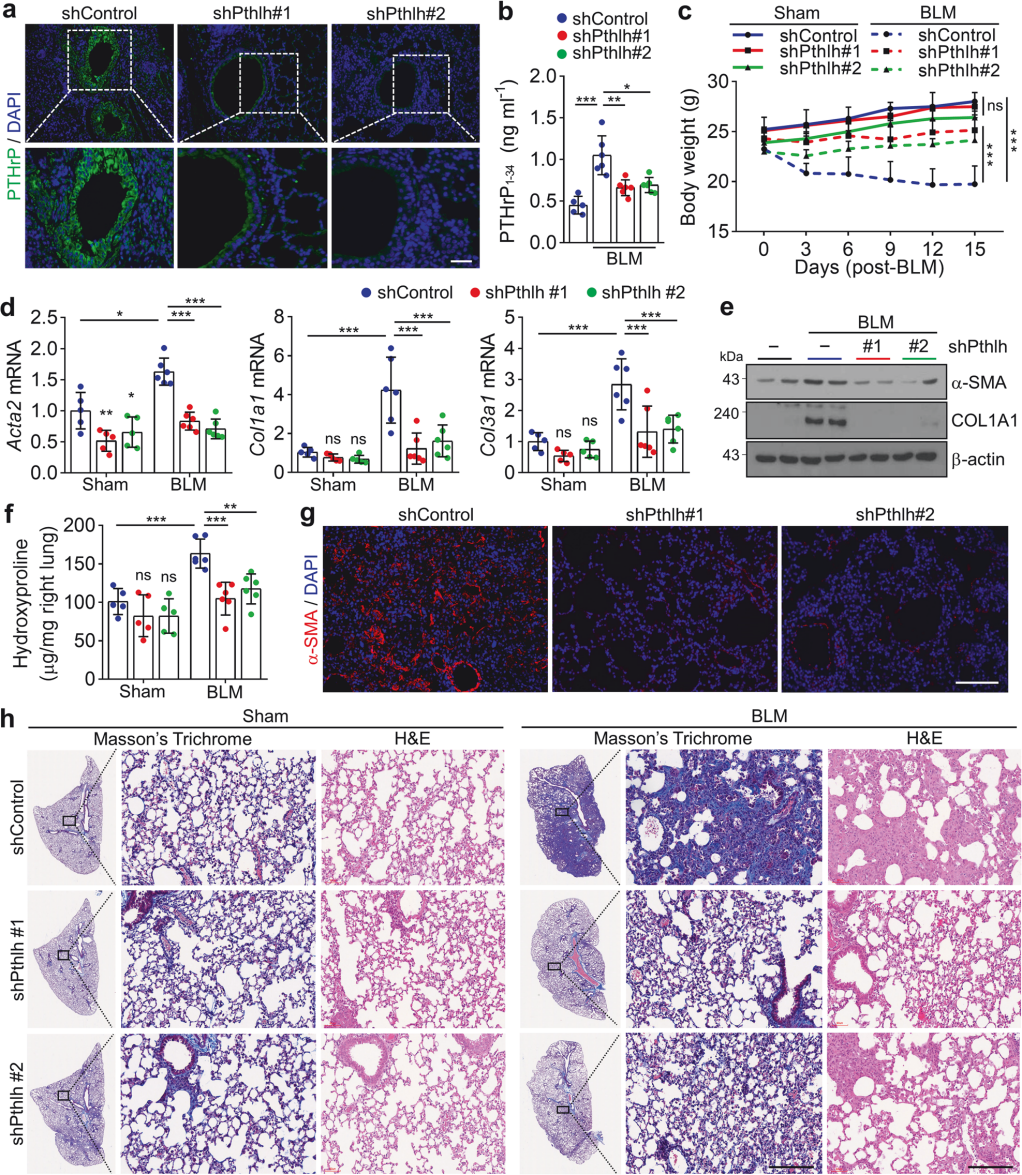
Genetic suppression of *Pthlh* attenuates BLM-induced PF. **a**–**h** Mice were administered lentivirus expressing shRNA targeting control (shControl) or *Pthlh* (shPthlh#1 and shPthlh#2) by IT injection twice, every 3 days, starting 6 days before challenge with BLM (1 mg/kg). After BLM administration, the mice were monitored for 15 days. (Sham group, *n* = 5; BLM-shControl group, *n* = 6; BLM-shPthlh#1 and #2 group, *n* = 7, biological replicates). **a** Representative IF images of PTHrP (green) in the lungs of mice. Scale bar: 100 μm. **b** Quantification of PTHrP_1-34_ in the BALF of mice. **c** Changes in body weight of mice **d** Expression of fibrosis-related genes and **e** protein expression in the lungs of mice. **f** Hydroxyproline content in the lungs. **g** Representative IF images of ɑ-SMA (red) in the lungs of mice. Scale bar: 100 μm. **h** Masson’s trichrome and H&E staining of lung sections. Scale bar: 100 μm. All data are shown as the mean ± SEM. *P* values were analyzed by two-way ANOVA (**c**) and one-way ANOVA and Tukey’s test (**b**, **d**, **f**). *P < 0.05, **P < 0.01, ***P < 0.001. ns not significant

To determine whether genetic silencing of *Pthlh* could attenuate PF progression, we employed a lentivirus-mediated RNA interference (RNAi) strategy using short hairpin RNA. Lentiviruses expressing either shControl or shPthlh were administered intratracheally to mice twice at 3-day intervals. Two days after the second administration, a low dose of BLM (1 mg/kg) was administered, and the mice were monitored for 15 days. Immunostaining revealed reduced PTHrP expression in the bronchial epithelium of shPthlh-treated mice compared to that in the BLM-induced PF lungs (Fig. 8a). Furthermore, BALF analysis showed an approximately 20% reduction in PTHrP_1-34_ levels in the shPthlh group compared to the shControl group, confirming effective knockdown of *Pthlh* expression (Fig. 8b). Next, we evaluated the impact of *Pthlh* silencing on body weight loss associated with BLM-induced PF. In the Sham group, shPthlh administration did not affect body weight, indicating that *Pthlh* knockdown alone does not elicit systemic effects. However, following BLM treatment, mice in the shControl group exhibited a 32% reduction in body weight by day 15, whereas those in the shPthlh-treated BLM group showed only a 10% decrease, suggesting that *Pthlh* silencing significantly mitigated BLM-induced weight loss (Fig. 8c). Consistent with these findings, the upregulation of fibrosis genes and proteins in BLM-induced PF lungs was significantly reversed by *Pthlh* knockdown (Fig. 8d and e). Similarly, the elevated hydroxyproline content was markedly reduced in the lungs of shPthlh-treated mice (Fig. 8f). Immunohistochemical analysis further revealed a dramatic decrease in α-SMA expression in the lungs of shPthlh-treated mice (Fig. 8g). Histological examination using H&E and Masson’s trichrome staining confirmed that collagen deposition was significantly reduced in the lungs of shPthlh-treated mice compared with shControl group mice (Fig. 8h). Together, these results demonstrate that *Pthlh* knockdown effectively suppresses key pathological features of BLM-induced PF, highlighting its potential as a therapeutic target for IPF.

## Discussion

This study demonstrates that PTHrP_1-34_ is elevated in lung tissues from patients with IPF and in a BLM-induced PF mouse model. Intratracheal administration of PTHrP_1-34_ alone modestly increased fibrosis-related gene expression, collagen deposition, and fibrotic lesions, even in the absence of BLM. In vitro, human and mouse lung fibroblasts treated with PTHrP_1-34_ exhibited dose- and time-dependent activation and differentiation into myofibroblasts. Notably, long-term exposure to PTHrP_1-34_ did not induce macrophage infiltration, suggesting that fibrosis may proceed independently of classical inflammatory pathways. Experiments using primary human AECs, HBEpC, and mouse AT2 cells confirmed that PTHrP_1-34_ does not activate MAPK, PKA, or AKT signaling, nor does it affect cell survival, apoptosis, or the production of profibrotic cytokines such as IL-6, TGFβ1, and IL-11. Single-cell RNA sequencing revealed that PTH1R, the receptor for PTHrP_1-34_, is expressed in smooth muscle cells and fibroblasts but not in AT1 or AT2 epithelial cells. These findings suggest that PTHrP_1-34_ promotes fibrogenesis primarily through the direct activation of fibroblasts, rather than via epithelial injury or inflammation.

PTHrP has also been implicated in fibrosis of other organs, including the kidney and liver. In folic acid-induced kidney injury, PTHrP contributes to renal fibrosis, and in murine models of liver fibrosis induced by carbon tetrachloride or a methionine-deficient and choline-deficient diet, PTHrP and its receptor PTH1R are pathologically involved.^31,32^ Treatment with PTHrP_1-36_ has been shown to increase TGFβ1 secretion from tubuloepithelial and hepatic stellate cells.^33,34^ While the downstream mechanisms of fibroblast activation via PTH1R signaling are well characterized, the specific cell types responsible for secreting PTH1R ligands remain poorly defined. In our reanalysis of scRNA-seq data, *PTHLH* was predominantly expressed in bronchial epithelial cells, with much lower expression in liver and kidney tissues.^35^ These findings suggest that PTHrP-mediated fibrogenesis may be a more prominent pathological event in the respiratory airways than in other organs.

The bronchial epithelium consists of diverse epithelial cell types, including ciliated, goblet, club, and basal cells, which collectively function as a physical barrier and immune defense against inhaled particles and pathogens.^36,37^ Respiratory basal cells, a stem-like population capable of differentiating into various epithelial subtypes, are essential for maintaining epithelial homeostasis.^38^ Our data show that PTHrP is strongly expressed in bronchial epithelial cells, particularly in respiratory basal, club, and basaloid cells. Given its established role as a paracrine regulator in multiple organ systems, including bone, lung, and mammary gland, PTHrP may similarly regulate the microenvironment of the lower airways through paracrine signaling.^39–41^ This hypothesis is supported by our observation that PTHrP_1-34_ administration alters fibrotic responses even in the absence of additional injury, suggesting that PTHrP functions as a central paracrine effector in bronchial–alveolar signaling.

Recent advances in lower respiratory tract research have highlighted the importance of epithelial crosstalk between bronchial and alveolar compartments in lung development and chronic lung disease pathogenesis.^42^ Extracellular vesicles derived from bronchial epithelial cells have been shown to modulate fibrotic responses and chronic inflammation in IPF and COPD, underscoring the active role of the bronchial epithelium in distal lung remodeling.^43,44^ Despite growing evidence of bronchial–alveolar interactions, the specific soluble mediators—such as cytokines, hormones and growth factors—secreted by the bronchial epithelium that regulate alveolar repair remain poorly characterized. Our findings demonstrate that PTHrP upregulation in IPF is driven by epithelial stress responses, as evidenced by increased *PTHLH* mRNA and PTHrP_1-34_ secretion in BEAS-2B and HBEpC cells under hypoxia or BLM stimulation. Reanalysis of scRNA-seq data from IPF lung tissue revealed elevated *PTHLH* expression in airway epithelial cells, basal cells, and aberrant basaloid cells.^20,45,46^ In BLM-induced fibrotic lungs, PTHrP was co-expressed with p63 and KRT17, which are markers of basaloid cells, suggesting a link between PTHrP expression and basaloid cell expansion. These results support a model in which epithelial-derived PTHrP contributes to fibroblast activation and extracellular matrix production, thereby promoting fibrosis progression. Understanding the molecular drivers of epithelial crosstalk may offer new therapeutic avenues for IPF and other interstitial lung diseases.

Our preclinical investigation using the BLM-induced PF model identified multiple therapeutic strategies targeting PTHrP to mitigate disease progression. Among these, α-PTHrP demonstrated the most pronounced efficacy, significantly alleviating clinical symptoms such as body weight loss, collagen deposition, and widespread fibrotic lesions in the lungs, while markedly improving survival rates. These findings are consistent with previous reports of the therapeutic potential of α-PTHrP in lung cancer-associated cachexia and metastasis in breast and pancreatic cancers.^13,47,48^ In parallel, knockdown of the *Pthlh* gene, which encodes PTHrP, effectively blocked the progression of BLM-induced PF, further supporting the pathological role of epithelial-derived PTHrP in fibrotic remodeling. Additionally, PTHrP_7-34_, a selective peptide antagonist that disrupts the bipartite interaction between PTHrP_1-34_ and PTH1R, protected mice from BLM-induced PF by inhibiting the PTH1R/PKA signaling axis. PTHrP_1-34_-induced activation of lung fibroblasts, characterized by α-SMA upregulation, was reversed by H89, a PKA inhibitor, confirming that PTHrP_1-34_ promotes fibroblast-to-myofibroblast differentiation via PTH1R/PKA signaling. Given the role of subepithelial fibroblasts in epithelial regeneration and structural maintenance, our findings underscore the importance of paracrine signaling between bronchial epithelial PTHrP and fibroblast PTH1R in maintaining airway integrity. Collectively, these results highlight the therapeutic potential of targeting PTHrP/PTH1R signaling via peptide antagonism, gene silencing, or neutralization as a promising strategy to prevent the progression of PTHrP-related fibrotic diseases, including IPF.

Previous studies have demonstrated that PTHrP_1-34_ levels decrease during the acute phase of lung injury. In a silica-induced lung injury model, Hastings et al. reported reduced PTHrP_1-34_ expression on day 4, followed by an increase on day 14.^49^ As mentioned earlier, Stern et al. reported that PTHrP levels in the BALF were negatively correlated with the severity of lung injury.^18^ In another clinical study, preoperative PTHrP levels in the BALF were significantly lower in patients who subsequently developed postoperative lung injury after pulmonary thromboendarterectomy, suggesting that reduced PTHrP expression may predispose to epithelial vulnerability. They proposed that PTHrP may contribute to epithelial repair, given its role as an autocrine growth and differentiation factor for alveolar type II cells.^50^ Decreased PTHrP levels have also been observed in the tracheal aspirates of newborns with bronchopulmonary dysplasia or respiratory distress syndrome, suggesting a link to impaired lung maturation.^9,51^ In contrast, our study demonstrated a progressive increase in PTHrP_1-34_ levels in both bronchial epithelium and BALF during the fibrotic phase (day 15) of BLM-induced lung injury in mice. These findings suggest that PTHrP may exert stage-dependent functions during the transition from injury to fibrosis. In the acute phase, downregulation of PTHrP may facilitate epithelial repair, while its restoration supports homeostasis. However, sustained activation of the PTHrP pathway under chronic conditions, such as IPF, may drive fibroblast activation and ECM deposition. This dual role of PTHrP is consistent with the biphasic behavior observed in other signaling pathways involved in lung injury and remodeling. For example, M2 macrophages and amphiregulin promote epithelial repair in acute lung injury but contribute to fibrosis when chronically activated via TGFβ1 or PDGF signaling.^52–54^ Likewise, the Wnt/β-catenin and hypoxia-inducible factor-1 alpha pathways support AT2 cell regeneration after acute injury, yet their persistent activation in chronic hypoxia or IPF perpetuates fibrotic remodeling.^55–58^ Taken together, the apparent discrepancy between the acute and chronic effects of PTHrP reflects its context-dependent functional plasticity. This biphasic expression pattern highlights the potential of PTHrP_1-34_ as a stage-specific biomarker for lung injury and IPF, with implications for disease monitoring and therapeutic intervention.

Smooth muscle plays a vital role in maintaining the structural and functional integrity of the bronchial and vascular compartments in the lung. Its abnormal proliferation and contractility are implicated in the pathogenesis of various pulmonary diseases, including asthma, COPD, pulmonary hypertension, and fibrosis.^59^ Although the PTHrP/PTH1R axis has been shown to regulate vascular smooth muscle cell proliferation,^60,61^ its function in airway smooth muscle cells remains poorly defined. In this study, PTHrP_1-34_ did not affect proliferation, calcium influx, migration, or fibrosis gene expression in HBSMCs. Moreover, it failed to activate canonical PTH1R downstream pathways such as MAPK, PKA, and AKT, suggesting cell type-specific signaling divergence. Previous studies have demonstrated that PTHrP contributes to smooth muscle relaxation in various organ systems, including the vasculature, gastrointestinal tract, uterus, and urinary bladder.^62–65^ While our study did not directly assess the role of PTHrP in regulating bronchial smooth muscle tone, single-cell RNA sequencing revealed high expression of PTH1R in smooth muscle cells, supporting the possibility of its involvement in contractile regulation. Taken together, our findings suggest that the PTHrP/PTH1R axis does not promote proliferation or fibrotic remodeling in bronchial smooth muscles but may instead play a role in modulating smooth muscle tone. Further studies are warranted to elucidate the potential relaxant effects of PTHrP in airway smooth muscles and its implications in airway hyperresponsiveness and remodeling in chronic lung diseases.

Despite these promising findings, this study has certain limitations that warrant further investigation. To evaluate the clinical relevance of PTHrP expression in IPF, future studies must incorporate large, well-characterized patient cohorts that include comprehensive clinical parameters—such as forced vital capacity, diffusing capacity of the lung for carbon monoxide, imaging-based fibrosis extent, and progression-free survival. Such integrative analyses are essential to establish PTHrP as a viable biomarker and therapeutic target in IPF. In addition, the absence of cell type-specific genetic models limits our ability to identify the precise cellular sources of PTHrP in fibrotic lungs. Follow-up studies using bronchial epithelial cell-specific knockout mice (e.g., *Krt17*-Cre) and conditional deletion of PTH1R in fibroblasts and smooth muscle cells (e.g., *Col1a1*-Cre, *Pdgfrb*-Cre) will be critical to elucidate the role of the PTHrP_1-34_/PTH1R axis and epithelial–mesenchymal crosstalk in IPF pathogenesis. Last, while the BLM-induced PF model provides useful insights, it does not fully recapitulate the complexity of human IPF. Validation of our therapeutic strategies, including PTHrP neutralization, RNAi and PTH1R antagonism, should be extended to alternative models such as silica-induced fibrosis and humanized SCID mice to better reflect clinical scenarios.^66,67^

In conclusion, our study demonstrated that PTHrP_1-34_, derived from bronchial epithelial cells, drives PF by promoting fibroblast-to-myofibroblast transition and ECM production. Inhibition of the PTHrP_1-34_/PTH1R axis using α-PTHrP antibodies, PTHrP_7-34_, or RNAi effectively attenuated fibrosis in the BLM-induced PF model. These findings identify PTHrP_1-34_ as a key paracrine mediator in the pathogenesis of PF and a promising therapeutic target for IPF.

## Materials and methods

### Mice and BLM-induced pulmonary fibrosis

Eight- to ten-week-old male C57BL/6 mice were obtained from OrientBio (SeongNam, Korea). To establish a BLM-induced PF model, mice were anesthetized by intraperitoneal injection of 240 mg/kg 2,2,2-tribromoethanol (T48402, Sigma–Aldrich, St. Louis, MO, USA), followed by a single dose of BLM sulfate (B3972, Tokyo Chemical Industry Co., Ltd, Tokyo, Japan). To evaluate the antifibrotic effect of PTHrP_1-34_/PTH1R inhibiting agents in a BLM-induced PF model, we designed an experiment with approximately 6 to 10 animals per group based on the guidelines provided by the American Thoracic Society’s official workshop report.^28^ In the BLM-induced PF animal experiment, blinding was not applied, and mice were randomly assigned to experimental conditions. To evaluate the short-term effects of PTHrP_1-34_, mice received a single administration of PTHrP_1-34_ peptide dissolved in PBS at a dose of 0.5 mg/kg. Animals were sacrificed 1 or 2 days post-administration, and lung tissues were collected to assess fibrotic gene expression. For long-term evaluation, PTHrP_1-34_ (0.5 mg/kg) was administered every 3 days for a total of 10 doses, and mice were sacrificed 3 days after the final dose. To investigate the exacerbating effect of PTHrP_1-34_ on BLM-induced PF, mice were first treated with BLM (0.75 mg/kg) to induce mild fibrosis, allowing for a clearer evaluation of PTHrP_1-34_–mediated disease aggravation. Starting on day 3 post-BLM, PTHrP_1-34_ peptide (0.5 mg/kg) was administered every 3 days for a total of 4 doses. Mice were sacrificed 3 days after the final administration. To examine endogenous PTHrP expression and secretion, mice were administered BLM (1 mg/kg) and sacrificed at 1, 3, 7, or 15 days post-treatment. The antifibrotic efficacy of a PTHrP_1-34_ neutralizing antibody (1 mg/kg) was evaluated in 2 treatment models following BLM administration (1 mg/kg): preventive (starting on day 3 post-BLM, 2 doses) and therapeutic (starting on day 6 post-BLM, 3 doses). In each model, mice were sacrificed 3 days after the final antibody administration to assess therapeutic outcomes. To assess survival improvement, mice received a higher dose of BLM (2 mg/kg) and were treated with the PTHrP_1-34_ neutralizing antibody (1 mg/kg) every 3 days starting on day 6 for a total of 3 doses. The therapeutic potential of a PTHrP_7-34_ antagonist peptide (0.5 mg/kg) was tested by initiating treatment on day 6 post-BLM (1 mg/kg), with 3 doses administered every 3 days. Comparative efficacy studies were performed using the PTHrP_1-34_ neutralizing antibody (0.1 or 0.5 mg/kg), PTHrP_7-34_ (0.05 or 0.2 mg/kg), and nintedanib (30 mg/kg, daily for 10 days), all starting on day 6 post-BLM. Mice were sacrificed 3 days after the final dose of PTHrP_7-34_ or PTHrP_1-34_ neutralizing antibody and 1 day after the final dose of nintedanib. Finally, to evaluate the antifibrotic effect of Pthlh knockdown, mice received 2 administrations of lentivirus expressing shRNA targeting Pthlh spaced 3 days apart. Three days after the final viral dose, BLM (1 mg/kg) was administered, and the mice were monitored for 15 days. In all animal experiments, BLM, lentivirus, PTHrP_1-34_, PTHrP_7-34_, and the PTHrP_1-34_ neutralizing antibody were administered via IT instillation using a tracheal intubation technique. Nintedanib was administered orally.

### Enzyme-Linked Immunosorbent Assay (ELISA)

The concentrations of PTHrP in equal volumes of lung homogenates, plasma, bronchoalveolar lavage fluid (BALF), and cell culture supernatants were quantified using a PTHrP_1-34_ ELISA kit (S-1227; BMA Biomedicals, Augst, Switzerland). Briefly, 50 µL of each sample, standard, and control were added to the wells of a pre-coated microplate and incubated at room temperature for 60 min. Wells were then thoroughly washed with the provided ELISA buffer to eliminate non-specific binding. Subsequently, 100 µL of enzyme-linked detection antibody was added to each well and incubated for an additional 60 min at room temperature. After a second wash step, 100 µL of TMB solution was added and allowed to react for approximately 10 min. The reaction was terminated by adding 100 µL of stop solution, and absorbance was measured at 450 nm using a microplate reader. The detection range of the PTHrP_1-34_ ELISA kit was 0–10 ng/mL, with a sensitivity of 0.3 ng/mL. Cross-reactivity was 0% with PTHrP fragments 7–34, 38–64, 67–86, 107–138, and 109–141, and less than 1% with human and rat parathyroid hormone (PTH). The concentrations of TGFβ1 in equal volumes of cell culture supernatant were quantified using a human TGFβ1 ELISA kit (BMS249-4; Invitrogen, Carlsbad, CA, USA). The detection range of the TGFβ1 ELISA kit was 31–2000 pg/mL, with a sensitivity of 8.6 pg/mL. To analyze PTHrP_1-34_ levels in lung tissue homogenates from both mice and humans, frozen lung tissues (30 mg per sample) were weighed and transferred into pre-chilled tubes containing 300 μL of ice-cold lysis buffer (PBS supplemented with a protease inhibitor cocktail). Tissues were homogenized using a microtube tissue homogenizer until a uniform suspension was obtained. The freeze and thaw process was performed for 2 cycles. The homogenates were then centrifuged at 5000 × *g* for 10 min at 4 °C to remove cellular debris. The supernatants were collected, and total protein concentration was determined using the Bradford protein assay. Equal amounts of protein (0.1 mg) were used for ELISA. ELISA results were normalized to the total protein content of each sample. This step was performed to account for differences in tissue mass and ensure accurate comparisons across groups. Furthermore, to minimize any residual variability in protein concentration or sample handling, the measured analyte concentration (ng/ml) was divided by the corresponding total protein concentration for each sample. BALF was collected from mice following terminal anesthesia using an established tracheal cannulation technique. Briefly, mice were anesthetized via intraperitoneal injection of 222-tribromoethanol (240 mg/kg). Upon confirmation of deep anesthesia, a midline cervical incision was made to expose the trachea. A small incision was then made in the trachea, and a sterile polyethylene catheter (outer diameter: 0.86 mm) was inserted. The lungs were lavaged with sterile PBS containing 100 μM EDTA. A total volume of 1 mL was instilled, with each aliquot gently injected and withdrawn 3 times to maximize recovery. The recovered lavage fluid was collected into pre-chilled tubes and kept on ice until further processing. Typically, 70–80% of the instilled volume was recovered. BALF samples were centrifuged at 400 × *g* for 10 min at 4 °C to separate cells from the supernatant. The supernatant was stored at −80 °C and used for ELISA. Whole blood was collected and centrifuged to separate the plasma in as state where blood coagulation is prevented. The ELISA was performed according to the manufacturer’s protocol.

### Gene expression analysis (RT-qPCR)

Total RNA was isolated from cultured cells and snap-frozen lung tissues using TRIzol (Invitrogen, Carlsbad, CA, USA) and isopropanol (Sigma–Aldrich, St. Louis, MO, USA). The cells and tissues were lysed with TRIzol, and RNA was precipitated with isopropanol, followed by a wash with 75% cold ethanol. RNA purity was measured using a spectrophotometer (BioTek, Winooski, VT, USA) at 260 and 280 nm, with a ratio of approximately ~1.8 being accepted for performing quantitative PCR. A reverse transcriptase and cDNA synthesis kit (Applied Biosystems, Foster City, CA, USA) was used for cDNA synthesis. Quantitative PCR was performed using SYBR Green qPCR mixture (Applied Biosystems, Foster City, CA, USA) and run for 40 cycles on a LightCycler 96 System (Roche Diagnostics K.K., Tokyo, Japan). Gene expression was calculated using the 2 − ∆∆Ct method, with human *36B4* (ribosomal protein subunit P0, RPLP0) and mouse *β-actin* as reference genes. The sequences of primers used for quantitative PCR are described in Supplementary Table 1.

### Histological analysis

The harvested lung tissue was fixed in 4% formalin and then embedded in paraffin to produce tissue blocks. The paraffin-embedded tissues were sectioned at a thickness of 5 μm for histological analysis. Masson’s trichrome staining was performed using the Trichrome Stain Kit (ab150686; Abcam, Cambridge, UK) according to the manufacturer’s instructions. Briefly, the tissue sections were immersed in Bouin’s fluid for 60 min, stained with hematoxylin for 5 min, and then rinsed under running water for 2 minutes. Then the tissue sections were stained with fuchsin acid for 15 min and rinsed in distilled water. The sections were then stained with phosphomolybdic acid and aniline blue solution for 15 min, followed by wash in distilled water. After staining, the sections were treated with 1% acetic acid for 5 min, then dehydrated and mounted in synthetic resin. For hematoxylin and eosin (H&E) staining, the tissue sections were placed on slides and deparaffinized in serial baths of xylene and ethanol, followed by staining with hematoxylin and 0.2% eosin. For immunohistochemistry (IHC) staining in tissue sections, antigen retrieval was performed using target retrieval solution, pH 6.0 (Agilent, Santa Clara, CA, USA), in an autoclave for 20 min to remove the aldehyde links formed during the initial fixation of tissues. Non-specific antigen and antibody reactions were blocked using 5% BSA for 1 h at room temperature. The tissue sections were then incubated overnight at 4 °C with primary antibody against F4/80. For immunofluorescence (IF) staining in tissue sections, tissue sections were deparaffinized and rehydrated, followed by heat-induced epitope retrieval was performed by autoclaving at 121 °C for 10 min in 100 mmol/L citrate buffer (pH 6.0). The tissues were then treated with 3% hydrogen peroxide in methanol for 10 min. Non-specific antigen and antibody reactions were blocked using 5% BSA for 1 h at room temperature. The tissue slides were then incubated overnight at 4 °C with primary antibodies against α-SMA, PTHrP, KRT17 and p63. The next day, sections were incubated with fluorescent–conjugated secondary antibodies for 1 h at RT. After that, slides were stained with DAPI (4′,6-diamidino-2-phenylindole, Sigma-Aldrich, St. Louis, MO, USA) diluted 1:500 in PBS for 10 min. Finally, slides were mounted using mounting medium and covered with a cover glass. For IF staining in cultured cells, MRC5 cells were fixed with 4% paraformaldehyde for 20 min at room temperature, washed with PBS, and permeabilized with 0.2% Triton X-100 in PBS for 20 min. Cells were then blocked in PBS containing Tween 20 with 5% BSA for 60 min at room temperature. The fixed cells were incubated overnight at 4 °C with primary antibodies against α-SMA and COL1A1. The cells were then incubated for 60 min at room temperature with Fluorescent-conjugated secondary antibodies in 5% BSA in PBS-T. DAPI was used for nuclei staining for 10 min. A human lung interstitial fibrosis tissue microarray (LC561), purchased from US Biomax Inc. (MD, USA), was used. The tissue array included 23 cases of pulmonary fibrosis tissue and 4 cases each of cancer-adjacent lung tissue and normal lung tissue, with duplicate cores per case. The IF staining method was the same as that described above. Details of all antibodies used in this study, including dilution ratios, suppliers, catalog numbers, and experimental applications, are provided in Supplementary Table 2.

### Statistical analysis

Each data point shown in the results of gene expression, ELISA, cell number, and population percentages in cell-based experiments represents the average of technical duplicates. All animal experiments represent the average of biological replicates. All data are presented as the mean ± standard error of the mean (SEM). Statistical analysis was conducted using GraphPad Prism 10.6.1 (GraphPad Software, La Jolla, CA, USA). A two-tailed Student’s *t* test was used for single comparison. One-way or two-way ANOVA followed by Tukey’s post-hoc test was used for multiple comparisons. Statistical significance was indicated as *P < 0.05, **P < 0.01, and ***P < 0.001. Graphical abstract and schematic illustrations were created using BioRender (https://www.biorender.com/).

## Supplementary information

Supplementary materials

Supplementary materials 2

## Data Availability

All the data produced or analyzed in this study are provided within this published article and supplementary materials.
