# Supplementary material for: Parathyroid hormone–related protein is a therapeutic target in idiopathic pulmonary fibrosis
Source: Signal Transduct Target Ther. 2026 Feb 23;11:67. doi: 10.1038/s41392-026-02578-8 (PMC12926227; doi:10.1038/s41392-026-02578-8)

Supplementary Materials for

**Parathyroid hormone–related protein is a therapeutic target in**

**idiopathic pulmonary fibrosis**

Xue-Quan Fang^1,2,3#^, Suha Lim^4,5,6#^, Yoon-Mi Lee^1,3#^, Chang-Hoon Lim^1,2,3^, Han-Byeol Kim^7^, Jeong Ho Joo^8^, Sang-Woo Han^9^, Seohyun Kim^10^, Ji Hyung Kim^10^, Kwon Joong Na^11,12^,

Samina Park^11^, Young Tae Kim^11,12^, Jimyung Park^13^, Jooho Park^1,2,3^, Jeong Seok Lee^8^, Eun-Young Shin^7^, Eung-Gook Kim^7^, Hyun-Woo Shin^4,5,6,12,14*^, and Ji-Hong Lim^1,2,3*^

Correspondence to: jhlim@kku.ac.kr

**This PDF file includes:**

Parts of Materials and Methods

Supplementary Figures 1 to 8

Supplementary Tables 1 to 4

References

Uncropped western blots

**Other Supplementary Materials for this manuscript include the following:**

Demographics and clinical characteristics of patients in the IPF tissue microarray

***Materials and Methods***

**Transcriptome dataset and bioinformatics analysis**

Transcriptome dataset from the GSE213001, GSE134692, and GSE124685, which include human specimens from both healthy individuals and patients with idiopathic pulmonary fibrosis (IPF), were utilized for analysis^1-3^. Differential expression analysis (DEG) was conducted on pre-processed raw count data using the voom method from the limma package (version 3.64.0) in R. Kyoto Encyclopedia of Genes and Genomes (KEGG) pathway analysis were conducted using the clusterProfiler (version 4.16.0) package in the R, and the results were visualized using the ggplot2 (version 3.5.2) package^4^. Eligible differentially expressed genes (DEGs) were filtered with these criteria: adjusted p value < 0.05 and |log2 (Fold Change)| > 1. The DEGs were visualized using heatmaps generated with the R packages ComplexHeatmap (version 2.24.0) and ggplot2. Single cell RNA-sequencing (scRNA-seq) data of human bronchus were obtained from the Human Protein Atlas (HPA) database^5,6^. The pre-processed count matrix and curated cell-type annotations were directly used for downstream analysis. Data visualization, including dimensionality reduction and feature plots, was performed using Seurat (version 5.3.0) and ggplot2 (version 3.5.2) in R. Detailed information regarding data processing and quality control can be found at the HPA website (https://www.proteinatlas.org/about/celltype). To investigate gene expression at the single cell level in lung tissues from patients with IPF and healthy controls, we analyzed publicly available scRNA-seq datasets (GSE122960, GSE128033, and GSE135893) obtained from the Gene Expression Omnibus (GEO)^7-9^. For each dataset, we downloaded the filtered gene-barcode matrices, gene annotations, and cell barcodes. scRNA-seq data were processed and integrated using the Seurat package (version 4.3.0.1) in R studio. Initial Seurat objects were generated using the CreateSeuratObject function. For quality control, cells with fewer than 200 detected genes, or with greater than 25% of transcripts mapping to mitochondrial genes, were excluded. Genes not expressed in any cells were also removed. Each sample was normalized individually using the SCTransform package (version 0.3.5), and the resulting datasets were subsequently merged. To mitigate batch effects among samples, data integration was performed using the Harmony package (version 0.1.1). A total of 3,000 highly variable genes were selected to align cells across samples. Principal component analysis (PCA) was conducted using the top 40 principal components, followed by dimensionality reduction and visualization with Uniform Manifold Approximation and Projection (UMAP). Cells were clustered and annotated based on known standard marker genes. Following integration and annotation, epithelial cells were isolated by subsetting for canonical epithelial markers, and UMAP was re-applied to this subset for further analysis and visualization.

**Chemicals and Reagents**

Synthetic PTHrP peptides including PTHrP_1-34_ (4017147) and PTHrP_7_ (4031198) were purchased from Bachem (Bubendorf, Switzerland). The amino acid sequence of PTHrP_7-34_ amide is H-Leu-Leu-His-Asn-Leu-D-Trp-Lys-Ser-Ile-Gln-Asp-Leu-Arg-Arg-Arg-Phe-Phe-Leu-His-His-Leu-Ile-Ala-Glu-Ile-His-Thr-Ala-NH₂. Chemical formula is C_153_H_247_N_49_O_37_. Purity is greater than 96.0% as determined by HPLC. PTHrP_38-64_ (056-12), PTHrP_67-86_ (056-13) and PTHrP_107-138_ (056-15) were purchased from Phoenix Pharmaceuticals, Inc (Burlingame, CA, USA). Human PTHrP_1-86_ recombinant protein (SRP4651) was purchased from Sigma-Aldrich (St. Louis, MO, USA). Recombinant human TGFβ1 protein (240-B) was purchased from R&D Systems, Inc (Minneapolis, MN, USA). A selective TGFβ receptor type I/II (TβRI/II) dual inhibitor, LY2109761 (S2704), and Nintedanib (11022) were purchased from Selleckchem (Houston, TX, USA) and Cayman Chemical (Ann Arbor, MI, USA), respectively. Bleomycin sulfate (BLM, B3972) was purchased from Tokyo Chemical Industry Co., Ltd (Tokyo, Japan). H89 dihydrochloride hydrate (H89, B1427) and forskolin (FSK, F6886) were purchased from Sigma-Aldrich. PTHrP_1-34_ neutralizing antibody was purchased from BMA Biomedicals (T-4512; BMA Biomedicals, Augst, Switzerland). According to the supplier, this antibody exhibits cross-reactivity with human, mouse, and rat PTHrP_1-34_ and is validated for use in various experimental applications, including ELISA. Previous studies have demonstrated its efficacy in neutralizing PTHrP activity in both in vitro and in vivo models, supporting its utility in cellular and animal experiments aimed at investigating the functional role of PTHrP in disease pathogenesis^10-14^.

**Structure Prediction of the PTHrP_1-34_/PTH1R/G protein Complex**

The three-dimensional structure of a protein complex comprising the PTH1R, the G protein subunit α (Gα), subunit β (Gβ), and subunit γ (Gγ) and PTHrP_1-34_ was predicted using the AlphaFold Server (<https://alphafoldserver.com/>)^15^. To construct the input for structural prediction, protein sequences were retrieved from the UniProt database (PTH1R: Q03431; Gα: P63092; Gβ: P62873; Gγ: P59768; PTHrP: P12272)^16^. Each sequence was submitted as a single copy for multimeric structure modeling, enabling AlphaFold to predict the quaternary structure of the complex. The random seed parameter was set to 'auto' and 5 structural models were generated. Among these, the model with the highest ranking score was selected for subsequent structural assessment. To examine the resulting complex, including its overall conformation and intermolecular interactions, structural visualization and analysis were performed using BIOVIA Discovery Studio Visualizer (Dassault Systèmes, Vélizy-Villacoublay, France).

**Cell culture**

MRC5 (KCLB10171) human lung fibroblasts were obtained from the Korean Cell Line Bank (Seoul, Republic of Korea), and BEAS-2B (CRL-3588) human bronchial epithelial cells were obtained from ATCC (Manassas, VA, USA). MRC5 and BEAS-2B cells were cultured in Minimum Essential Medium (MEM) and RPMI-1640, respectively, supplemented with 10% fetal bovine serum (FBS; Gibco) and 1% penicillin-streptomycin (Gibco). Human primary bronchial smooth muscle cells (HBSMCs, C-12561) and human primary bronchial epithelial cells (HBEpCs, C-12640), both isolated from human bronchi, were obtained from PromoCell GmbH (Heidelberg, Germany). HBSMCs and HBEpCs were cultured in smooth muscle cell growth medium (C-22062) and airway epithelial cell growth medium (C-21060), respectively, each supplemented with a supplement mix (PromoCell GmbH, Heidelberg, Germany). To investigate hypoxia-induced changes in PTHrP expression, BEAS-2B and HBEpC cells were incubated for 24 hours at 37°C in a humidified atmosphere under normoxic (20% O₂ and 5% CO₂) or hypoxic (1% O₂ and 5% CO₂) conditions. Primary human IPF fibroblast cells (CC-7231) were cultured in Fibroblast Growth Medium-2 (FGM-2) (CC-3132) under standard conditions (Lonza, Basel, Switzerland). 2 hours before PTHrP_1-34_ treatment, the culture medium was replaced with Fibroblast Basal Medium (FBM). Primary human pulmonary alveolar epithelial cells (AECs) (#3200) were purchased from ScienCell Research Laboratories (Carlsbad, CA, USA) and cultured in Alveolar Epithelial Cell Medium (#3201). According to the manufacturer, these cells consist of both type I and type II alveolar epithelial cells.

**Isolation and culture of mouse primary lung fibroblasts (MPLFs) and alveolar type 2 (AT2) cells**

For isolation and culture of MPLFs, mouse lungs were perfused with 30 mL of cold PBS via the right ventricle to remove blood cells. The lungs were then excised, minced, and digested in serum-free DMEM/F12 medium containing Liberase (Sigma–Aldrich, St. Louis, MO, USA) and 1% penicillin-streptomycin at 37°C for 60 minutes. The digested lung tissues were neutralized with DMEM/F12 supplemented with 10% FBS, followed by centrifugation at 1500 rpm for 5 minutes. The resulting tissue pellets were washed 3 times with PBS and incubated in DMEM/F12 containing 10% FBS to allow the outgrowth of fibroblasts from lung tissue fragments. After 1 week, the cells were trypsinized and cultured in MEM supplemented with 10% FBS, 1% non-essential amino acids (NEAA), and 1% penicillin-streptomycin to promote the selection and proliferation of MPLFs. For isolation and culture of mouse primary alveolar type 2 cells, perfused mouse lungs were instilled via the trachea with 1 mL of Dispase II (5 U/mL; Sigma–Aldrich, St. Louis, MO, USA), followed by 0.5 mL of melted 2% agarose in PBS. The lungs were placed on ice for 2 minutes to allow the agarose to solidify and block the upper airways. Subsequently, the lungs were excised and digested in 3 mL of PBS containing Dispase II (2 U/mL) at room temperature for 45 minutes. After digestion, the lungs were minced and transferred to 3 mL of serum-free DMEM containing DNase I (100 µg; Sigma–Aldrich, St. Louis, MO, USA), followed by incubation at room temperature for 20 minutes. The digested tissue was neutralized with DMEM supplemented with 10% FBS, filtered sequentially through 40-µm nylon mesh filters, and centrifuged at 1,500 rpm for 5 minutes. The cell pellet was resuspended in MACS buffer (PBS containing 0.5% BSA and 2 mM EDTA) and subjected to negative selection using anti-CD45 microbeads (#130-052-301; Miltenyi Biotec, Bergisch Gladbach, Germany) to deplete hematopoietic cells. The CD45⁻ fraction was stained with PE-conjugated anti-EpCAM, washed, incubated with anti-PE microbeads (#130-048-801; Miltenyi Biotec), and enriched by positive selection using MACS columns according to the manufacturer’s instructions, yielding EpCAM⁺ AT2 cells. Purified AT2 cells were seeded in 24-well plates coated with a mixture of 70% rat-tail collagen I (Corning, NY, USA) and 30% Matrigel (Corning) at a density of 3 × 10⁵ cells per well. Cells were cultured in DMEM supplemented with 2% FBS, 1% penicillin–streptomycin, and 20 ng/mL keratinocyte growth factor (MedChemExpress, Monmouth Junction, NJ, USA) for 24 hours^17,18^.

**Conditioned medium**

To prepare conditioned medium (CM), BEAS-2B and HBEpC cells were seeded at 70% confluence and allowed to adhere overnight. BLM was added the next day to induce stress responses for 48 hours. After treatment, the medium was replaced with serum-free medium, and CM was collected 24 hours later. The CM was centrifuged at 1,500 × g for 5 minutes at 4 ℃ and filtered through a 0.22-μm membrane. For fibroblasts stimulation, cells were cultured in medium mixed 1:1 (v/v) with CM from BEAS-2B or HBEpC cells and incubated for 24 hours.

**Immunoblotting**

For immunoblotting, total proteins were extracted from the cultured cells and snap-frozen lung tissues using lysis buffer containing 1M Tris-HCl, 2.5M NaCl, 10% NP-40, 1% sodium deoxycholate (SDC), 1% SDS, and a protease inhibitor cocktail. A Bradford protein assay was conducted to determine total protein concentration. Total proteins were resolved using SDS-polyacrylamide gel electrophoresis (PAGE), transferred onto PVDF membranes (Millipore, Burlington, MA, USA), and blocked with 5% skimmed milk in Tris-buffered saline (TBS) containing Tween 20. The membranes were incubated overnight at 4°C with primary antibodies, followed by incubation at room temperature for 1 hour with corresponding HRP-conjugated secondary antibodies. Finally, protein expression was visualized using an ECL Prime kit (GE Healthcare, Milwaukee, WI, USA). Details of all antibodies used in this study, including dilution ratios, suppliers, catalog numbers, and experimental applications, are provided in Supplementary Table 2.

**Cloning, transfection and Luciferase reporter assay**

The full-length human PTH1R cDNA was amplified from the pCMV-SPORT6-PTH1R plasmid (Korea Human Gene Bank, KRIBB, Korea) using PCR with primers containing NheI and EcoRV restriction sites. The PCR product and the pLenti-GIII-CMV lentiviral vector (Applied Biological Materials Inc, Richmond, BC, Canada). were digested with NheI and EcoRV restriction enzymes at 37°C for 1 hour. The digested fragments were purified and ligated using T4 DNA ligase at room temperature for 16 hours. The resulting construct, pLenti-GIII-CMV-PTH1R, was verified by sequencing. The primer sequences (5’-3’) of subcloning were 5’-CTAGCTAGCATGGGGACCGCCCGGATCGCACCC-3’ and 5’-CCGGATATCTCACATGACTGTCTCCCACTC-3’. For parathyroid hormone 1 receptor (PTH1R) knockdown, 20 nM of small interfering RNA (siRNA) against PTH1R was transiently transfected into MRC5 cells using Lipofectamine™ RNAi MAX (Invitrogen, Carlsbad, CA, USA). After transfection, the cells were incubated for an additional 48 hours to allow for stabilization and gene silencing. The sequences of siRNA and shRNA used for gene silencing are described in Supplementary Table 3. HEK293 cells were transfected with pLenti-PTH1R using Lipofectamine 2000 according to the manufacturer’s instructions. 48 hours post-transfection, cells were subjected to selection with puromycin (2 μg/mL) for 10 days to establish stable expression. HEK293-PTH1R were seeded in 12-well plates and transfected with a CRE-luciferase reporter vector (Promega, E8471). After transfection, cells were subjected to overnight serum starvation and subsequently treated with various concentrations of the peptide or neutralizing antibody for 5 hours. Luciferase activity was measured using the Luciferase Assay System (Promega, E1501) and normalized to β-galactosidase activity. EC_50_ or IC_50_ values were calculated using nonlinear regression analysis in GraphPad Prism 10.6.1.

**Human frozen lung tissues**

Human control lung tissues were obtained from the non-tumorous regions of adenocarcinoma patients, and IPF lung tissues were obtained from patients who underwent surgical lung biopsy. Frozen lung tissues were pulverized into a fine powder while maintaining cryogenic conditions using liquid nitrogen and dry ice. Clinical information of human donors is summarized in Supplementary Table 4.

**Cell viability**

To assess cell viability, HBEpCs, AECs and mouse primary AT2 (mAT2) cells were incubated for 24 hours in the presence of either vehicle or PTHrP_1-34_. Following incubation, cells were washed with cold PBS and fixed with 4% paraformaldehyde. Fixed cells were stained with 0.5% crystal violet solution for 20 minutes at room temperature. After staining, crystal violet was solubilized using 1% sodium dodecyl sulfate (SDS) solution, and the optical density (OD570) was measured using a microplate reader (BioTek, Winooski, VT, USA).

**Calcium Quantification in Mouse Whole Blood**

Calcium concentrations in mouse whole blood were measured using a colorimetric calcium quantification kit (Sigma–Aldrich, Cat# MAK477), according to the manufacturer’s instructions. Briefly, whole blood samples were collected and incubated with the calcium assay reagent at room temperature for 3 minutes. Absorbance was measured at 612 nm using a microplate reader, and calcium levels were calculated using the formula provided by the manufacturer.

**Intracellular Calcium Imaging**

Intracellular calcium in HBSMCs were monitored using the Fluo-4 Calcium Imaging Kit (F10489; Thermo Fisher Scientific, Waltham, MA, USA) according to the manufacturer’s instructions. Briefly, HBSMCs were incubated with the Fluo-4 AM loading solution at 37°C for 20 minutes, followed by an additional 20 minutes at room temperature. After incubation, cells were washed once with PBS and replaced with HBSS. HBSMCs were stimulated with PTHrP_1-34_ (100 ng/mL) for 1 or 3 minutes. KCl (50 mM) was applied as a positive control. Fluorescence images were acquired using an inverted fluorescence microscope (DMi8 Manual, Leica Microsystems, Wetzlar, Germany) equipped with a FITC filter set (excitation = 494 nm, emission = 506 nm).

**Hydroxyproline assay for collagen quantification**

The total hydroxyproline content in the right lungs of mice was measured using a hydroxyproline assay kit (MAK008; Sigma–Aldrich, St. Louis, MO, USA). Lung tissues were weighed, homogenized with water, and then hydrolyzed with 12N HCl at 120°C for 3 hours. The hydroxyproline concentration was determined by reacting oxidized hydroxyproline with 4-(Dimethylamino) benzaldehyde (DMAB), resulting in a colorimetric product at 560 nm proportional to the hydroxyproline content.

***In vivo* Transduction by intratracheal (IT) intubation of a Lentivirus**

pLKO.1-shRNA-Pthlh (TRCN0000179093 and TRCN0000180583, Sigma-Aldrich, St. Louis, MO, USA) was used to generate Pthlh knockdown in mice, as described previously^19^. To produce lentivirus harboring shRNA against Pthlh, the pLKO.1-shRNA vector, envelope vector (pMD2.G), and packaging vector (psPAX2) were transiently transfected into HEK293T cells using Lipofectamine 3000 reagent (Invitrogen, Carlsbad, CA, USA). The transfected HEK293T cells were incubated in culture medium containing 30% FBS for 48 hours to allow amplification of lentiviruses. The lentiviral particles were then concentrated and purified using a Lenti-X™ Concentrator (Takara, Shiga, Japan). The purified lentiviral stocks were quantified using the Lenti-X™ RT-qPCR titration kit (Takara, Shiga, Japan). To deliver control or Pthlh-silencing lentivirus to the bronchus and lung in vivo, lentiviruses for all groups (control, shPthlh#1, and shPthlh#2) at a dose of 1 × 10^8 PFU in a final volume of 50 μL were administered intratracheally twice, every 3 days. Three days after the final lentivirus instillation, BLM (1 mg/kg) was administered via tracheal intubation, and the mice were monitored for 15 days thereafter.

**Cell cycle analysis**

Cell cycle progression was assessed using the Click-iT Plus EdU Flow Cytometry Assay Kit (C10634; Thermo Fisher Scientific, Waltham, MA, USA) following the manufacturer’s instructions. HBSMCs were incubated with 10 µM EdU for 2 hours at 37°C, harvested by trypsinization, and washed in 1% BSA in PBS. Cells were then fixed with 4% paraformaldehyde in PBS for 15 minutes at room temperature. Fixed cells were then permeabilized using the saponin-based permeabilization and wash reagent. After permeabilization, cells were incubated with the Click-iT™ reaction cocktail for 30 minutes at room temperature in the dark. Following the reaction, nuclei were counterstained with DAPI (1 µg/mL), and fluorescence signals were analyzed using a BD FACS Canto II flow cytometer (Becton, Dickinson and Company, Franklin Lakes, NJ, USA).

**Wound healing and Transwell migration assay**

MRC5 cells were seeded at a density of 1×10^5 cells per well and cultured until they reached 90% confluence. The cell monolayer was scraped straight with a sterile pipette tip and washed 3 times with PBS to remove cell debris. The cells were then subjected to serum-free starvation for 2 hours, followed by incubation with vehicle or 100 ng/mL of PTHrP_1-34_ for 4 hours. After this, the cells were incubated in complete medium for an additional 24 hours, and the migrated cell images were obtained using Nikon Eclipse TS2 microscopy (Nikon, Japan). Transwell chamber (Sigma–Aldrich, St. Louis, MO, USA) was used for in vitro migration assay. MRC5 and HBSMCs, at a concentration of 1×10^4 cells in 0.2 mL of serum-free MEM and smooth muscle cells basal medium, were placed in the upper chamber either in the absence or presence of 100 ng/mL of PTHrP_1-34_. The lower chamber was filled with MEM containing 2% FBS and smooth muscle cells growth medium as a chemoattractant, and the cells were incubated for 24 hours. The migrated cells that attached to the lower side of the Transwell chamber membrane were fixed with 4% paraformaldehyde for 15 minutes and then stained with crystal violet for 15 minutes. The membrane was placed on a glass slide, and the total number of cells was quantified from 4 randomly selected fields under 40× magnification using a Nikon Eclipse TS2 (Nikon, Japan). Cells were counted in 20% of each field area and extrapolated to estimate the total number in the entire field.

***Supplementary Figures 1-8***

**
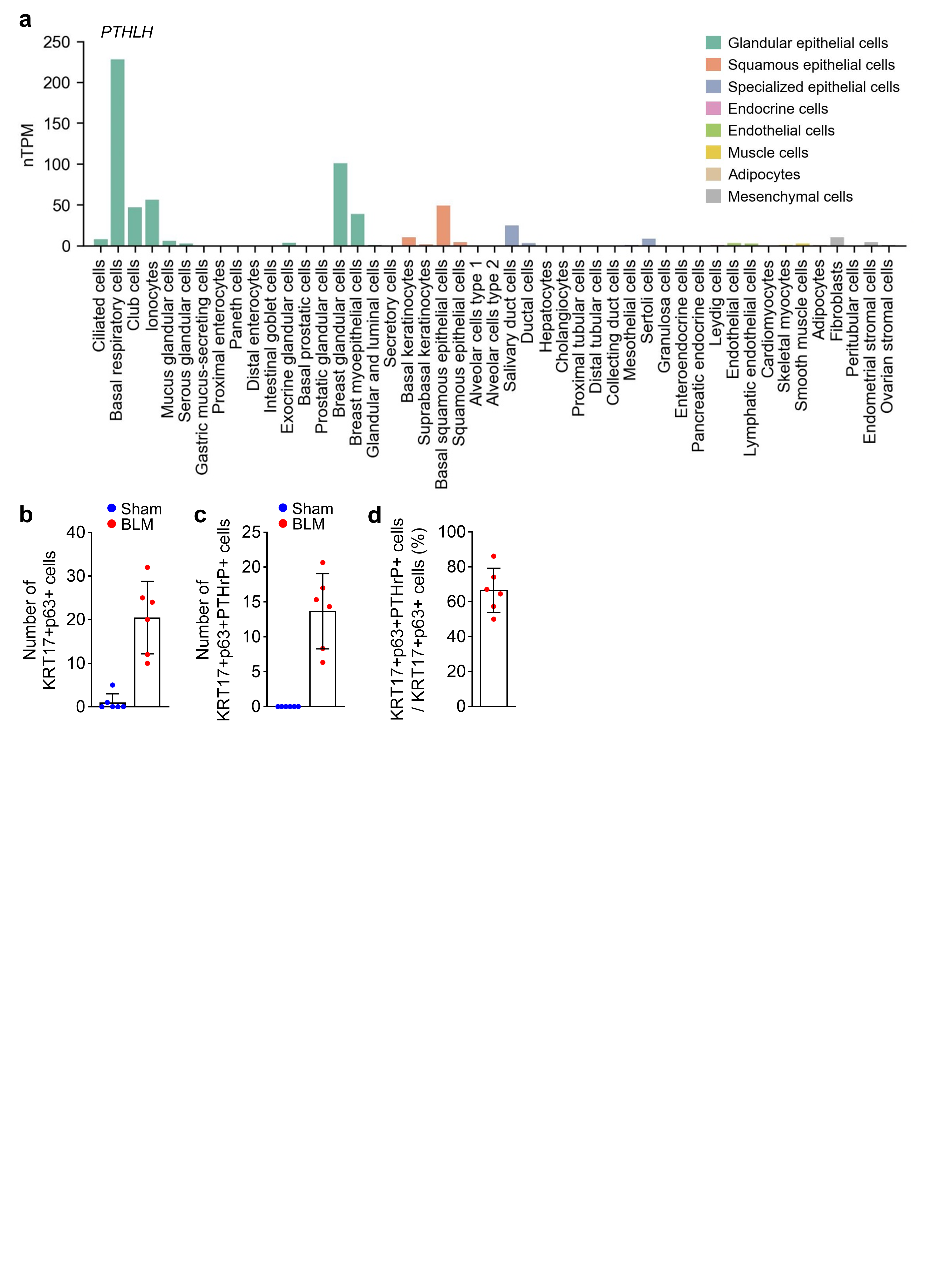
**

**Supplementary Figure. 1.** ***PTHLH* mRNA expression in human tissues.**

(a) *PTHLH* mRNA expression (nTPM; normalized Transcripts Per Million). Single-cell RNA-sequencing (scRNA-seq) data from human bronchial tissue were obtained from the Human Protein Atlas (HPA) database (https://www.proteinatlas.org/about/celltype). (b) Quantification of aberrant basaloid cells (KRT17+ and p63+). (c) Co-expression of aberrant basaloid cells with PTHrP (KRT17+, p63+, and PTHrP+). (d) Proportion of PTHrP+ cells among aberrant basaloid cells. Cell counting was performed on 3 randomly selected fields per subject at 200 × magnification, and the mean value was used for quantification.

**
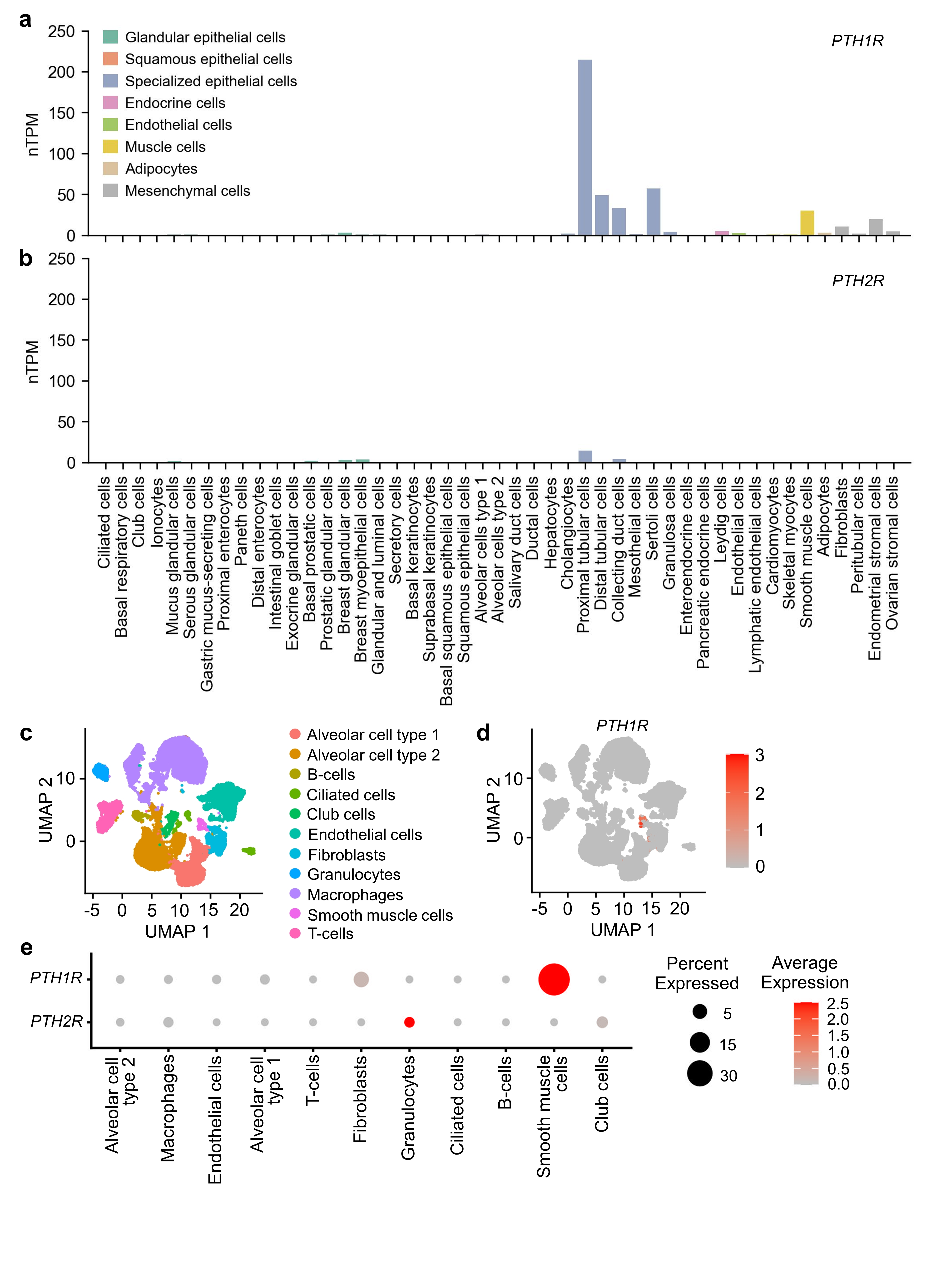
**

**Supplementary Figure. 2. Cell type-specific expression of *PTH1R* and *PTH2R* mRNA in human tissues.**

(a-b) Single-cell RNA-sequencing (scRNA-seq) data from human bronchial tissue were obtained from the Human Protein Atlas (HPA) database (https://www.proteinatlas.org/about/celltype). (a) *PTH1R* mRNA expression) (nTPM; normalized Transcripts Per Million. (b) *PTH2R* mRNA expression. (c-e) Single-cell RNA sequencing (scRNA-seq) data from human lung tissue were used for analysis. (c) UMAP visualization of single-cell populations in scRNA-seq data from human lung. (d) UMAP visualization of *PTH1R*-expressing single cells (red) in human lung. (e) Dot plot showing the expression of *PTH1R* and *PTH2R* in single cells from human lung.

**
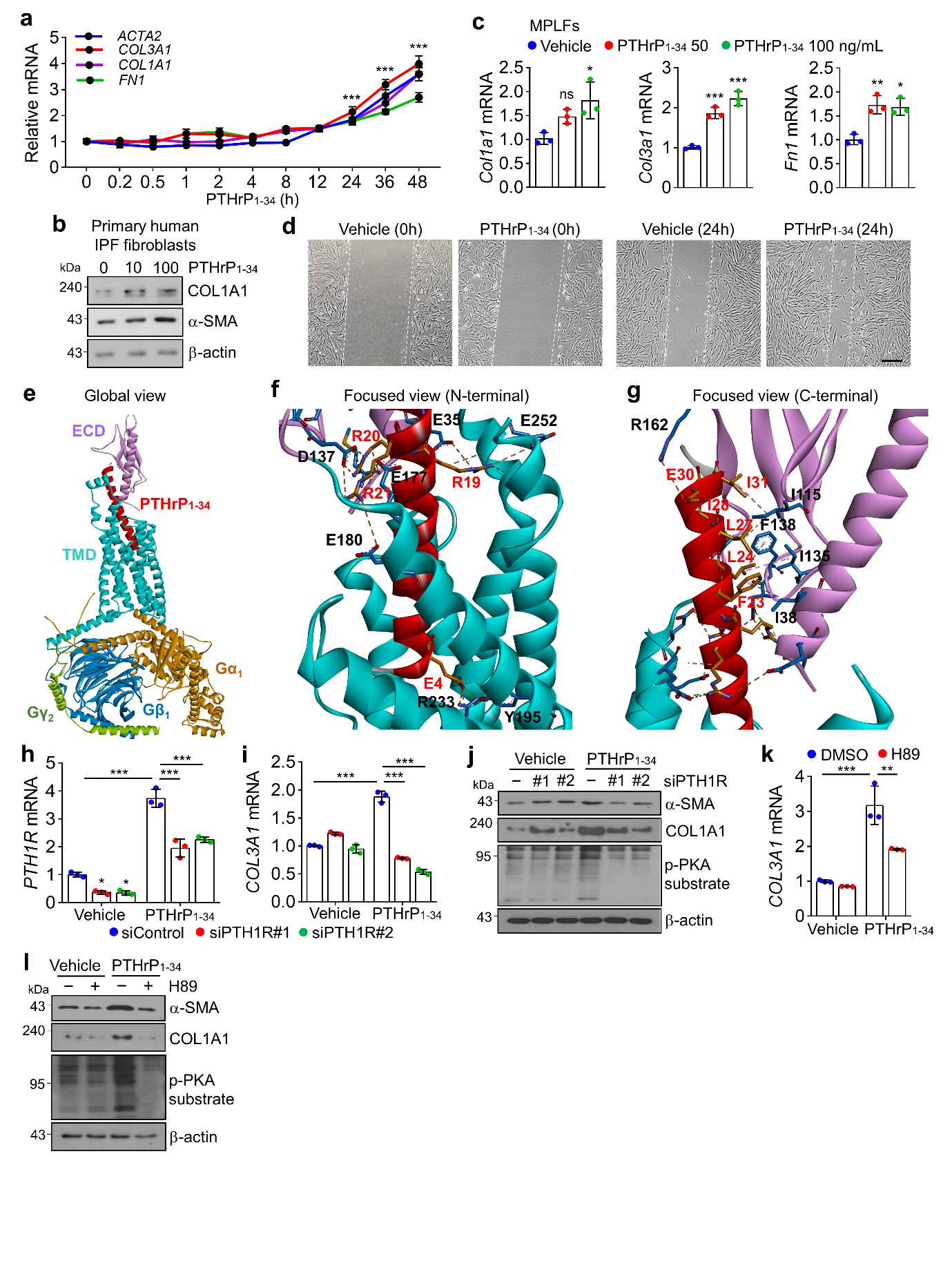
**

**Supplementary Figure. 3. PTHrP_1-34_ activates lung fibroblasts via the PTH1R/PKA signaling**

(a) PTHrP_1-34_ (100 ng/mL) treatment increases the expression of fibrosis-related genes (*ACTA2, COL3A1, COL1A1,* and *FN1*) in MRC5 cells in a time-dependent manner. (b) COL1A1 and α-SMA protein expression in human primary IPF fibroblasts upon PTHrP_1-34_ (10, and 100 ng/mL, 48 hours) treatment. (c) Expression of fibrosis-related genes in mouse primary lung fibroblasts (MPLFs). Cells were incubated for 48 hours with vehicle or PTHrP_1-34_ (50 and 100 ng/mL). (d) Representative images of migrated MRC5 cells in the absence or presence of PTHrP_1-34_ (100 ng/mL). Scale bar: 200 μm. (e) Overall structure of the PTH1R/PTHrP_1-34_/Gα_1_β_1_γ_2_ complex. (f) Interactions between the N-terminal region of PTHrP_1-34_ and the PTH1R TMD. (g) Interactions between the C-terminal region of PTHrP_1-34_ and the PTH1R ECD. (h-j) MRC5 cells were transiently transfected with siRNA silencing control (siControl, 20 nM) or PTH1R-targeting siRNAs (siPTH1R#1 and siPTH1R#2, 20 nM) prior to PTHrP_1-34_ treatment. Transfected cells were cultured with vehicle or PTHrP_1-34_ (100 ng/mL) for 48 hours. (h) *PTH1R* mRNA expression were measured using RT-qPCR. (i) *COL3A1* mRNA expression (j) α-SMA, COL1A1 and phosphorylated PKA substrate protein expression. (k-l) MRC5 cells were pretreated with the PKA inhibitor H89 (10 μM) for 1 hour prior to PTHrP_1-34_ treatment. After pretreatment, cells were cultured with PTHrP_1-34_ (100 ng/mL) for 48 hours. (k) *COL3A1* mRNA expression. (l) ɑ-SMA, COL1A1, and phosphorylated PKA substrate protein expression. Data are shown as mean ± SEM. *P*-values were analyzed by two-way ANOVA (a) and one-way ANOVA and Tukey’s test in (c, h, i, and k). *P < 0.05, **P < 0.01, ***P < 0.001. ns; not significant.

**
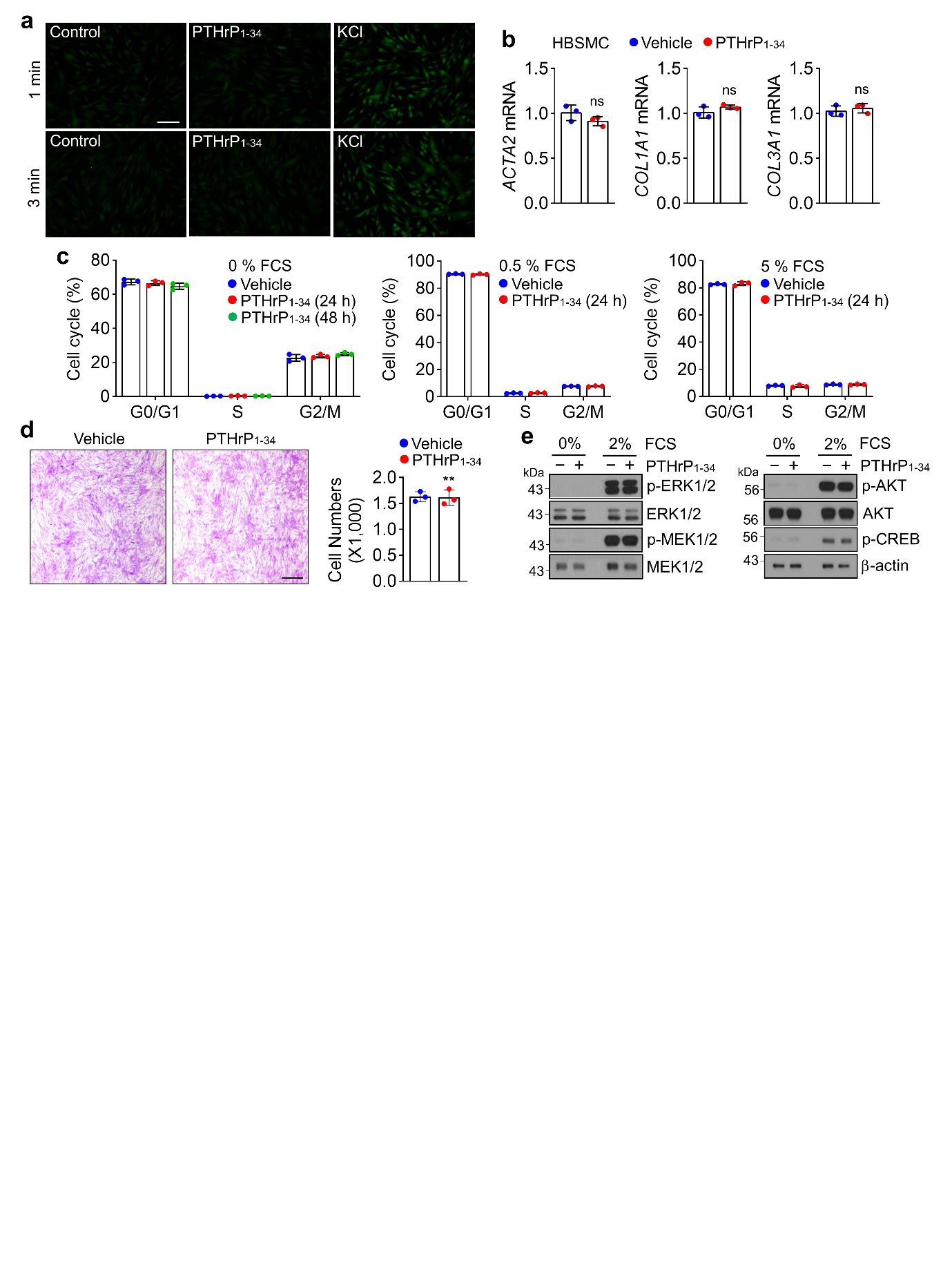
**

**Supplementary Figure. 4.** **PTHrP_1-34_ does not affect physiological functions in HBSMCs.** (a) Representative fluorescence images of Fluo-4 AM–loaded HBSMCs showing Ca²⁺ responses to PTHrP_1-34_ (100 ng/mL) or KCl (50 mM) stimulation at 1 minutes and 3 minutes. Scale bar: 200 μm. (b) Fibrosis-related genes in HBSMCs incubated with PTHrP_1-34_ (100 ng/mL, 48 hours). (c) EdU/DAPI-based flow cytometric analysis of cell-cycle distribution in HBSMCs treated with PTHrP_1-34_ (100 ng/mL) under different serum conditions (0, 0.5, and 5 % FCS). (d) Representative images of migrated HBSMCs stained with crystal violet, and quantification of migrated cell numbers after incubation with PTHrP_1-34_ (100 ng/mL, 24 hours). Scale bar: 200 μm. (e) Western blot analysis of the MAPK, PKA and AKT signaling pathways in HBSMCs treated with PTHrP_1-34_ (100 ng/mL) for 10 minutes, with or without 2% FCS. *P*-values were determined by two-tailed Student’s t-test in (b and d). **P < 0.01. ns; not significant.


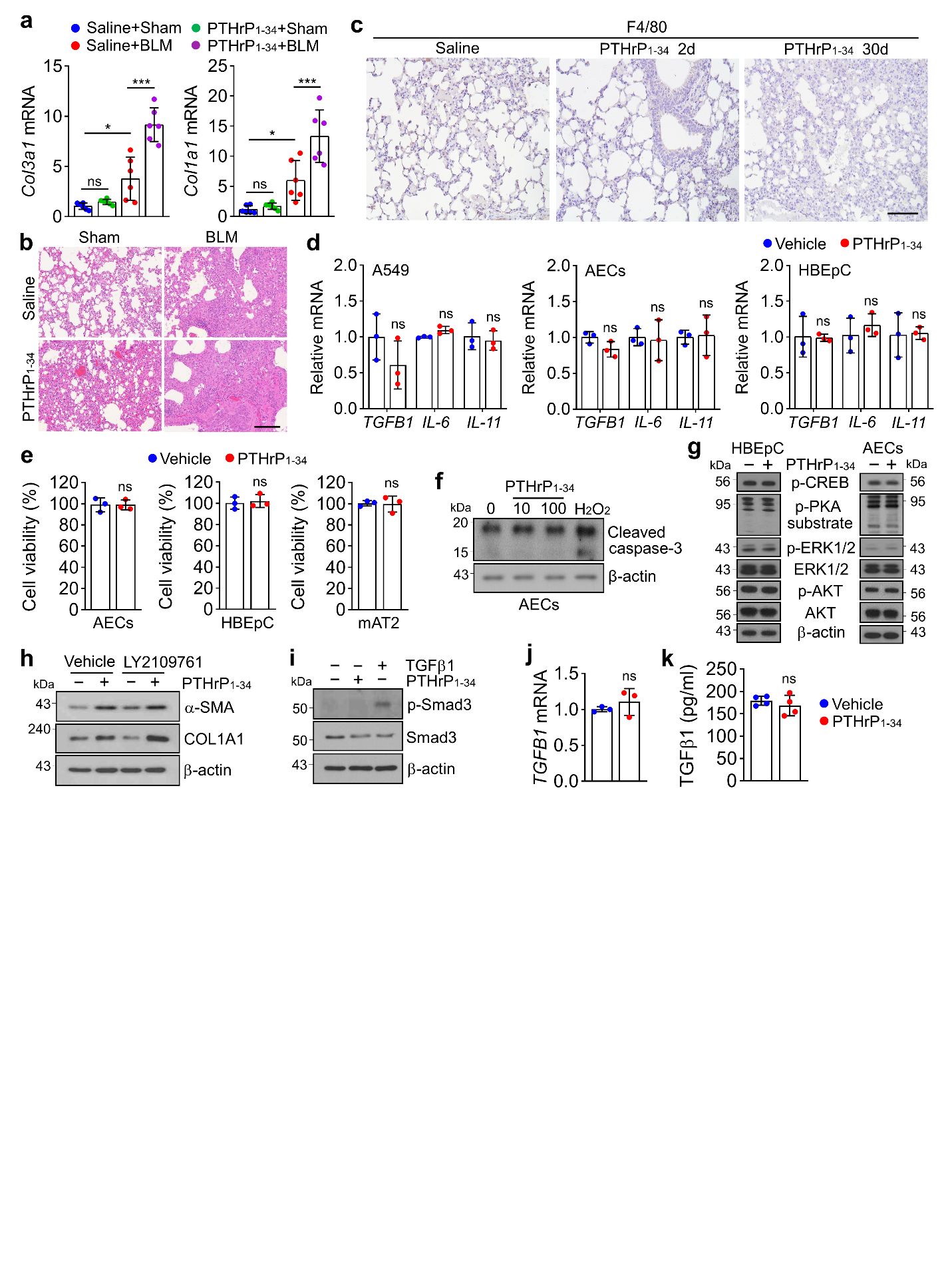


**Supplementary Figure. 5.** **PTHrP_1-34_ does not induce damage in bronchial and alveolar epithelial cells.**

(a-b) After injecting BLM (0.75 mg/kg) to the mice, PTHrP_1-34_ (0.5 mg/kg) was administered by intratracheal (IT) injection every 3 days for a total of 12 days, and the animal experiment was terminated 3 days after the final injection (n=6 each group, biological replicates). (a) Fibrosis-related genes in lung tissue. (b) H&E staining in lung sections. Scale bar: 100 μm. (c) F4/80 staining in lung sections. Scale bar: 100 μm. (d) Expression of profibrotic genes in A549 cells, AECs or HBEpCs treated with PTHrP_1-34_. Cells were incubated with vehicle or PTHrP_1-34_ (100 ng/mL) for 48 hours. (e) Quantitative analysis of cell viability based on crystal violet staining in AECs, HBEpCs, or mouse alveolar type 2 (mAT2) cells, presented as a bar graph. (f) Cleaved caspase-3 expression in human AECs in response to PTHrP_1-34_ (100 ng/mL, 24 hours) and H_2_O_2_ (100 μM, 6 hours). (g) Western blotting analysis of the MAPK, PKA and AKT signaling pathways in HBEpCs (left) or AECs (right) treated with PTHrP_1-34_ (100 ng/mL) for 10 minutes. (h) MRC5 cells were pretreated with the LY2109761 (5 μM) for 1 hour prior to PTHrP_1-34_ treatment. After pretreatment, cells were cultured with PTHrP_1-34_ (100 ng/mL) for 48 hours. Fibrosis-related proteins expression. (i) Phosphorylated Smad3 (p-Smad3) was analyzed by western blotting in MRC5 cells treated with PTHrP_1-34_ (100 ng/mL) or TGFβ1 (10 ng/mL) for 24 hours. (j) TGFβ1 mRNA expression in MRC5-treated with PTHrP_1-34_ (100 ng/mL) for 24 hours. (k) TGFβ1 protein expression in MRC5-treated with PTHrP_1-34_ (100 ng/mL) for 24 hours determined by ELISA. All data shown are mean ± SEM. *P*-values were determined by two-tailed Student’s t-test in (e, j and k) and one-way ANOVA Tukey’s test in (a, and d). *P < 0.05, ***P < 0.001. ns; not significant.


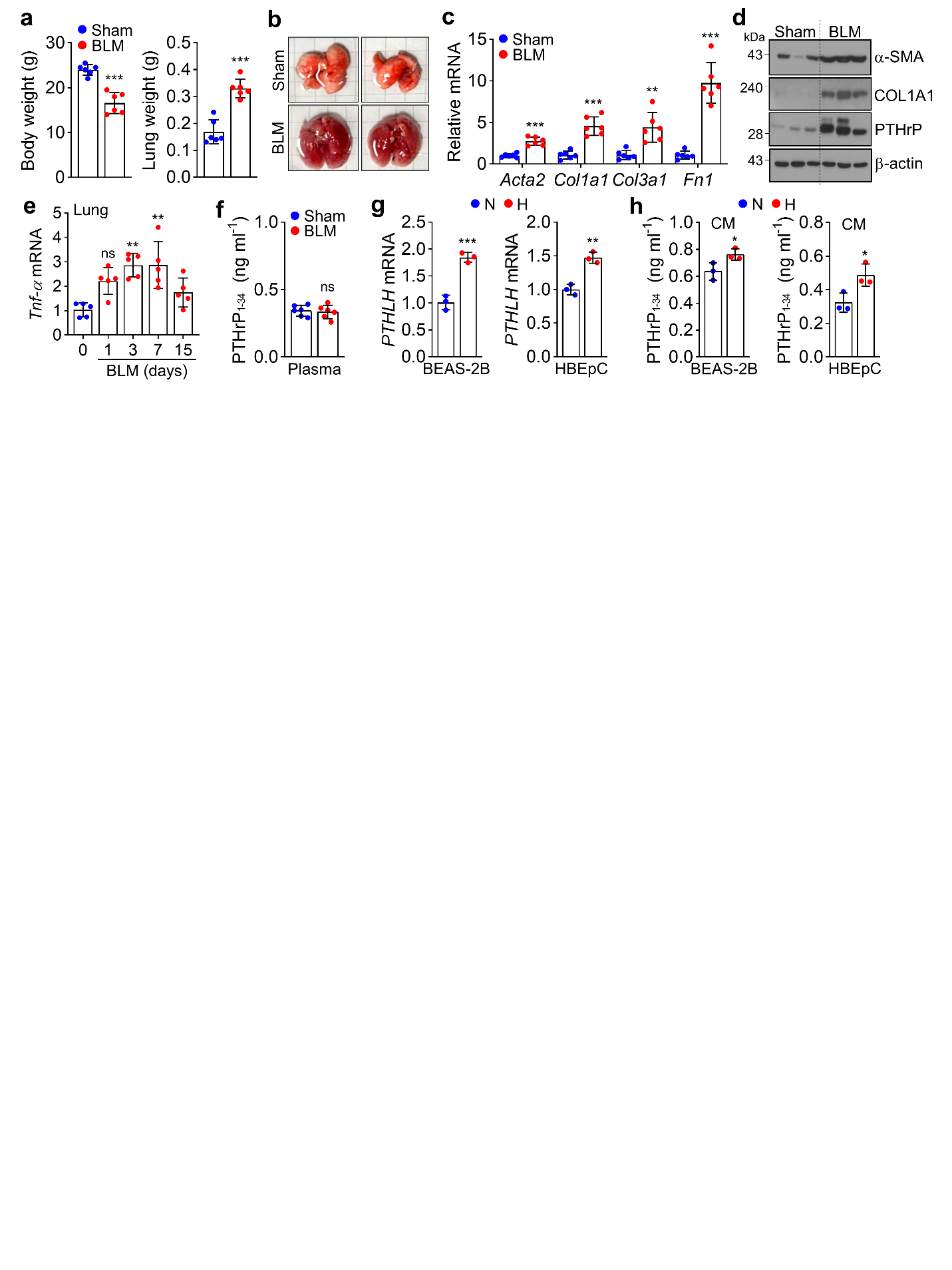


**Supplementary Figure. 6. PTHrP_1-34_ is secreted in response to BLM treatment or hypoxia.**

(a) Body and lung weight changes of mice injected with saline (sham) or 1 mg/kg of BLM (n=6, biological replicates). (b) Representative lung images from sham or BLM-induced PF mice (n=2 each group, biological replicates). (c) Expression of fibrosis-related genes in the lungs of sham or BLM-induced PF mice, measured by RT-qPCR (n=6, biological replicates). (d) Expression of fibrosis-related proteins in the lungs of sham or BLM-induced PF mice, measured by western blotting (n=3, biological replicates). (e) *Tnf-α* mRNA expression in BLM-induced PF mice (n=5, biological replicates) sacrificed at indicated time points. (f) Quantification of PTHrP_1-34_ in plasma in sham or BLM-induced PF mice (g) *PTHLH* mRNA expression in BEAS-2B or HBEpCs under normoxia (N) or hypoxia (H) for 24 hours. (h) PTHrP_1-34_ protein levels collected from conditioned medium of BEAS-2B or HBEpCs under hypoxia for 24 hours. All data shown are mean ± SEM. *P*-values were determined by two-tailed Student’s t-test in (a, c and g-h) and one-way ANOVA Tukey’s test in (e). *P < 0.05, **P < 0.01, ***P < 0.001. ns; not significant.

**
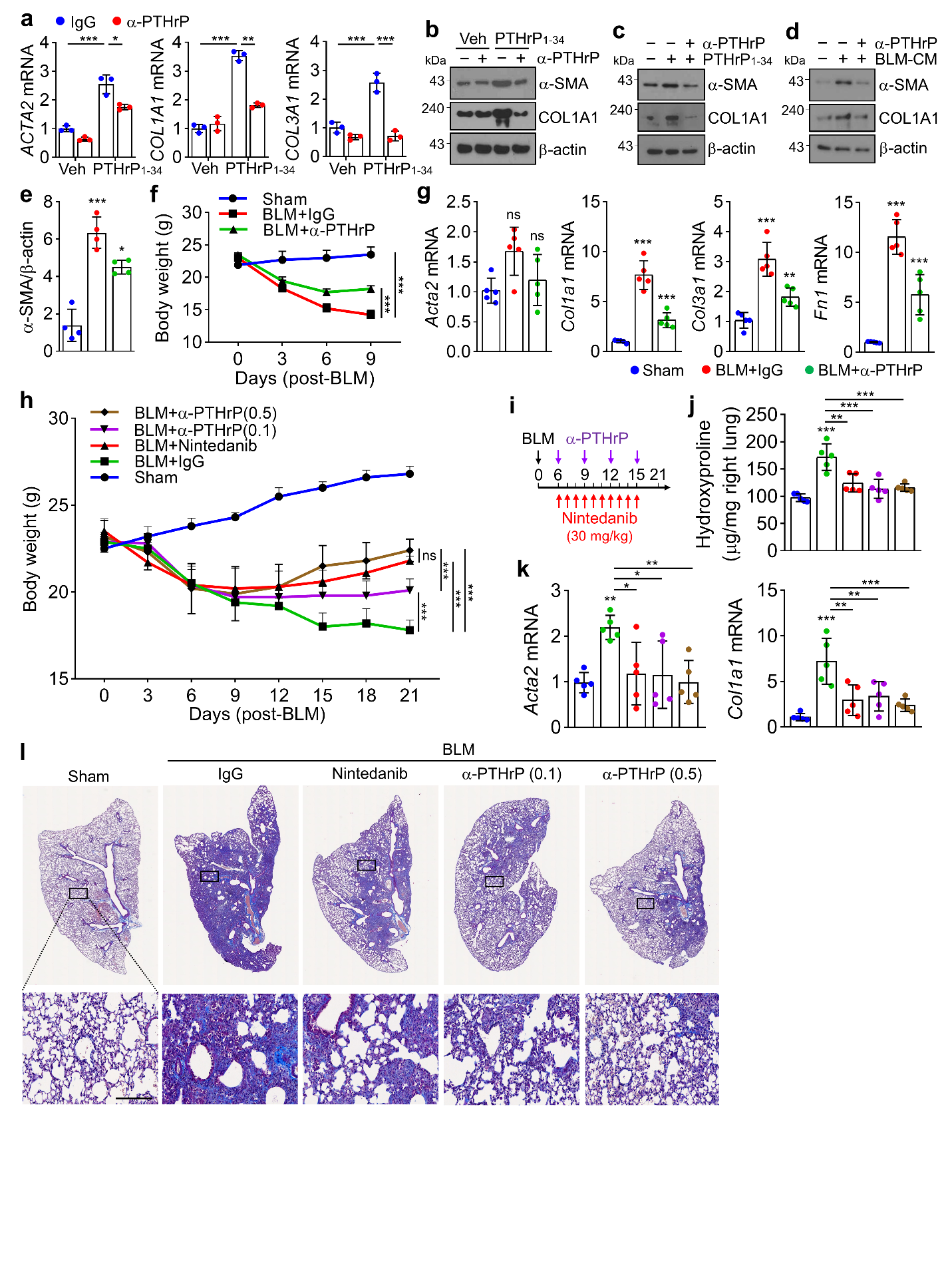
**

**Supplementary Figure. 7. Neutralization of PTHrP_1-34_ with α-PTHrP Antibody mitigates BLM-induced PF.**

(a–b) α-PTHrP (5 μg/mL) was used to pretreat MRC5 cells, which were then incubated with vehicle (Veh) or PTHrP_1-34_ (100 ng/mL, 48 hours). (a) Expression of fibrosis-related genes. (b) Expression of fibrosis-related proteins. (c) α-PTHrP (5 μg/mL) was used to pretreat human primary IPF fibroblasts, which were then incubated with Veh or PTHrP_1-34_ (100 ng/mL, 48 hours). (d) Conditioned medium from BLM (5 μg/mL, 48 hours)-treated BEAS-2B cells was mixed with α-PTHrP (5 μg/mL) and applied to MRC5 cells for analysis of fibrosis-related proteins. (e) Quantification of α-SMA protein in BLM-induced PF mice. (f, g) α-PTHrP (1 mg/kg) was administered via IT injection every 3 days for 6 days, starting on day 3 after BLM (1 mg/kg) challenge, and the animal experiment was terminated on day 9 (n=5 each group, biological replicates). (f) Changes in body weight of mice. (g) Expression of fibrosis-related genes in the lungs. (h-l) α-PTHrP (0.1 or 0.5 mg/kg) and nintedanib (30 mg/kg) were administered via IT injection every 3 days for 15 days, starting on day 6 after BLM (1 mg/kg) challenge, and the animal experiment was terminated on day 21 (n=5 each group, biological replicates) (h) Changes in body weight of mice (i) Schematic of the experimental schedule. (j) Hydroxyproline content in lung tissues. (k) Expression of fibrosis-related genes in lung tissues. (l) Masson’s trichrome in lung sections. Scale bar: 100 μm. Data are shown as mean ± SEM. *P*-values were analyzed by two-way ANOVA (f and h) and one-way ANOVA and Tukey’s test in (a, e, g, j and k). *P < 0.05, **P < 0.01, ***P < 0.001. ns; not significant.

**
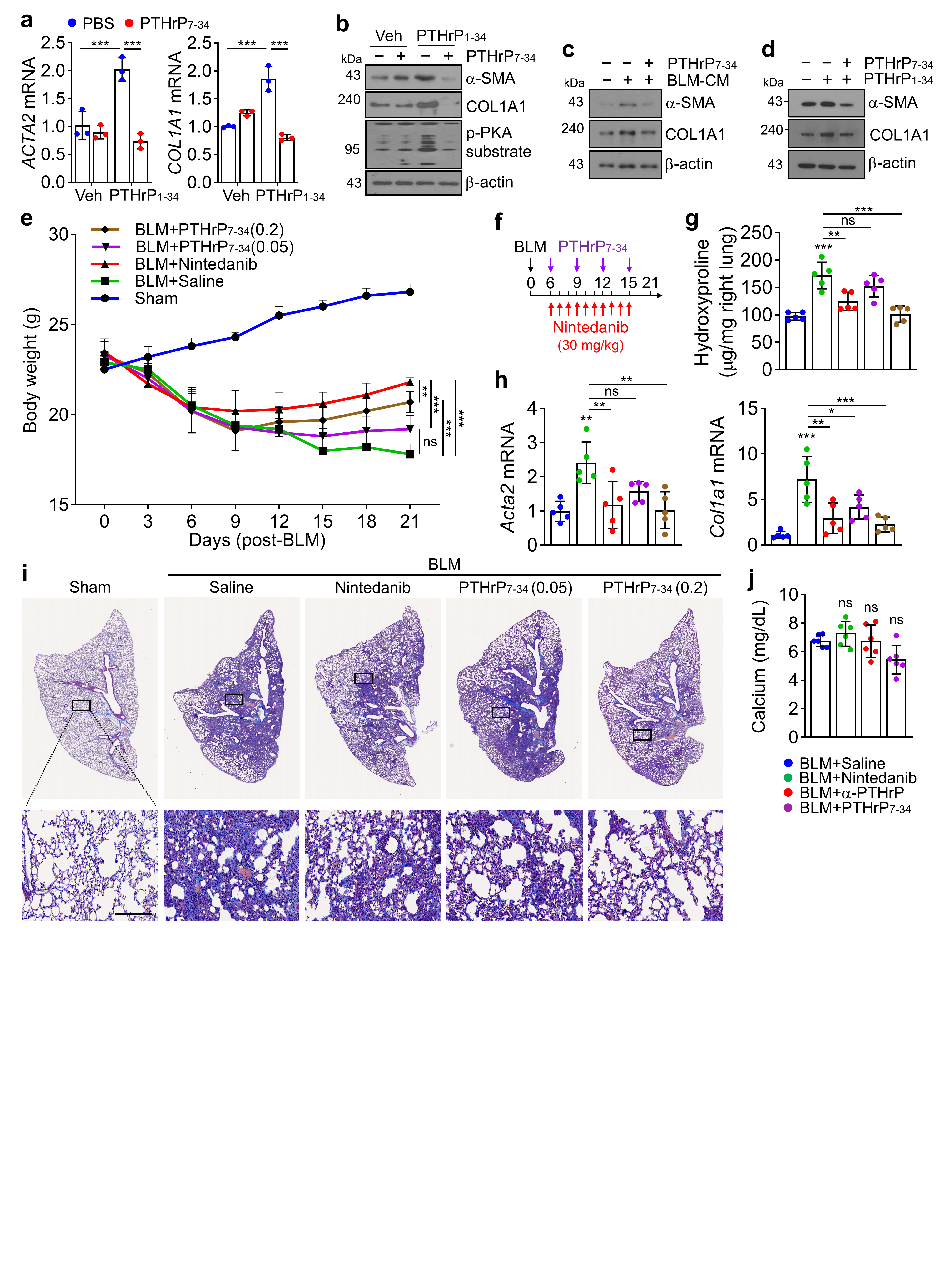
**

**Supplementary Figure. 8. PTHrP_7-34_ mitigates BLM-induced PF.**

(a and b) MRC5 cells were pretreated with PTHrP_7-34_ (100 ng/mL) for 1 hour prior to treatment with PTHrP_1-34_. The cells were then further cultured with PTHrP_1-34_ (100 ng/mL) for 48 hours. (a) Expression of fibrosis-related genes. (b) Fibrosis-related proteins and phosphorylated PKA substrate expression. (c) Conditioned medium from BEAS-2B cells treated with BLM (5 μg/mL, 48 hours) were mixed with PTHrP_7-34_ (100 ng/mL) and applied to MRC5 cells for analysis of fibrosis-related proteins. (d) Human primary IPF fibroblasts were pretreated with PTHrP_7-34_ (100 ng/mL) for 1 hour, followed by treatment with PTHrP_1-34_ (100 ng/mL) for 48 hours to evaluate fibrosis-related protein expression. (e-j) PTHrP_7-34_ (0.05 or 0.2 mg/kg) and nintedanib (30 mg/kg) were administered via IT injection every 3 days for 15 days, starting on day 6 after BLM (1 mg/kg) challenge, and the animal experiment was terminated on day 21 (n=5 each group, biological replicates). Data for Sham, BLM+Saline, and BLM+Nintedanib groups are shared between experiments shown in Supplementary Fig. 7h-7l. (e) Changes in body weight of mice. (f) Schematic of the experimental schedule. (g) Hydroxyproline content in lung tissues. (h) Expression of fibrosis-related genes in lung tissues. (i) Masson’s trichrome in lung sections. Scale bar: 100 μm. (j) Calcium levels were measured in whole blood collected from mice. Data are shown as mean ± SEM. *P*-values were analyzed by two-way ANOVA (e) and one-way ANOVA and Tukey’s test in (a, g, h, and j). *P < 0.05, **P < 0.01, ***P < 0.001. ns; not significant.

***Supplementary Tables 1-4***

**Supplementary Table 1. Nucleotide sequences for RT-qPCR primer**

| Gene | Forward Sequences (5’-3’) | Reverse Sequences (5’-3’) |
| --- | --- | --- |
| Acta2 (m) | ATGCAGAAGGAGATCACAGC | CAGCTTCGTCGTATTCCTGT |
| Col1a1 (m) | TGACTGGAAGAGCGGAGAGT | GTTCGGGCTGATGTACCAGT |
| Col3a1 (m) | TGACTGTCCCACGTAAGCAC | GGAGGGCCATAGCTGAACTG |
| Fn1 (m) | GAAGTCGCAAGGAAACAAGC | GCCACCATAAGTCTGGGTCA |
| Pthlh (m) | CATCAGCTACTGCATGACAAGG | CTGTGTGGATCTCCGCGAT |
| Pth1r (m) | TCTGCAATGGTGAGGTGCAG | GCTACTCCCACTTCGTT |
| β-actin (m) | ACGAGGCCCAGAGCAAGAG | TCTCCAAGTCGTCCCAGTTG |
| ACTA2 (h) | GTGACGAAGCACAGAGCAAA | ATGCTCTTCAGGGGCAACA |
| COL1A1 (h) | GGCAAGGTGTTGTGCGAT | TGGTTTCTTGGTCGGTGGG |
| COL3A1 (h) | CCACGGAAACACTGGTGGA | CGGCTGGAGAGAAGTCGAAG |
| FN1 (h) | ACCTCGGTGTTGTAAGGTGG | CCATAAAGGGCAACCAAGAG |
| PTHLH (h) | TTGTCATGGAGGAGCTGATG | CGGTGTTCCTGCTGAGCTAC |
| PTH1R (h) | GATTTACACCGTGGGCTACTC | CAGGTGCATGTGGATGTAGTT |
| TGFB1 (h) | TACCTGAACCCGTGTTGCTCTC | GTTGCTGAGGTATCGCCAGGAA |
| IL-6 (h) | AGTGAGGAACAAGCCAGAGC | GTCAGGGGTGGTTATTGCAT |
| IL-11 (h) | GGACAGGGAAGGGTTAAAGG | CTCAGCACGACCAGGACC |
| 36B4 (h) | TGGTGATACCTAAAGCCTGGAA | CATGTTGCTGGCCAATAAGG |

**Supplementary Table 2. Antibody information**

| Antibody | Supplier | Cat No | Application | Dilution |
| --- | --- | --- | --- | --- |
| α-SMA | Abcam | ab7817 | Immunoblotting | 1:1,000 |
| β-actin | Santa Cruz Biotechnology | sc-47778 | Immunoblotting | 1:1,000 |
| COL1A1 | Cell Signaling Technology | 72026 | Immunoblotting | 1:2,000 |
| Phospho-PKA substrate | Cell Signaling Technology | 9624 | Immunoblotting | 1:1,000 |
| Phospho-ERK1/2 | Cell Signaling Technology | 4370 | Immunoblotting | 1:5,000 |
| Phospho-MEK1/2 | Cell Signaling Technology | 9154 | Immunoblotting | 1:5,000 |
| Phospho-AKT | Cell Signaling Technology | 4060 | Immunoblotting | 1:2,000 |
| ERK1/2 | Santa Cruz Biotechnology | sc-94 | Immunoblotting | 1:2,000 |
| MEK1/2 | Cell Signaling Technology | 9122 | Immunoblotting | 1:2,000 |
| AKT | Cell Signaling Technology | 4691 | Immunoblotting | 1:2,000 |
| PTHrP | BMA Biomedicals | T-4512 | Immunoblotting | 1:1,000 |
| Smad3 | Cell Signaling Technology | 9523 | Immunoblotting | 1:1,000 |
| Phospho-Smad3 | Cell Signaling Technology | 9520 | Immunoblotting | 1:1,000 |
| Cleaved-caspase-3 | Cell Signaling Technology | 9664 | Immunoblotting | 1:1,000 |
| Anti-Rabbit-HRP | Jackson ImmunoResearch | 111-035-003 | Immunoblotting | 1:10,000 |
| Anti-Mouse-HRP | Jackson ImmunoResearch | 115-035-003 | Immunoblotting | 1:10,000 |
| α-SMA | Abcam | ab5694 | Immunofluorescence (IF) | 1:200 |
| KRT17 | Santa Cruz Biotechnology | sc-393002 | Immunofluorescence (IF) | 1:100 |
| p63 | R&D Systems | AF1916 | Immunofluorescence (IF) | 1:200 |
| COL1A1 | Cell Signaling Technology | 72026 | Immunofluorescence (IF) | 1:200 |
| PTHrP | BMA Biomedicals | T-4512 | Immunofluorescence (IF) | 1:200 |
| F4/80 | Proteintech | 29414-1-AP | Immunohistochemistry (IHC) | 1:10,000 |
| Alexa Fluor 488 | Thermo Fisher | A-21206 | Immunofluorescence (IF) | 1:1,000 |
| Alexa Fluor 555 | Thermo Fisher | A-31570 | Immunofluorescence (IF) | 1:1,000 |
| Alexa Fluor 647 | Thermo Fisher | A-32849 | Immunofluorescence (IF) | 1:1,000 |
| Alexa Fluor 594 | Thermo Fisher | A-11012 | Immunofluorescence (IF) | 1:1,000 |

**Supplementary Table 3. Nucleotide sequences for pLKO.1-shRNA and siRNA**

| Gene | Sense (5’-3’) | Antisense (5’-3’) |
| --- | --- | --- |
| shControl | GATACCTAACTCAGGAAACCA | TGGTTTCCTGAGTTAGGTATC |
| shPthlh#1 | GATACCTAACTCAGGAAACCA | TGGTTTCCTGAGTTAGGTATC |
| shPthlh#2 | CCAATTATTCCTGTCACTGTT | AACAGTGACAGGAATAATTGG |
| siControl | UUCUCCGAACGUGUCACGUTT | ACGUGACACGUUCGGAGAATT |
| siPTH1R #1 | GUUUCUGCAAUGGCGAGGUACAAGC | GACAAAGACGUUACCGCUCCAUGUUCG |
| siPTH1R #2 | AUGCAGAUGACGUCAUGACUAAAGA | CCUACGUCUACUGCAGUACUGAUUUCU |

**Supplementary Table 4. Patient information for control and IPF samples**

| Subject ID | Sex | Age | Smoking status |
| --- | --- | --- | --- |
| Control_1 | F | 55 | Non-smoker |
| Control_2 | M | 45 | Non-smoker |
| Control_3 | M | 57 | Non-smoker |
| Control_4 | F | 55 | Non-smoker |
| Control_5 | F | 37 | Non-smoker |
| IPF_1 | F | 61 | Non-smoker |
| IPF_2 | M | 64 | Ex-smoker |
| IPF_3 | M | 60 | Ex-smoker |
| IPF_4 | F | 64 | Non-smoker |
| IPF_5 | F | 65 | Non-smoker |

**References**

1 Jaffar, J. *et al.* Matrix metalloproteinase-7 is increased in lung bases but not apices in idiopathic pulmonary fibrosis. *ERJ Open Res* **8** (2022).

2 Sivakumar, P. *et al.* RNA sequencing of transplant-stage idiopathic pulmonary fibrosis lung reveals unique pathway regulation. *ERJ Open Res* **5** (2019).

3 McDonough, J. E. *et al.* Transcriptional regulatory model of fibrosis progression in the human lung. *JCI Insight* **4** (2019).

4 Kanehisa, M., Furumichi, M., Tanabe, M., Sato, Y. & Morishima, K. KEGG: new perspectives on genomes, pathways, diseases and drugs. *Nucleic Acids Res* **45**, D353-D361 (2017).

5 Karlsson, M. *et al.* A single-cell type transcriptomics map of human tissues. *Sci Adv* **7** (2021).

6 Lukassen, S. *et al.* SARS-CoV-2 receptor ACE2 and TMPRSS2 are primarily expressed in bronchial transient secretory cells. *EMBO J* **39**, e105114 (2020).

7 Habermann, A. C. *et al.* Single-cell RNA sequencing reveals profibrotic roles of distinct epithelial and mesenchymal lineages in pulmonary fibrosis. *Sci Adv* **6**, eaba1972 (2020).

8 Reyfman, P. A. *et al.* Single-Cell Transcriptomic Analysis of Human Lung Provides Insights into the Pathobiology of Pulmonary Fibrosis. *Am J Respir Crit Care Med* **199**, 1517-1536 (2019).

9 Morse, C. *et al.* Proliferating SPP1/MERTK-expressing macrophages in idiopathic pulmonary fibrosis. *Eur Respir J* **54** (2019).

10 Kir, S. *et al.* Tumour-derived PTH-related protein triggers adipose tissue browning and cancer cachexia. *Nature* **513** (2014).

11 Bhalerao, N. *et al.* Pancreatic cancer cachexia is mediated by PTHrP-driven disruption of adipose de novo lipogenesis. *bioRxiv* (2025).

12 Zhang, X., Cheng, Q., Wang, Y., Leung, P. S. & Mak, K. K. Hedgehog signaling in bone regulates whole-body energy metabolism through a bone-adipose endocrine relay mediated by PTHrP and adiponectin. *Cell Death Differ* **24**, 225-237 (2017).

13 Mak, I. W., Turcotte, R. E. & Ghert, M. Transcriptomic and proteomic analyses in bone tumor cells: Deciphering parathyroid hormone-related protein regulation of the cell cycle and apoptosis. *J Bone Miner Res* **27**, 1976-1991 (2012).

14 Wang, Y. *et al.* DLC1-dependent parathyroid hormone-like hormone inhibition suppresses breast cancer bone metastasis. *J Clin Invest* **124**, 1646-1659 (2014).

15 Abramson, J. *et al.* Accurate structure prediction of biomolecular interactions with AlphaFold 3. *Nature* **630**, 493-500 (2024).

16 UniProt, C. UniProt: the Universal Protein Knowledgebase in 2025. *Nucleic Acids Res* **53**, D609-D617 (2025).

17 Kebaabetswe, L. P., Haick, A. K. & Miura, T. A. Differentiated phenotypes of primary murine alveolar epithelial cells and their susceptibility to infection by respiratory viruses. *Virus Res* **175**, 110-119 (2013).

18 Chen, Q. & Liu, Y. Isolation and culture of mouse alveolar type II cells to study type II to type I cell differentiation. *STAR Protoc* **2**, 100241 (2021).

19 Richeldi, L., Collard, H. R. & Jones, M. G. Idiopathic pulmonary fibrosis. *Lancet* **389**, 1941-1952 (2017).

**Uncropped western blots**


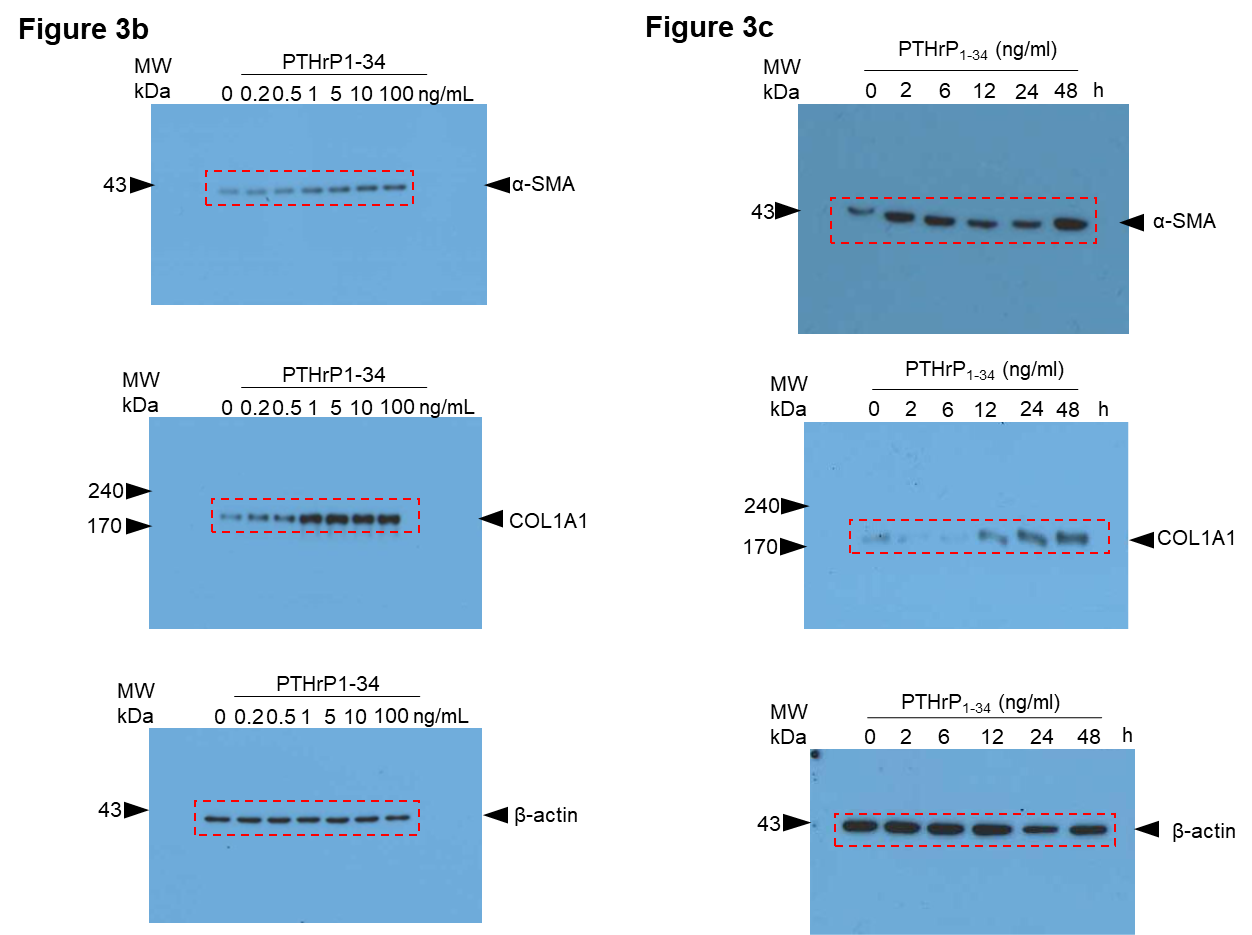


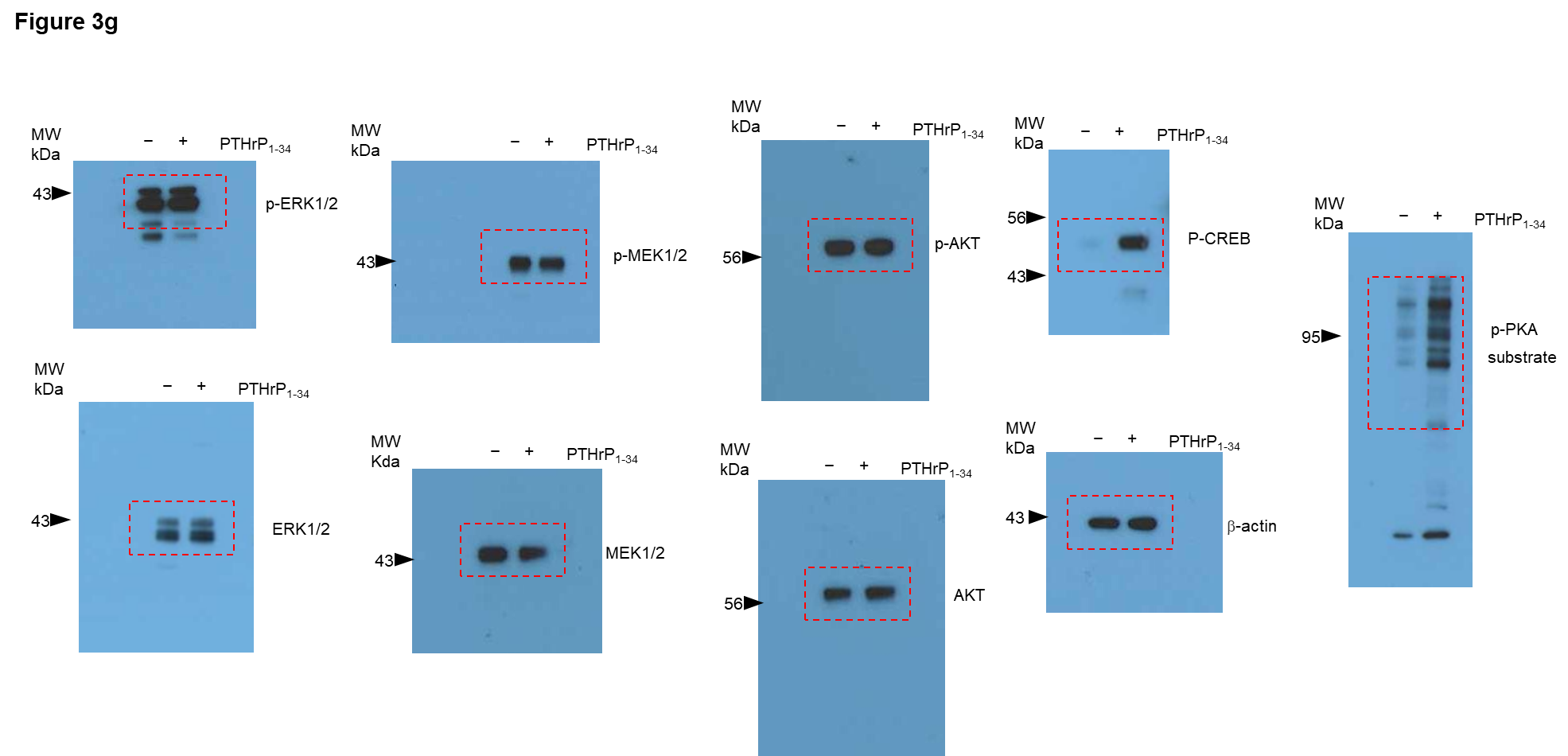


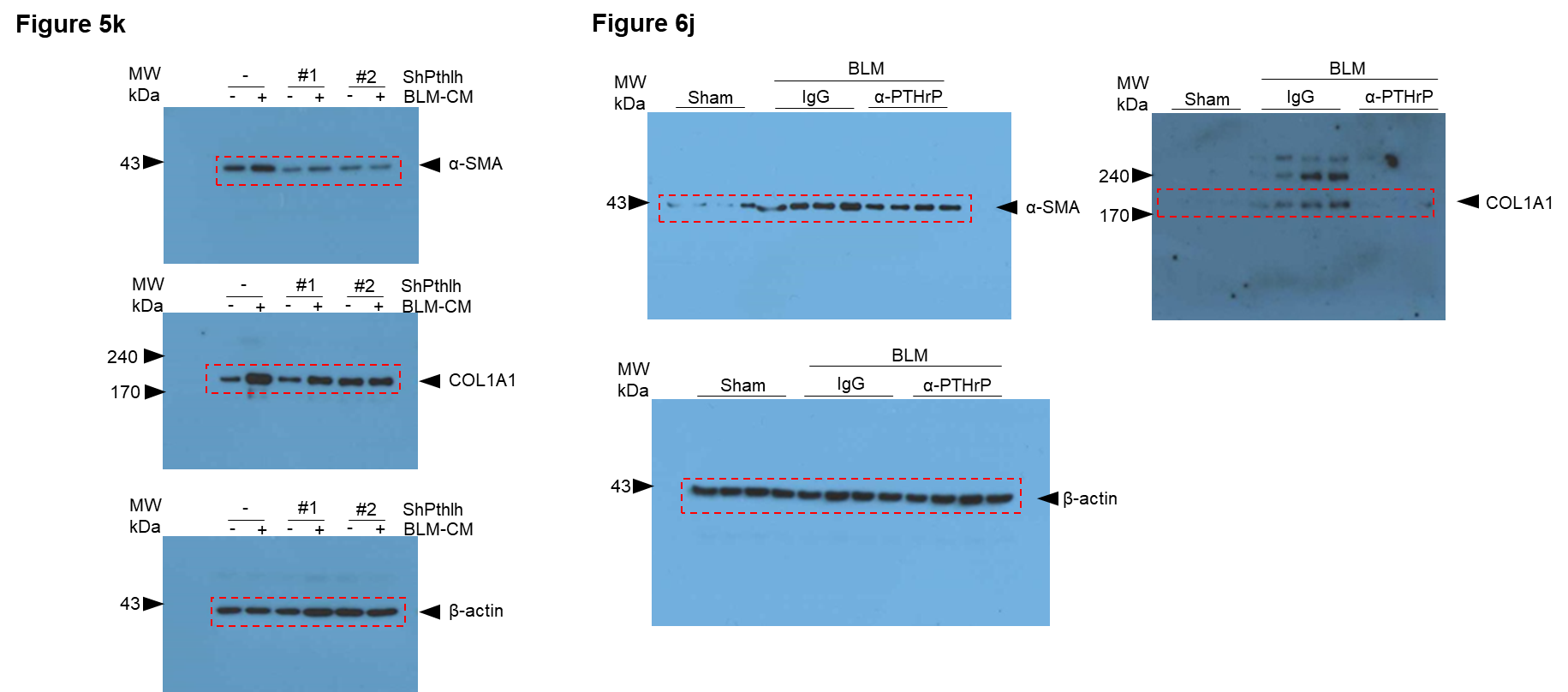


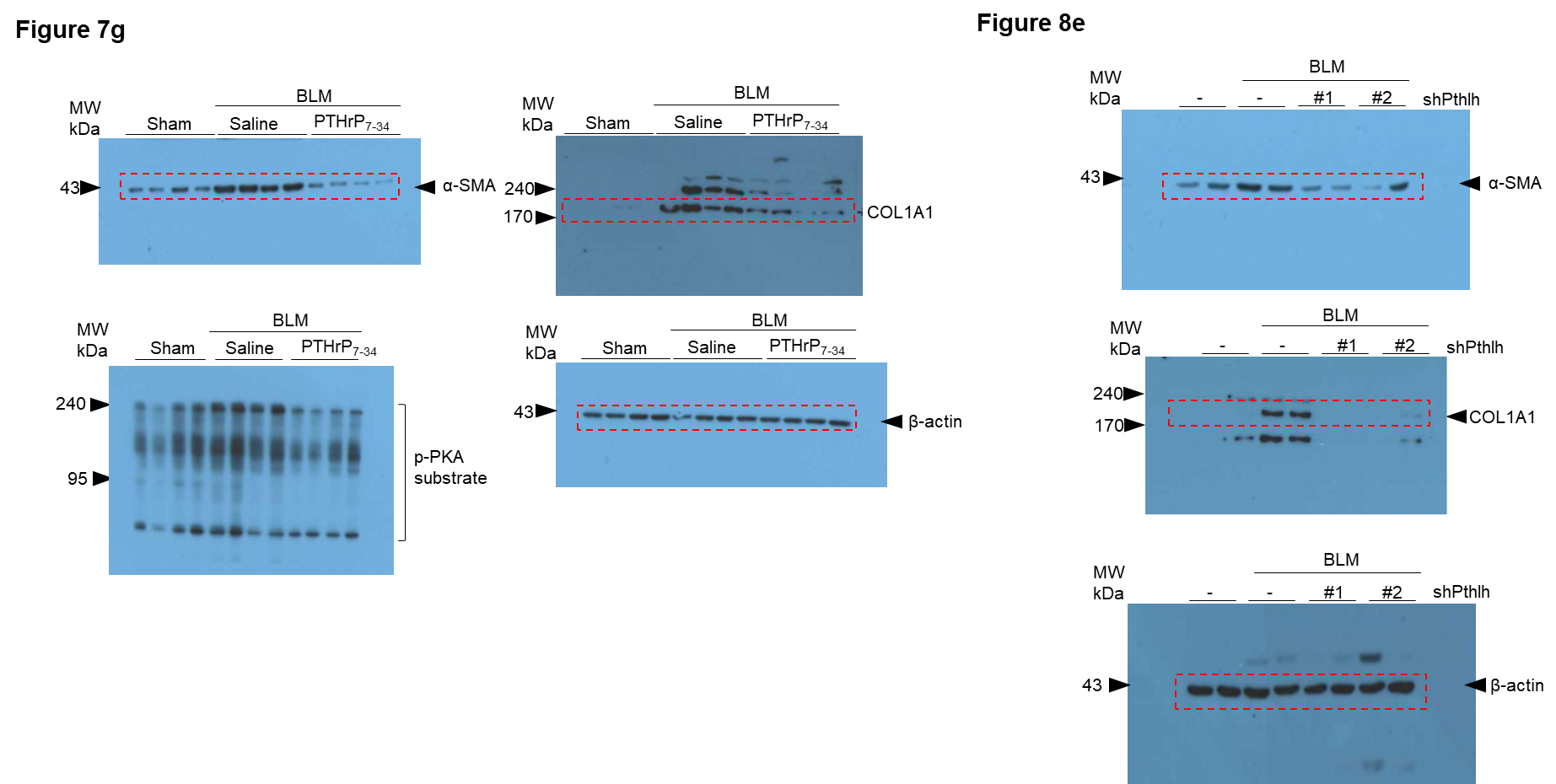


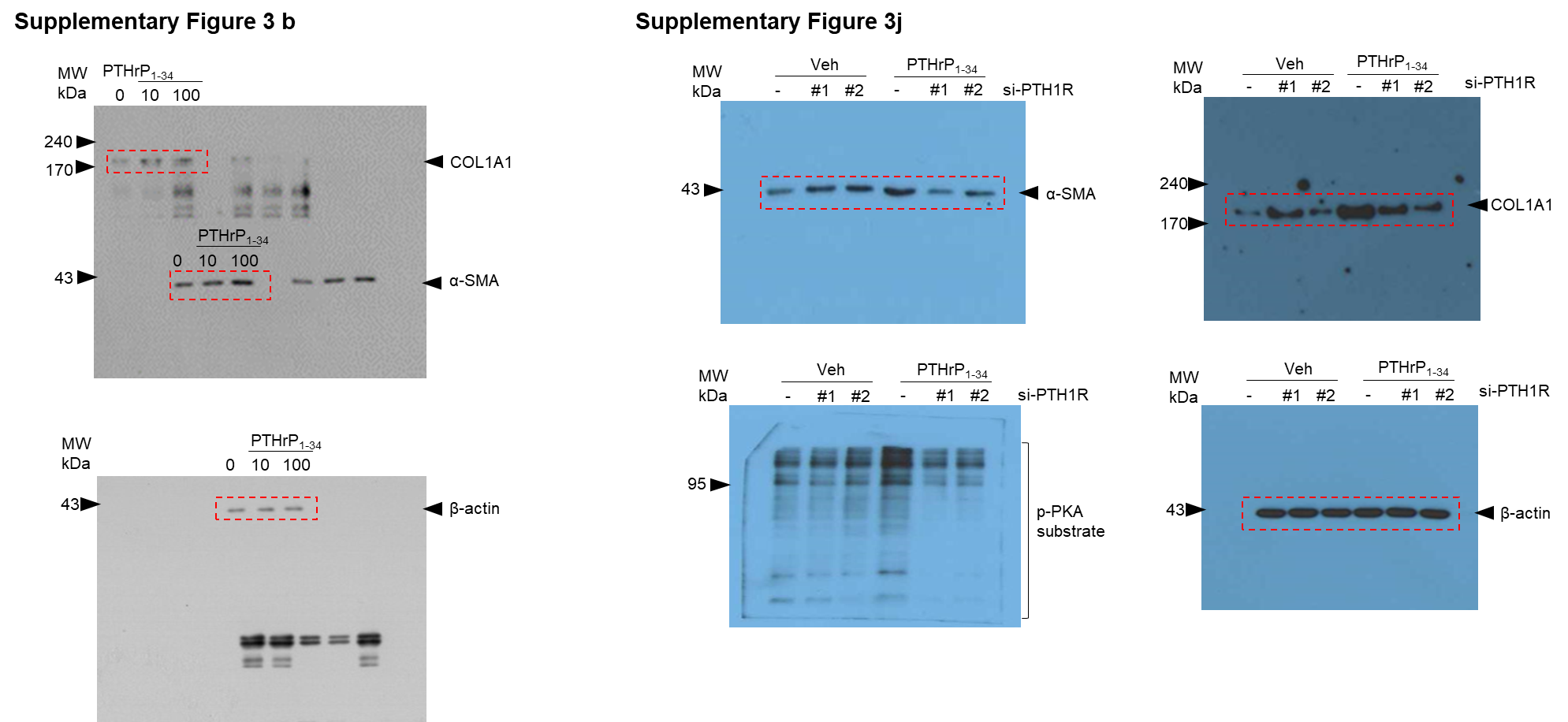


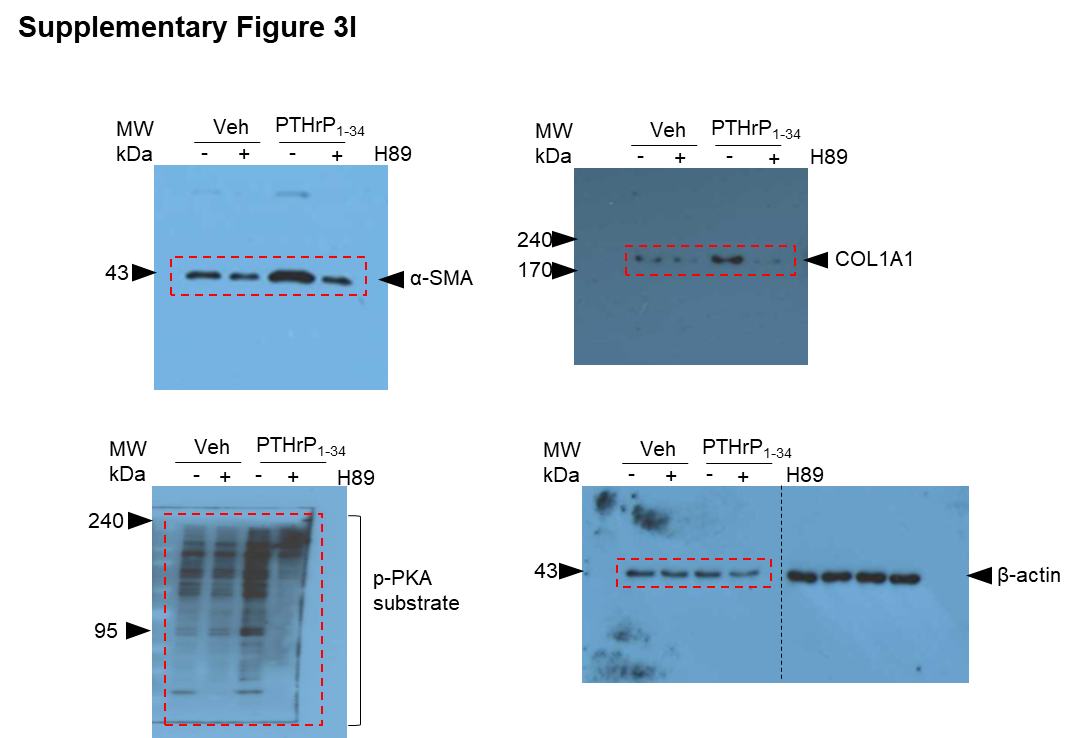


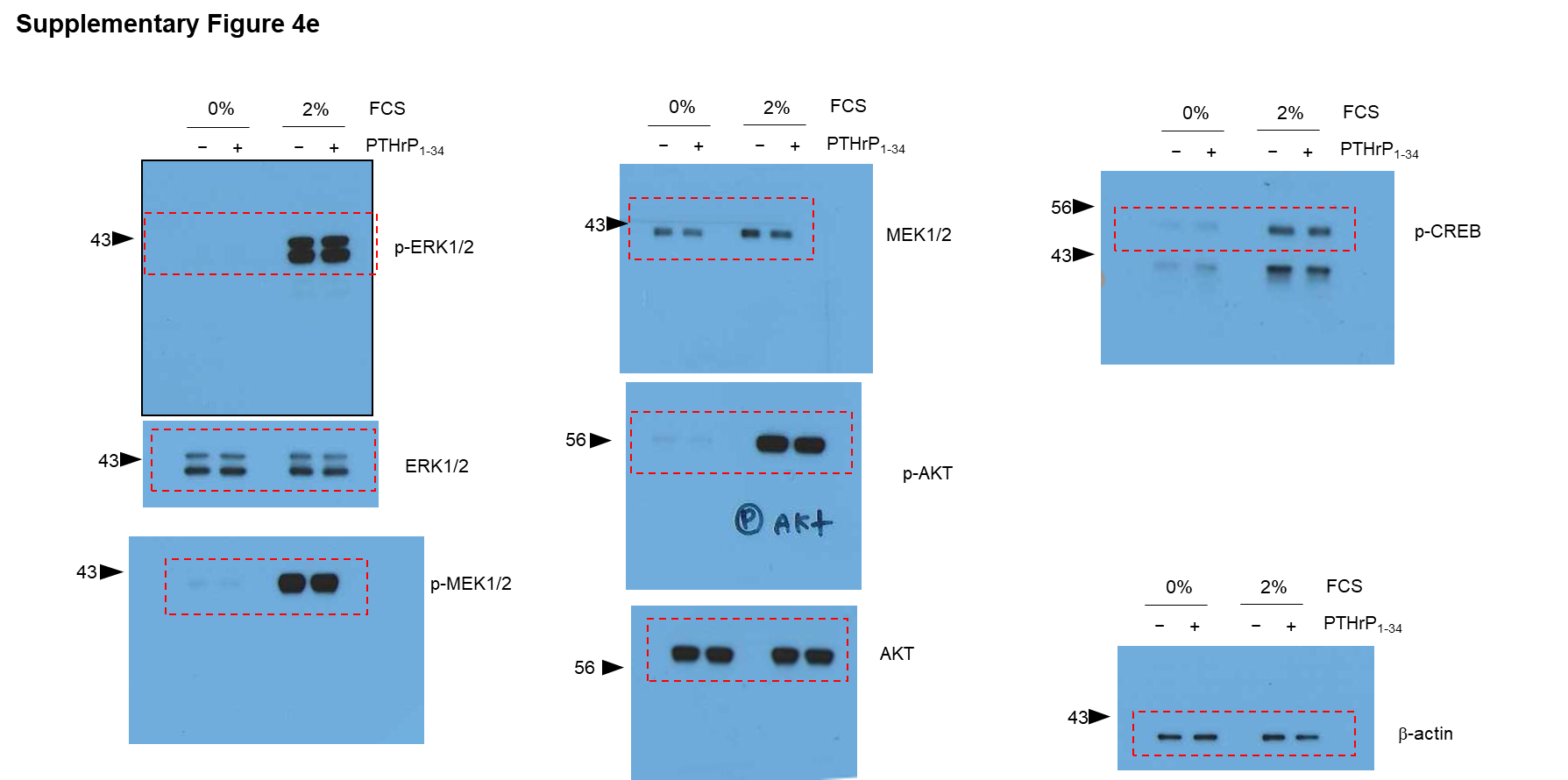


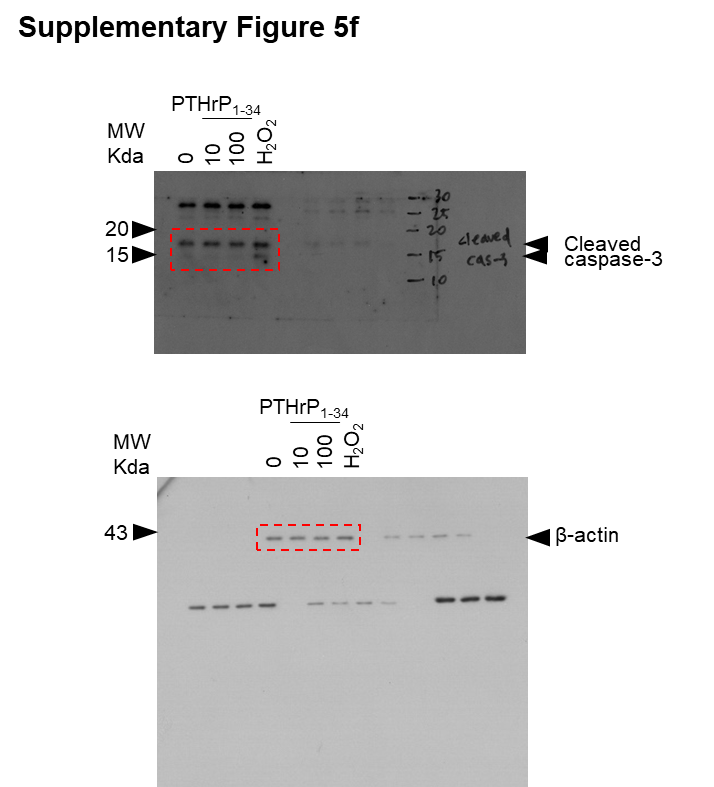


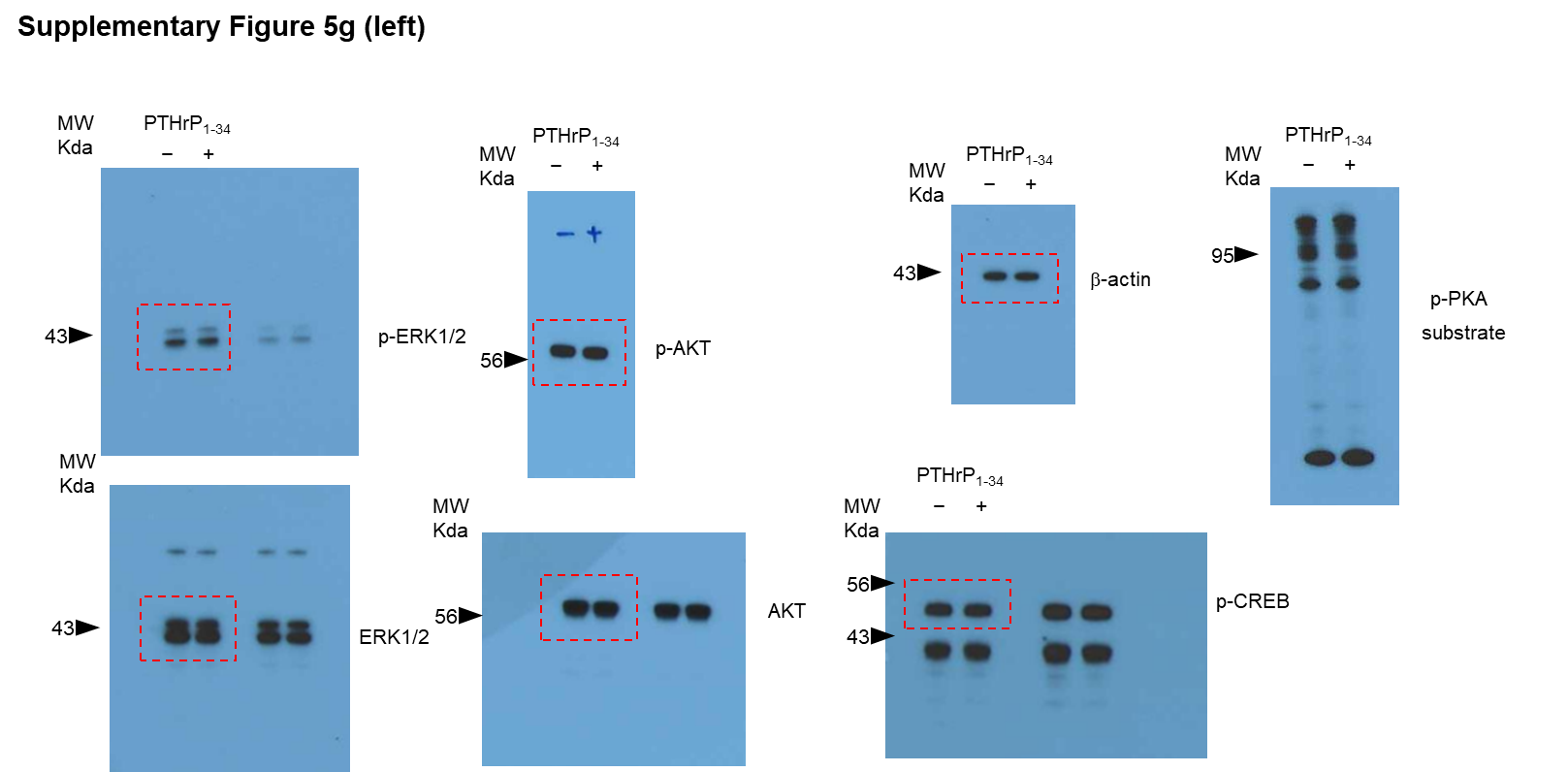


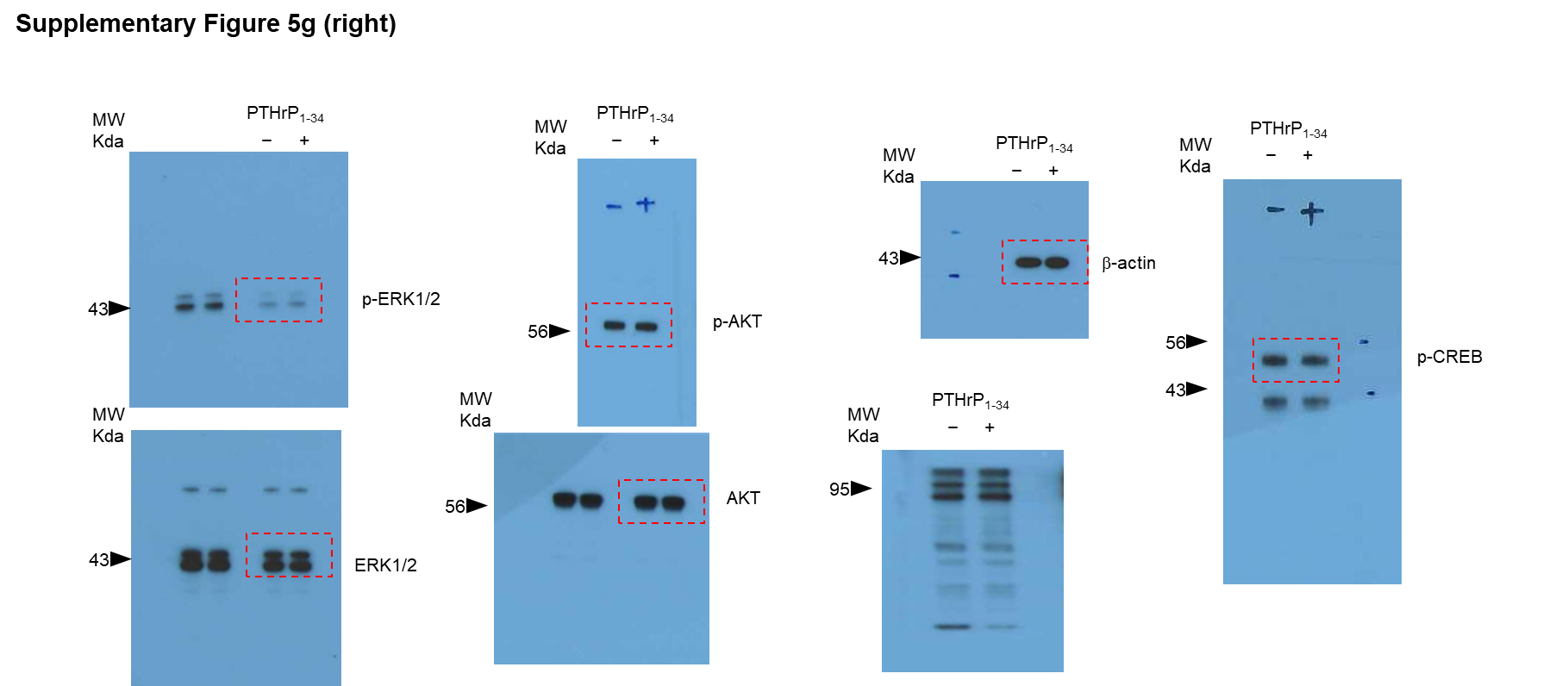


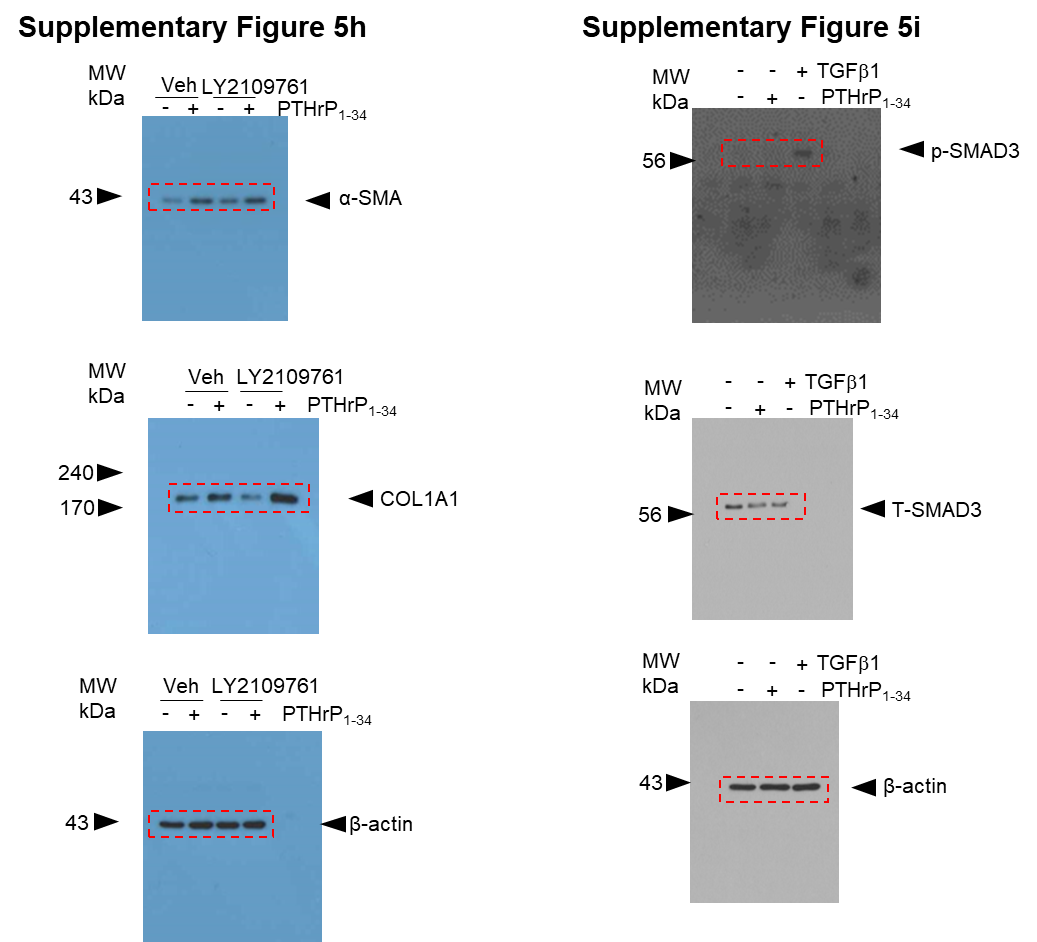


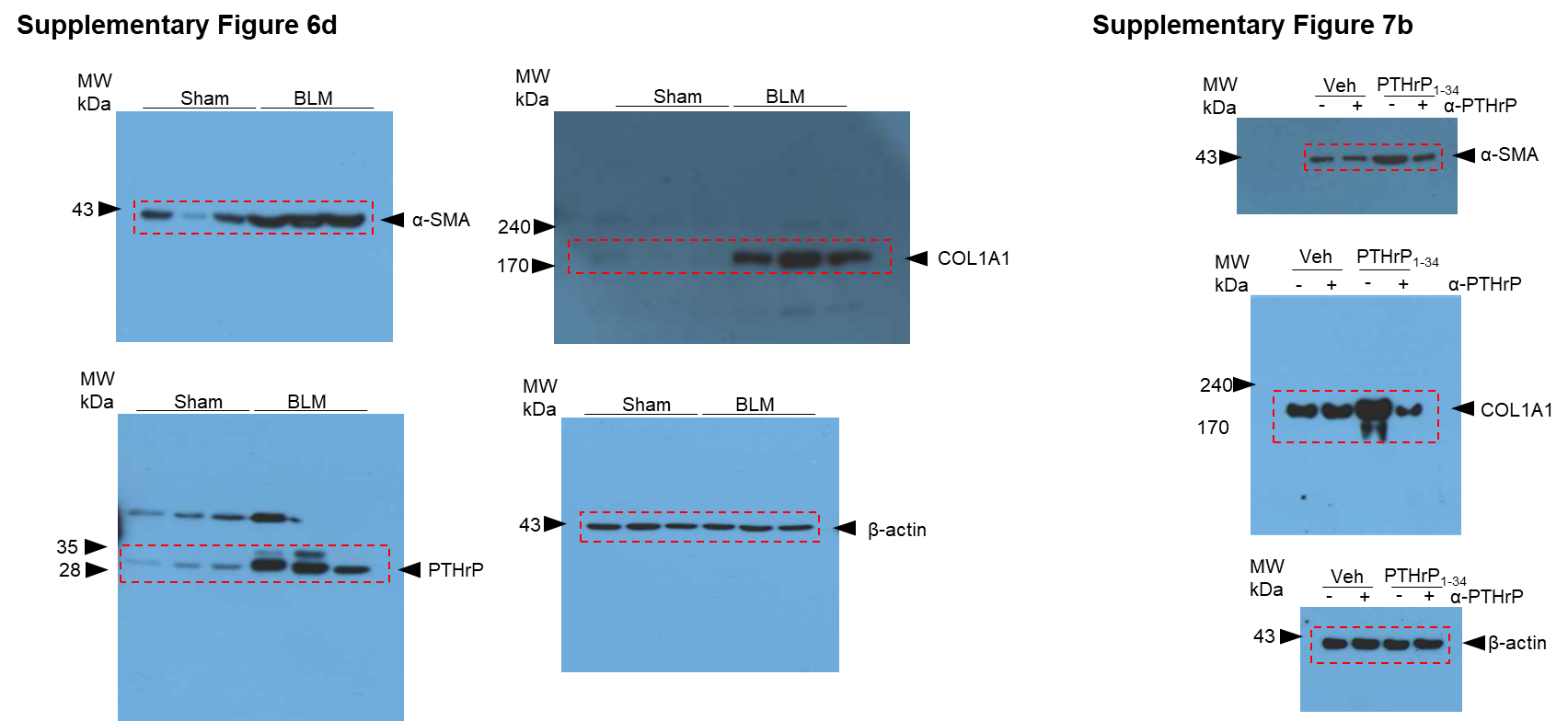


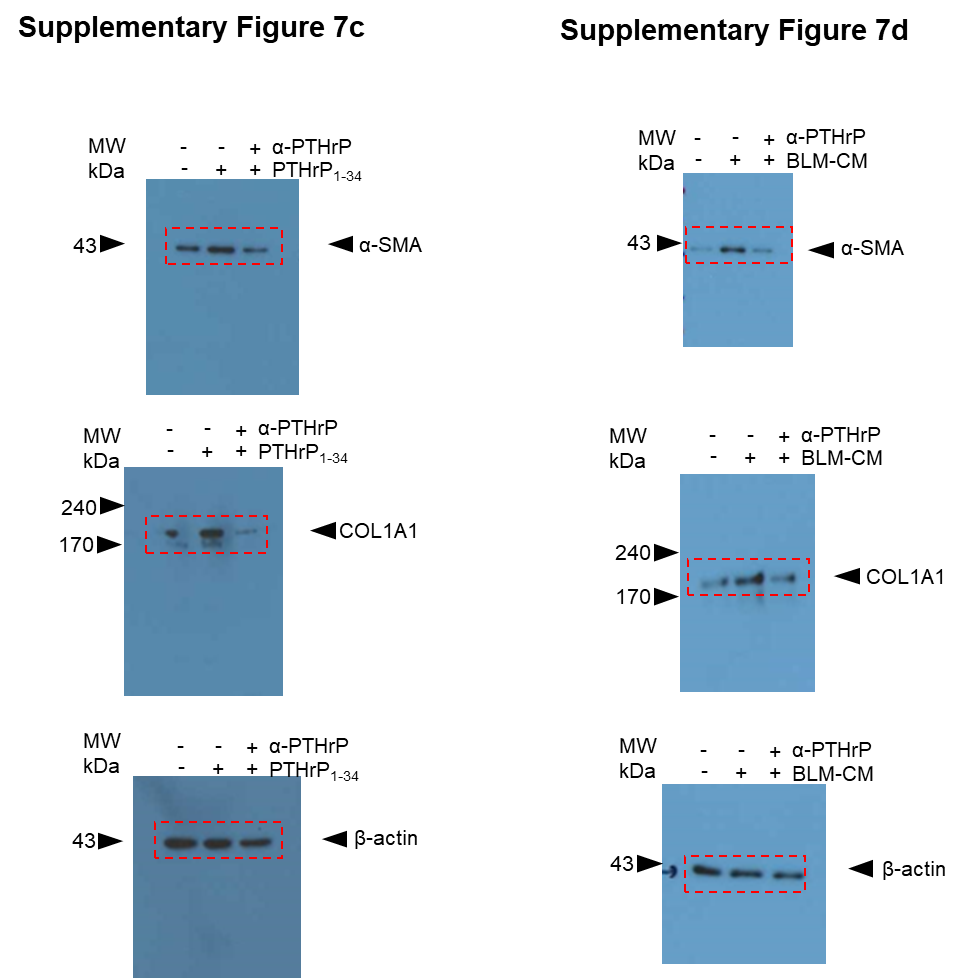


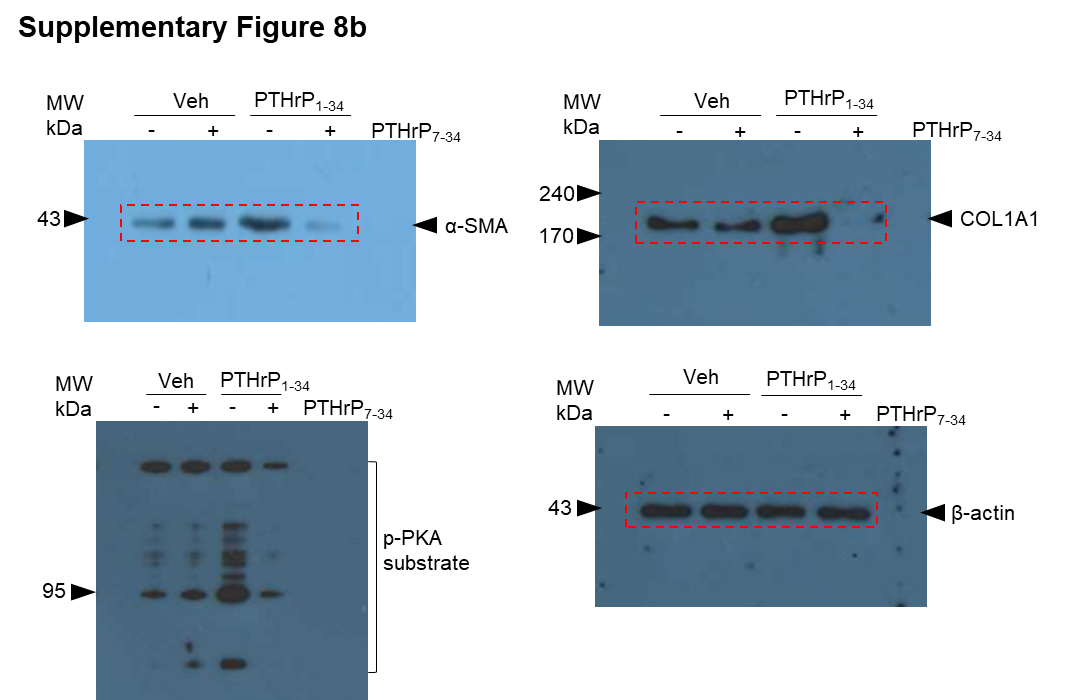


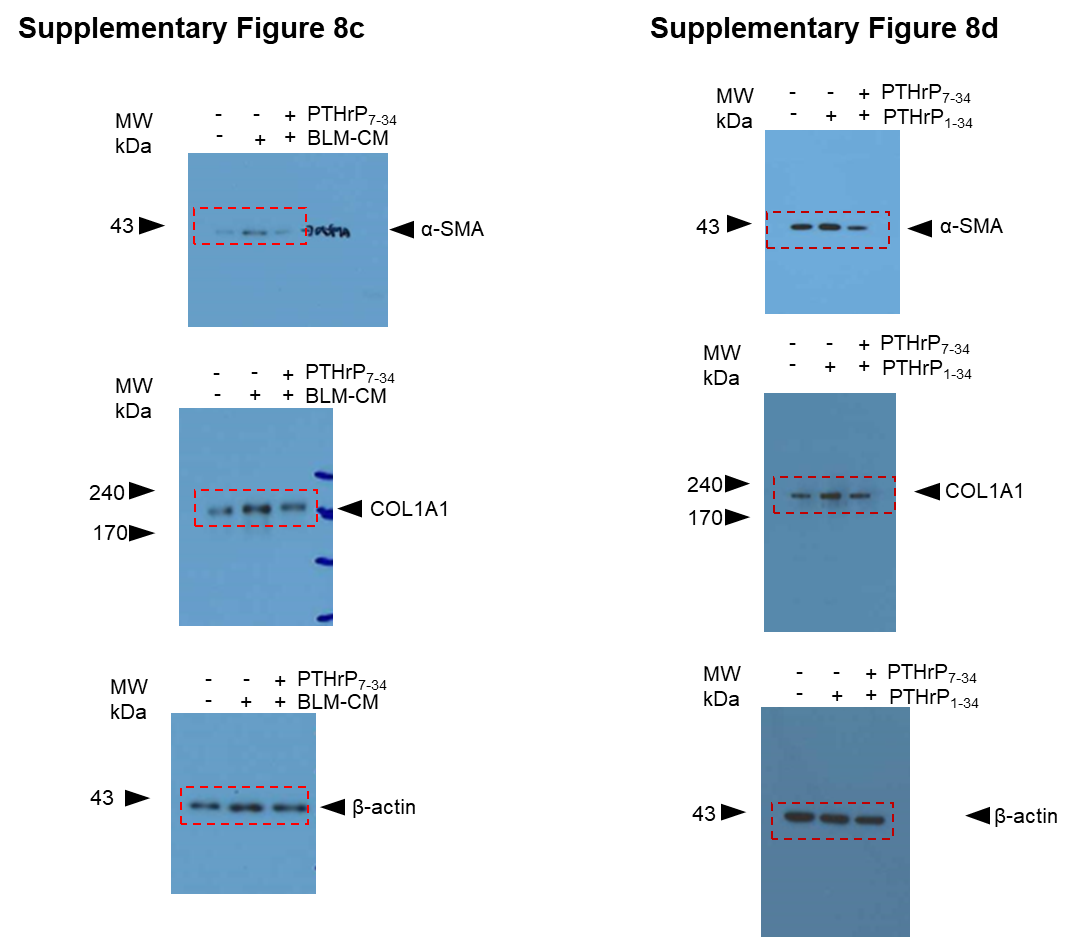

Supplement: Supplementary file 1 — Supplementary materials [file 41392_2026_2578_MOESM1_ESM.docx]
